# Modulating the Nitrate Reduction Pathway on Unconventional Phase Ultrathin Nanoalloys for Selective Ammonia Electrosynthesis

**DOI:** 10.1021/jacs.5c07490

**Published:** 2025-06-20

**Authors:** Jingwen Zhou, Fu Liu, Zhihang Xu, Jian-An Yin, Liang Guo, Fengkun Hao, Yunhao Wang, Yuecheng Xiong, Xichen Zhou, Cheng Wang, Yangbo Ma, Xiang Meng, Pengyi Lu, Jinwen Yin, An Zhang, Jie Wang, Chenliang Ye, Qiang Li, Chongyi Ling, Hsiao-Chien Chen, Hao Ming Chen, Ye Zhu, Jian Lu, Zhanxi Fan

**Affiliations:** 1 Department of Chemistry, 53025 City University of Hong Kong, Kowloon, Hong Kong SAR 999077, China; 2 Department of Applied Physics, Research Institute for Smart Energy, 26680 The Hong Kong Polytechnic University, Kowloon, Hong Kong SAR 999077, China; 3 Hong Kong Branch of National Precious Metals Material Engineering Research Center (NPMM), City University of Hong Kong, Kowloon, Hong Kong SAR 999077, China; 4 Department of Mechanical Engineering, City University of Hong Kong, Kowloon, Hong Kong SAR 999077, China; 5 Key Laboratory of Fluid and Power Machinery of Ministry of Education, School of Materials Science and Engineering, 12598 Xihua University, Chengdu, 610039 Sichuan, China; 6 Department of Power Engineering, 47840 North China Electric Power University, Baoding, 071003 Hebei, China; 7 Key Laboratory of Quantum Materials and Devices of Ministry of Education, School of Physics, Southeast University, Nanjing 211189, China; 8 Center for Reliability Science and Technologies, Center for Sustainability and Energy Technologies, 56081 Chang Gung University, Taoyuan 33302, Taiwan; 9 Department of Chemistry, 33561 National Taiwan University, Taipei 10617, Taiwan; 10 Hong Kong Institute for Clean Energy, City University of Hong Kong, Kowloon, Hong Kong SAR 999077, China; 11 City University of Hong Kong, Shenzhen Research Institute, Shenzhen 518057, China

## Abstract

Ammonia (NH_3_) electrosynthesis from nitrate-polluted wastewater is a challenging but meaningful technique for the future green chemical and sewage disposal industries. The dominant difficulties lie in how to realize a highly selective, low-overpotential, and rapid electrocatalytic nitrate reduction reaction (NO_3_RR). Herein, we propose a catalyst crystal phase and electrode/electrolyte interface dual engineering strategy to enhance the neutral NO_3_RR performance of ultrathin alloy nanostructures. The obtained unconventional 2H-RhCu not only shows higher intrinsic NH_3_ selectivity than its traditional face-centered cubic and amorphous/crystalline counterparts but also delivers superior Faradaic efficiency and yield rate toward NH_3_ in K^+^-based electrolyte over those in Li^+^/Na^+^-based ones. *In situ* studies and theoretical calculations reveal that the faster generation/conversion kinetics of key intermediates, weaker N–N recombination, and unique *NO_bri_ adsorption configuration at electrode/electrolyte interfaces account for this significant enhancement. In addition, rechargeable Zn-nitrate/methanol flow batteries with 2H-RhCu were constructed as a demonstration of potential applications.

## Graphical abstract

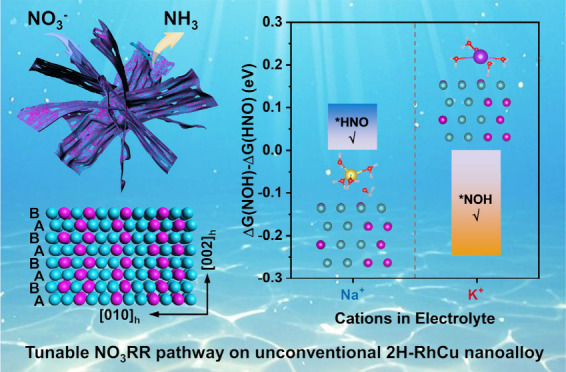

## Introduction

As a dominant pollutant in industrial wastewater, nitrate (NO_3_^–^) has caused considerable environmental concerns over the past century, like eutrophication and health risks to drinking water.^1,2^ How to efficiently eliminate NO_3_^–^ to reach a low nitrogen concentration that is safe for release to the environment is an urgent pursuit for sewage disposal industries. Current aqueous NO_3_^–^ degradation heavily relies on ion exchange and biological denitrification methods to convert nitrate nitrogen to harmless N_2_ gas for emission and faces issues of energy-intensive production, tedious process procedures, and high-cost equipment and consumables.^3,4^ Generating N_2_ as the final NO_3_^–^ degradation product conforms to environmental protection, but it is not energy-economical enough from the perspective of sustainable development.^5,6^ Interestingly, rapid technical advances in electrochemical synthesis in recent years offer another promising disposal route to upgrade the NO_3_^–^ contaminant to high-value-added chemicals for wide applications, like ammonia (NH_3_)^7–9^ or even organic ammonium salts after coupling with appropriate CO_2_/biomass-derived clean fuels.^10–13^ Such a “turning waste to wealth” electrosynthesis strategy greatly appeals to future green chemistry and sewage disposal industries,^14–16^ but there are still considerable issues that obstruct its development.

The dominant technical hurdles hindering the industrialization of this NO_3_^–^ upgrading route lie in the following two difficulties. On the one hand, it is challenging for current metal nanocatalysts to achieve a highly selective and fast electrocatalytic nitrate reduction reaction (NO_3_RR) toward NH_3_ formation at low overpotentials, especially in neutral media with low corrosiveness. Most previously reported metal catalysts (e.g., the commonly used copper (Cu)) delivered acceptable Faradaic efficiency (FE) toward NH_3_ (FE­(NH_3_)) only when the applied potential was more negative than −0.7 V (vs. reversible hydrogen electrode (RHE)), resulting in quite limited energy efficiency for NH_3_ electrosynthesis.^17–22^ Among different metal elements that have been attempted for NO_3_RR, Rh^23^ and its alloys like RhCu^14,24^ and RhNi^25^ exhibit the specific ability to realize sufficient FE­(NH_3_) at much less negative potentials (e.g., greater than −0.3 V vs. RHE) through decreasing the energy barriers of intermediate deoxygenation and hydrogenation steps.^23,26,27^ However, Rh-based catalysts encounter a non-negligible issue of low NH_3_ yield rates, due to the strong hydrogen evolution competition and sluggish *k*_1_ (NO_3_^–^ to NO_2_^–^) reaction kinetics on Rh sites during NO_3_RR.^26,27^ Restricted by the intrinsic properties of common face-centered cubic (fcc) Rh crystals, those optimization methods focusing on traditional factors like geometrical shape, alloying, size, and catalyst distribution seem to face difficulty breaking through this dilemma.^28–31^ In comparison, the potential of phase control of electrocatalysts has been barely unraveled in this emerging direction.^32,33^ On the other hand, most research attention has been devoted to catalyst optimization, while there is an extreme lack of studies regarding the effects of the solvation structure of the electrolyte on NO_3_RR performance and its underlying electrochemical mechanisms, despite the fact that alkali metal cations in the electrolytes have proven to be highly effective in regulating the electrochemical behavior of various aqueous electrocatalytic reactions including but not limited to the electrocatalytic CO_2_ reduction reaction.^34–37^ Understanding what happens at the electrode/electrolyte interfaces in different electrolytes and designing appropriate cation solvation structures to match with the used catalysts accordingly are of great significance for accelerating the industrialization of the NO_3_RR.

Herein, we report a catalyst crystal phase and electrode/electrolyte interface dual engineering strategy for ultrathin rhodium–copper (RhCu) alloy nanostructures to significantly facilitate the electroreduction of NO_3_^–^ to NH_3_ in neutral media. Common amorphous/crystalline (A/C)- and fcc- and unconventional 2H-RhCu nanostructures with similar morphology and composition have been synthesized via a one-pot wet-chemical method. Impressively, 2H-RhCu delivers superior selectivity and single-site activity toward NH_3_ over its A/C and fcc counterparts, because it more favorably activates critical intermediates like *NO, *HNO, and *NH_2_OH as well as blocks undesirable N–N recombination. Furthermore, cation effects allow 2H-RhCu to demonstrate the highest FE­(NH_3_) of 94.8% at −0.3 V (vs. RHE) and the largest yield rate of 4936.8 mg h^–1^ g^–1^_cat_ in the K^+^-based electrolyte, greatly exceeding those in Li^+^/Na^+^-based environments. *In situ* experimental characterization and theoretical simulation results determined that the K^+^-filled outer Helmholtz plane (OHP) stabilizes the *NO_bri_ more than the commonly seen *NO_on‑top_ adsorption configuration at the electrode/electrolyte interface, which leads to faster hydrogenation by a *NH_2_OH-free pathway. As a multifunctional device prototype combining energy storage, sewage disposal, and electrosynthesis, the constructed Zn-nitrate/methanol flow battery using the 2H-RhCu nanocatalyst shows a low discharge–charge overpotential gap of 0.55 V at 0.5 mA cm^–2^ and produces value-added HCOONH_4_ in the catholyte after long-term cycling.

## Results and Discussion

### Phase Modulation of Ultrathin RhCu Nanostructures

The phase control of ultrathin RhCu solution alloy nanostructures has been achieved by varying the solvents and reaction temperatures in the hydro­(solvo)­thermal growth, as schematically depicted in Figure 1a. In general, formaldehyde (HCHO) was used as the reductant and capping agent (i.e., carbon monoxide) precursor to induce the generation of ultrathin nanostructures in all three synthetic routes. The extra introduction of oleylamine (OAm) and oleic acid (OLA) as the ligands results in preferable nucleation and growth toward the 2H phase, in contrast to the preparation conditions for common fcc-RhCu. By lowering the reaction temperature to reduce the crystallinity of fcc domains, we can obtain A/C-RhCu. The preparation details are provided in the Supporting Information.

**Figure 1:**
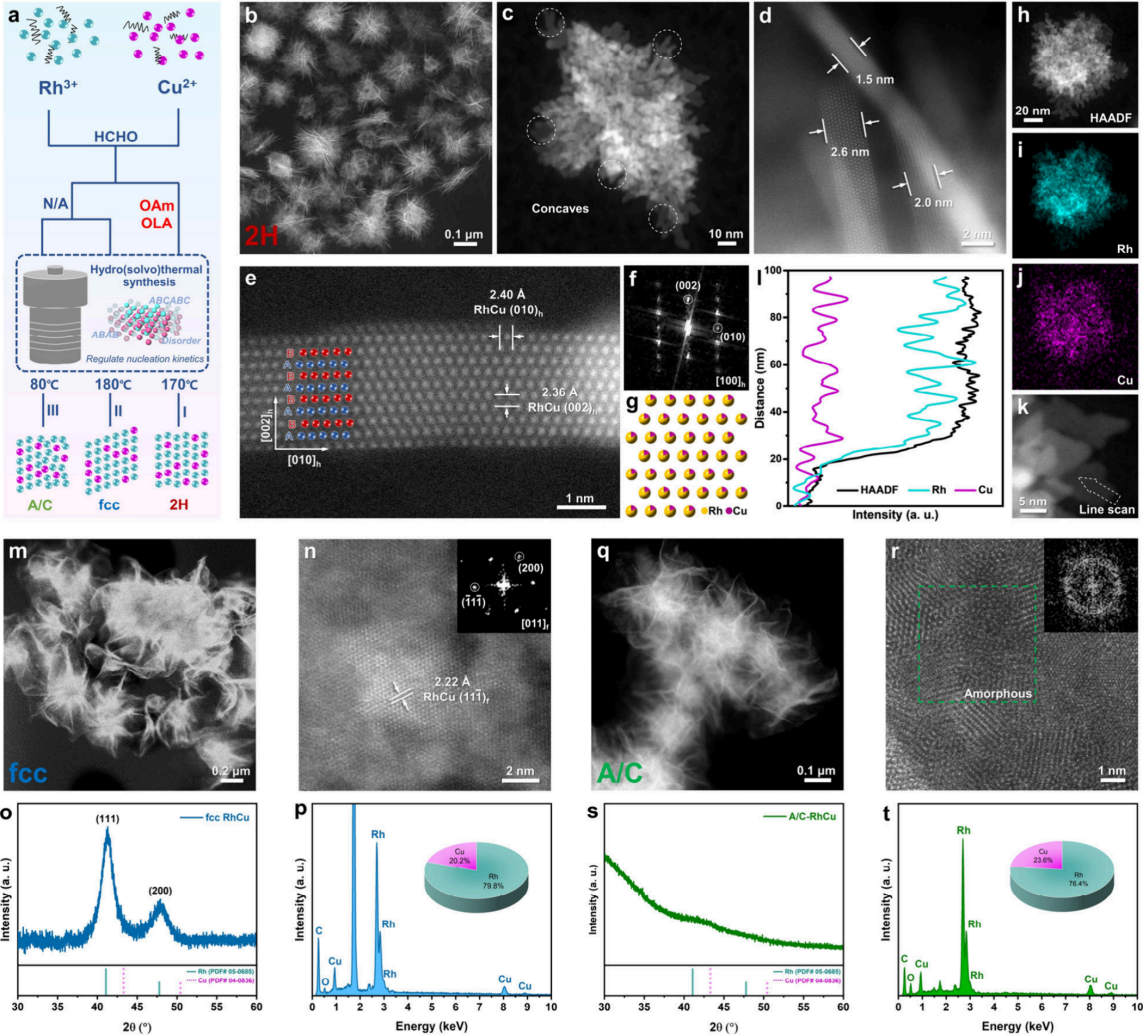
Synthesis and structural characterizations of RhCu alloy nanostructures with different phases. (a) Schematic illustration of the synthesis of ultrathin A/C-, fcc-, and 2H-RhCu nanosheet assemblies. (b–d) HAADF-STEM images showing the morphology (b), concave edges (c), and thickness (d) of 2H-RhCu nanostructures. (e–g) Atomic-resolution HAADF-STEM image (e), FFT pattern (f), and simulated atomic model (g) of a single 2H-RhCu nanosheet along the [100]_h_ direction. (h–j) HAADF-STEM image (h) and corresponding EDS elemental maps of Rh (i) and Cu (j) for a 2H-RhCu nanosheet assembly. (k, l) HAADF-STEM image (k) and EDS line-scan profiles (l) across the basal plane of a single 2H-RhCu nanosheet indicated by the white dashed arrow marked in (k). (m–p) Low-magnification HAADF-STEM image (m), atomic-resolution HAADF-STEM image (n) with the corresponding FFT pattern as inset, XRD pattern (o), and EDS spectrum (p) of fcc-RhCu nanostructures. (q–t) Low-magnification HAADF-STEM image (q), atomic-resolution HAADF-STEM image (r) with the corresponding FFT pattern as inset, XRD pattern (s), and EDS spectrum (t) of A/C-RhCu nanostructures.

The microstructures of as-synthesized RhCu nanostructures were first investigated by transmission electron microscopy (TEM), high-resolution TEM (HRTEM), and atomic-resolution aberration-corrected high-angle annular dark-field scanning TEM (HAADF-STEM). The unconventional 2H-RhCu shows a morphology of nanourchins assembled from randomly oriented ultrathin nanosheets (Figures 1a and S1a,b). The corresponding selected-area electron diffraction (SAED) pattern indicates that they are polycrystalline with a good 2H phase purity (Figure S2a,b). When the nanosheets were examined in detail, concave areas were frequently observed at the edges of their basal planes (Figures 1c and S2c). Their thickness shows a narrow distribution from 1.5 to 3.5 nm (Figures 1d and S1c). According to the HRTEM and zoom-in HAADF-STEM characterizations shown in Figures S1d–g and Figure 1e–g, these nanosheets demonstrate a typical hexagonal close-packed (hcp, 2H type) phase with the stacking pattern of “AB” along the [001]_h_ close-packed direction. The adjacent lattice distances are measured to be 2.40 and 2.36 Å for (010)_h_ and (002)_h_ facets, respectively (Figures 1e and S2d,e). A pivotal bright spot belonging to (010) facets of 2H crystals was found in the corresponding selected-area fast Fourier transform (FFT) pattern along the [100]_h_ zone axis (Figure 1f and S1f), further confirming the formation of the 2H phase. This is further supported by the unique XRD pattern of 2H-RhCu, which shows significant differences from the standard patterns ascribed to common fcc-Rh and fcc-Cu (Figure S1h). Due to the uniform contrast, Cu atoms should lie randomly in the Rh lattices, thus producing structurally disordered solid solution alloys. Based on the above characterizations, a simulated atomic model of 2H-RhCu is provided in Figure 1g for reference. Besides, according to the energy-dispersive X-ray spectroscopy (EDS) result (Figure S3), the atomic ratio of Rh/Cu is 76.0/24.0 in 2H-RhCu. The corresponding EDS elemental maps (Figure 1h–j) and line scans (Figure 1k,l) identify that Rh and Cu are homogeneously dispersed in these nanosheet assemblies. Time-dependent experiments indicate that RhCu clusters should be first generated in the crystal growth of 2H-RhCu, and then OAm and OLA ligands are able to adjust the surface energy of these clusters to stabilize at a state preferentially growing along the AB stacking sequence. As the capping agent, CO from HCHO decomposition strongly adsorbed onto the (002) planes prevents the thickening of the nanocrystal nucleus and induces the formation of nanosheets. The crystallinity of 2H-RhCu was further improved in the following heat preservation process (Figure S4).

For fcc-RhCu, ultrathin nanosheets are interconnected to constitute nanoflowers (Figures 1m and S5a,b). These nanoflowers possess a high fcc phase purity according to the SAED pattern in Figure S5a. The zoomed-in HAADF-STEM image together with the corresponding FFT pattern give proof that the (011)_f_ facet is the basal plane of fcc-RhCu nanosheets (Figure 1n). Combining the XRD pattern (Figure 1o) and EDS spectrum (Figure 1p), the as-synthesized fcc-RhCu is also a solid solution alloy in which Rh atoms occupy about 80% of fcc sites and Cu atoms fill in the remaining sites. In addition, no apparent Rh/Cu element segregation was observed in the nanosheets of fcc-RhCu (Figure S5c–f). The obtained A/C-RhCu demonstrates a similar morphology and composition to those of fcc-RhCu (Figures 1q,t and S6a), but its crystallinity is much lower (Figure 1r,s), and numerous amorphous domains are formed within the two-dimensional (2D) in-plane structures (Figure 1r). The thicknesses of the nanosheet building blocks for fcc- and A/C-RhCu are both 2–3 nm on average (Figures S5b and S6b).

X-ray photoelectron spectroscopy (XPS) was utilized to unravel the electronic structure discrepancy of the as-synthesized RhCu alloys. The Rh 3d XPS spectra show two groups of subpeaks located at 307.3/312.1 eV and 308.4/313.7 eV (Figure 2a), which are attributed to Rh^0^ and Rh^3+^, respectively.^28,38,39^ Remarkably, metallic Rh dominates the surface of 2H-RhCu while there is a higher proportion of Rh^3+^ in fcc-RhCu and A/C-RhCu. Based on the Cu 2p XPS spectra (Figure 2b), metallic Cu^0^ (931.5/951.5 eV) dominates in all three samples, but A/C-RhCu has a larger proportion of Cu^+^ and Cu^2+^ (933.8/953.4 eV) in contrast to its 2H and fcc counterparts.^40–42^ These Rh/Cu oxidation states should originate from the surface oxidation of ultrathin nanostructures exposed in air.

**Figure 2:**
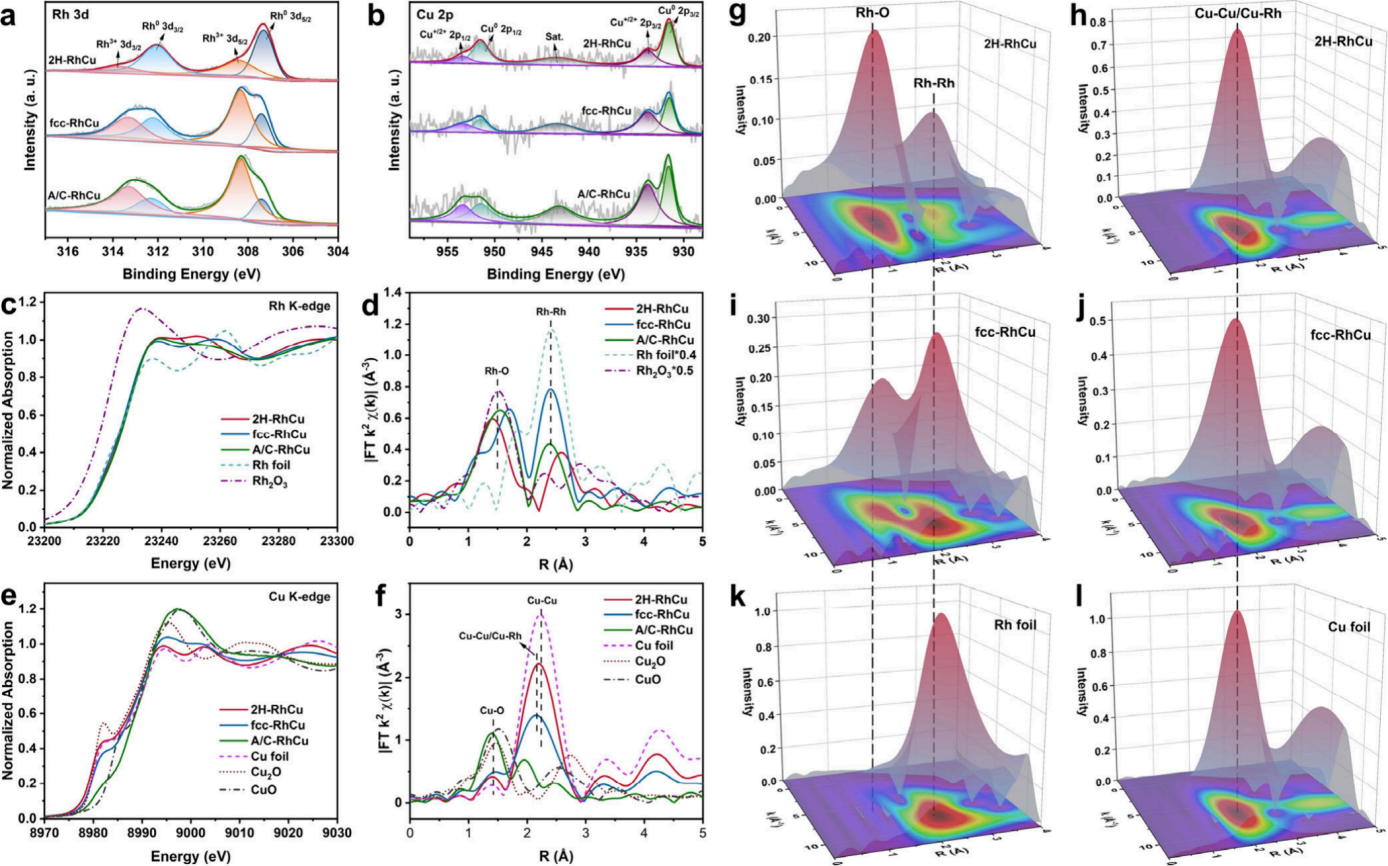
X-ray spectral analysis. (a, b) High-resolution XPS spectra of Rh 3d (a) and Cu 2p (b) orbits for A/C-, fcc-, and 2H-RhCu nanostructures. (c, d) Normalized Rh K-edge XANES (c) and R-space EXAFS (d) spectra of A/C-, fcc-, and 2H-RhCu nanostructures. Reference samples of Rh foil and Rh_2_O_3_ are included for comparison. (e, f) Normalized Cu K-edge XANES (e) and R-space EXAFS (f) spectra of A/C-, fcc-, and 2H-RhCu nanostructures. Reference samples of Cu foil, Cu_2_O and CuO are included for comparison. (g–l) Contour plots of wavelet transform of Rh (g, i) and Cu (h, j) K-edge EXAFS data for 2H- (g, h) and fcc-RhCu (i, j) nanostructures. Reference samples of Rh (k) and Cu (l) foils are included for comparison.

To further analyze the coordination environments of these RhCu nanocatalysts with different phases, X-ray absorption near-edge structure (XANES) and extended X-ray absorption fine structure (EXAFS) spectroscopies were used. From the XANES spectra at Rh K-edge (Figure 2c), 2H-, fcc- and A/C-RhCu exhibit similar white line positions which are very close to that of Rh foil rather than that of Rh_2_O_3_, suggesting Rh atoms are mostly in metallic states for all three RhCu alloys. After investigating the Fourier transforms of EXAFS spectra (Figure 2d), we found that fcc- and A/C-RhCu and Rh foil exhibit dominant peaks at 2.41 Å in R space, ascribed to the Rh–Rh scattering path.^43–45^ In comparison, this peak for 2H-RhCu moved forward to 2.59 Å. The differences in Rh–Rh bond length indicate the formation of unconventional 2H Rh substrates. Simultaneously, the coordination numbers (CNs) of Rh atoms are 6.6 for fcc-RhCu and 6.0 for 2H-RhCu (Figure S7a,c,e and Table S1), which suggests that there are plenty of low-coordination Rh atoms exposed on the surface owing to the ultrathin nature of as-synthesized 2D nanostructures. In the XANES spectra at the Cu K-edge (Figure 2e), 2H- and fcc-RhCu show similar white line positions and intensities to those of Cu foil, while the data for A/C-RhCu are approaching those for CuO. This result is in good accordance with the previous XPS analysis. At R space (Figures 2f and S7b,d,f), different from the dominant peak at 2.24 Å (Cu–Cu scattering path) for Cu foil, 2H- and fcc-RhCu present dominant peaks at 2.19 and 2.15 Å, respectively, ascribed to the Cu–Cu and/or Cu–Rh scattering paths of the first shell.^46–48^ Alloy structure and phase diversity should account for such metal–metal bond length changes. In addition, according to the wavelet transform (WT) of the EXAFS spectra (Figure 2g,i,k), the center of maximum intensity for the Rh–Rh path of 2H-RhCu has a slight increase in R range but a decrease in K range, compared with those for fcc-RhCu and Rh foil. In terms of Cu–Cu/Cu–Rh paths, their contour plots of WT are similar (Figure 2h,j,l), but 2H-RhCu reveals a narrow maximum intensity distribution in both K and R ranges, in contrast to the fcc one. The detailed fitting results are provided in Table S1, for more comprehensive information on their fine coordination structures.

### Phase-Dependent NH_3_ Selectivity and NO_3_RR Performance Improvement via Cation Effects in Neutral Media

The electrocatalytic NO_3_RR performance was first evaluated in a frequently used neutral solution consisting of 0.5 M Na_2_SO_4_ and 0.1 M NaNO_3_ to avoid CO_2_ intake and corrosion issues as much as possible. The NO_3_RR products were quantified by colorimetric methods (Figure S8). As seen from the linear sweep voltammetry (LSV) curves (Figure S9), distinctly greater current densities in the presence of NO_3_^–^ over the ones in the absence of NO_3_^–^ confirm that all of the synthesized RhCu nanocatalysts are active for NO_3_RR below 0 V (vs. RHE), but the competing hydrogen evolution reaction (HER) becomes severe when the applied potential is less than −0.4 V (vs. RHE). Therefore, we have further checked the FEs and yield rates of NH_3_ and NO_2_^–^, the two major products of the NO_3_RR, over the potential window of 0 to −0.4 V (vs. RHE) (Figure 3a). Despite fcc-RhCu showing the larger current densities during tests, its selectivity toward NH_3_ is much inferior to that of 2H-RhCu. When the potential is above −0.3 V (vs. RHE), 2H-RhCu demonstrates good FE­(NH_3_), and the highest value of 81.1% is obtained at −0.2 V (vs. RHE) while its FE­(NO_2_^–^) is limited to a quite low level, ranging from 1.16 to 5.96%. In comparison, fcc-RhCu exhibits much higher FE­(NO_2_^–^), among which the largest one reaches 44.4% at −0.3 V (vs. RHE). The FE­(NH_3_) for fcc-RhCu only fluctuates at around 50%. For A/C-RhCu, the NH_3_ selectivity is even worse. When analyzing the FE­(NH_3_)/(FE­(NH_3_) + FE­(NO_2_^–^)) ratios (Figure 3b), the advantages of 2H-RhCu are more obvious. These values are always above 0.9 for 2H-RhCu but much lower (0.4 to 0.7) for fcc and A/C RhCu.

**Figure 3:**
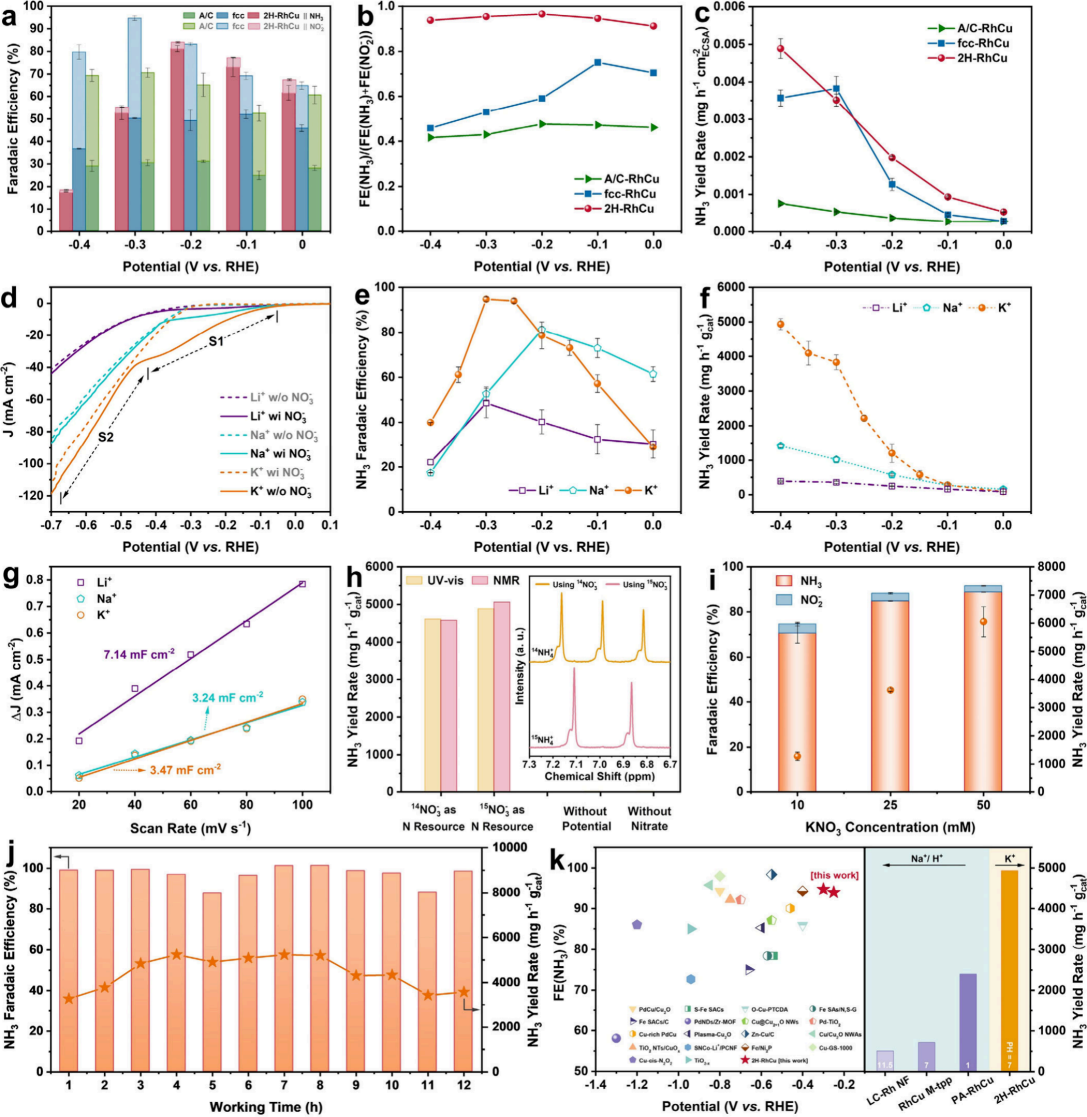
NO_3_RR performance in neutral media of the as-synthesized ultrathin RhCu nanostructures with different phases. (a–c) FE­(NH_3_) and FE­(NO_2_^–^) (a), $\frac{\mathrm{FE}\left(NH_{3}\right)}{\mathrm{FE}\left({NO_{2}}^{-}\right)+\mathrm{FE}\left(NH_{3}\right)}$ ratios (b), and NH_3_ yield rates based on ECSA (c) of A/C-, fcc-, and 2H-RhCu nanostructures in 0.5 M Na_2_SO_4_ + 0.1 M NaNO_3_ solution. (d) LSV curves at 5 mV s^–1^ of 2H-RhCu cathodes in 0.5 M Li^+^, Na^+^, and K^+^ based neutral media in the absence and presence of 100 mM NO_3_^–^ over the potential window of 0.1 to −0.7 V (vs. RHE). (e, f) FE­(NH_3_) (e) and NH_3_ yield rate (f) of 2H-RhCu in Li^+^, Na^+^, and K^+^ based electrolytes at different potentials. (g) Fitting results of *C*_dl_ for 2H-RhCu in Li^+^, Na^+^, and K^+^ based electrolytes. (h) NH_3_ yield rates of 2H-RhCu cathodes in K^+^-based electrolyte using ^14^NO_3_^–^ or ^15^NO_3_^–^ as the nitrogen resource at −0.3 V (vs. RHE), quantified by both UV–vis and NMR methods. The yield rates recorded in the cases without applied potential but in the presence of NO_3_^–^ and without NO_3_^–^ at −0.3 V (vs. RHE) are also provided. Inset: ^1^H NMR spectra of electrolytes after NO_3_RR using ^14^NO_3_^–^ or ^15^NO_3_^–^ as nitrogen source. (i) FE­(NH_3_) and FE­(NO_2_^–^), and NH_3_ yield rate of 2H-RhCu in K^+^-based solutions with low-concentration NO_3_^–^. (j) Cycling stability of 2H-RhCu in 0.5 M K_2_SO_4_ + 0.1 M KNO_3_ solution at −0.3 V (vs. RHE). (k) Comparison of electrochemical performance of 2H-RhCu in this work and other representative NO_3_RR catalysts reported previously.

Although fcc-RhCu demonstrates larger current densities in the chronoamperometry (CA) tests (Figure S10), it does not show remarkable advantages on NH_3_ partial current densities and NH_3_ yield rates (Figure S11), restricted by its own unsatisfactory FE­(NH_3_). There are similar NH_3_ yield rates based on the weight of catalyst for 2H- and fcc-RhCu when the potential is above −0.2 V (vs. RHE). Compared with the two crystalline counterparts, A/C-RhCu shows negligible NH_3_ yield rates. These yield rate differences may be attributed to the different electrochemically active surface areas (ECSAs) of as-synthesized RhCu nanostructures to a certain degree since fcc-RhCu demonstrates a much larger *C*_dl_ of 5.47 mF cm^–2^ over its 2H (3.24 mF cm^–2^) and A/C (1.06 mF cm^–2^) counterparts (Figure S12). After the NH_3_ yield rates are normalized to ECSAs, 2H-RhCu even exhibits larger values than fcc-RhCu (Figure 3c), suggesting that 2H-RhCu has the intrinsically faster reaction kinetics for NH_3_ electrosynthesis on individual active sites. In terms of the energy efficiency toward NH_3_ (Figure S13), 2H-RhCu is superior to both fcc and A/C ones when the applied potential is higher than −0.3 V (vs. RHE). Besides, 2H-RhCu also shows larger turnover frequencies of NH_3_ (TOF_NH_3__) over the potential window (Figure S14). On basis of the aforementioned results, the 2H phase is regarded as the most favorable crystal structure of RhCu nanoalloys for neutral NO_3_RR.

After determining the most favorable phase, we have further tried to enhance the electroreduction of NO_3_^–^ to NH_3_ via cation effects which can lead to obvious structural distinctions of the OHP at electrode/electrolyte interfaces.^49–51^ Here, three kinds of commonly used alkali metal cations including Li^+^, Na^+^, and K^+^ were investigated. As observed from the LSV curves (Figure 3d), there are two different regions marked by S1 and S2, where S1 is the NO_3_RR-dominant process, while S2 is the HER-dominant process. At the S1 region, the 2H-RhCu cathode realized the lowest Tafel slope of 278 mV dec^–1^ in K^+^-based solution and its current densities showed a rising tendency obeying the order of K^+^ > Na^+^ > Li^+^ in the presence of NO_3_^–^ (Figure S15). More importantly, in contrast to the situation in Na^+^-based solution, the highest FE­(NH_3_) increased to 94.8% in K^+^-based solution, but it decreased to 48.5% in Li^+^-based solution at −0.3 V (vs. RHE) (Figure 3e). The corresponding maximum energy efficiencies of NH_3_ production are 30.28%, 54.15%, and 60.61% in the Li^+^, Na^+^ and K^+^-based neutral electrolytes, respectively (Figure S16). In addition, the Faradaic efficiencies toward H_2_ (FE­(H_2_)) are 5.4% and 22.2% at −0.3 and −0.4 V (vs. RHE) in K^+^-based solution (Figure S17), suggesting that HER is still a main competitive reaction when the applied potential is below the critical point (i.e., −0.35 V (vs. RHE) in this case). Due to the remarkably rising current densities (Figure S18), 2H-RhCu achieves the largest NH_3_ yield rate of 4936.8 mg h g^–1^_cat_ in K^+^-based electrolyte, which is nearly 3.5 times that in Na^+^-based electrolyte and 12.7 times that in Li^+^-based electrolyte (Figure 3f). Such significant yield rate increase should not originate from the ECSA change of 2H-RhCu in the electrolytes with different cations, because its ECSAs are very close to each other in Na^+^- or K^+^-containing environment, and it even displays a higher value in Li^+^-containing solution (Figures 3g and S19). The underlying mechanisms are discussed later.

Isotope labeling experiments were next implemented to confirm the origin of nitrogen in the synthesized NH_3_ in the K^+^ environment (Figures 3h and S20). The only observed characteristic peak ascribed to ^15^NH_4_^+^ in the case of ^15^NO_3_^–^ as the nitrogen resource verifies that NH_3_ does derive from the electroreduction of NO_3_^–^. The corresponding NH_3_ yield rates are similar to those using ^14^NO_3_^–^ whether the quantification was based on ultraviolet–visible (UV–vis) absorption spectroscopy or ^1^H nuclear magnetic resonance (NMR) spectroscopy methods.^8,52^ The above statement is further supported by the negligible yield of NH_3_ for the cases without potential or without NO_3_^–^. In the following, we checked the NO_3_RR performance of 2H-RhCu under lower concentrations of NO_3_^–^. The FE­(NH_3_) values are 88.8, 84.9 and 70.5% while FE­(NO_2_^–^) values are always restricted within a tiny range (2.8–4.0%) when the initial NO_3_^–^ concentrations are 50, 25, and 10 mM, respectively (Figure 3i). The corresponding NH_3_ yield rates show a gradual reduction with the decrease of NO_3_^–^ concentration. During the stability tests, 2H-RhCu exhibits sufficient catalytic selectivity and durability toward NH_3_ for 12 consecutive cycles (Figure 3j). After the long-term cycling stability test, 2H-RhCu well maintains the morphology of nanourchins, in which 2H domains are still easily observed in those building blocks (Figure S21a–c). No obvious elemental segregation of Rh and Cu can be found (Figures S21d–f and S22), and the Rh/Cu atomic ratio shows only a slight change to 78.1/21.9 (Figure S23). To highlight the merits of our proposed catalyst crystal phase and electrode/electrolyte interface dual engineering strategy, a performance comparison was made between the 2H-RhCu/K^+^ combination in this work and some other representative noble metal-dominant catalysts for the NO_3_RR reported previously (Figure 3k and Table S2). Few metal-containing catalysts can deliver comparable FE­(NH_3_) in neutral solutions at such low overpotentials.^7,27,53–62^ Especially, 2H-RhCu with a K^+^-filled OHP takes an obvious leading position on NH_3_ yield rates among Rh-dominant catalysts regardless of electrolyte pH.^14,23,24^

To further evaluate the potential of 2H-RhCu in industrial applications, we have further performed NO_3_RR measurements in a 1 M KOH + 1 M KNO_3_ electrolyte to see whether its current density can reach a higher level approaching practical demands. As can be seen in Figure S24a, 2H-RhCu cathodes can deliver a large current density of more than 500 mA cm^–2^ when the potential reaches −0.7 V (vs. RHE) in the LSV test. The current densities range from ∼75 mA cm^–2^ to ∼350 mA cm^–2^ over the potential window (Figure S24b). The FE­(NH_3_) values are 87.2, 81.9, 81.9, 85.7, and 78.6% at 0.1, 0, −0.1, −0.2, and −0.3 V (vs. RHE), respectively, while the FE­(NO_2_^–^) values remain at around 5.6–8.5% (Figure S24c). A maximum NH_3_ yield rate of 9.886 mg h^–1^ cm^–2^ was achieved at −0.3 V (vs. RHE) (Figure S24d). Such good FE­(NH_3_) and NH_3_ yield rates also endow 2H-RuCu with a certain potential for industrial electrosynthesis of NH_3_ from NO_3_^–^ in an alkaline environment.

### Mechanism Studies: Intermediate Assembly at Electrode/Electrolyte Interfaces

The aforementioned findings bring in two interesting but profound questions: (1) Why can the 2H phase of RhCu promote NH_3_ electrosynthesis in comparison with its fcc and A/C counterparts? (2) Why can K^+^ significantly enhance the selectivity and kinetics of NO_3_RR toward NH_3_ on 2H-RhCu? We carried out *in situ* electrochemical characterizations to unravel the underlying mechanisms.

In terms of the effects of crystal phase, *in situ* electron paramagnetic resonance (EPR) studies revealed that the 2H phase of RhCu has a stronger ability to favor the formation of active hydrogen radicals (*H) than the fcc one, but these *H are efficiently exhausted when converting NO_3_^–^ in both cases (Figure S25). This observation is further supported by the *H adsorption/desorption tests where 2H-RhCu has a broader *H coverage than the fcc and A/C ones (Figure S26a).^63,64^ Although more *H may be a reason for the faster NH_3_ production by individual active sites of 2H-RhCu, this variation seems to be not adequate enough to result in such an obvious discrepancy in NH_3_ selectivity.

Hence, more attention was paid to the intermediates of the NO_3_RR. According to the *in situ* differential electrochemical mass spectrometry (DEMS) patterns (Figure S27a–c), there are noticeable signal differences on several key mass-to-charge (*m*/*z*) ratios (Figure 4a). On the one hand, in contrast to A/C- and fcc-RhCu, 2H-RhCu can better stimulate *H for NO_3_^–^ hydrogenation rather than their recombination to release H_2_ (*m*/*z* = 2). On the other hand, N_2_ (*m*/*z* = 14) evolution has occurred on A/C and fcc phases but it was totally avoided on 2H one. As the critical intermediates, NO (*m*/*z* = 30) associated with HNO/NOH (*m*/*z* = 31) presented distinctly weaker signals on A/C- and fcc-RhCu in contrast to that on 2H-RhCu. The signal of NH_2_OH (*m*/*z* = 33) was detected only in the fcc case. Little undesired N_2_O (*m*/*z* = 44) parasitic product was found on these RhCu nanoalloys except for the 2H one. Furthermore, *in situ* attenuated total reflection Fourier transform infrared (ATR-FTIR) spectra revealed that fcc-RhCu starts to repulse NO_3_^–^ (1400 cm^–1^) as the NO_3_RR proceeds (Figure S28) while this phenomenon is not observed on 2H-RhCu (Figure 4b), due to the stronger electrostatic effects.^15,65^ Simultaneously, it was found that the peak intensity for adsorbed *NH_2_OH (1110 cm^–1^) is quite strong on fcc-RhCu, but the *NH_2_OH adsorption on 2H-RhCu is moderate. The excessive accumulation of *NH_2_OH on the catalyst surface would lead to the diffusion of free molecules into the electrolyte, which gives a rational explanation for why the NH_2_OH signal was only detected on the fcc sample in the DEMS tests. Combining the *in situ* DEMS and ATR-FTIR characterizations, it is reckoned that, besides better activating and stabilizing *H for subsequent hydrogenation reactions, 2H-RhCu boosts the formation and conversion of key *NO and *NH_2_OH intermediates while suppressing the recombination of N-containing intermediates to release N_2_ or other byproducts.

**Figure 4:**
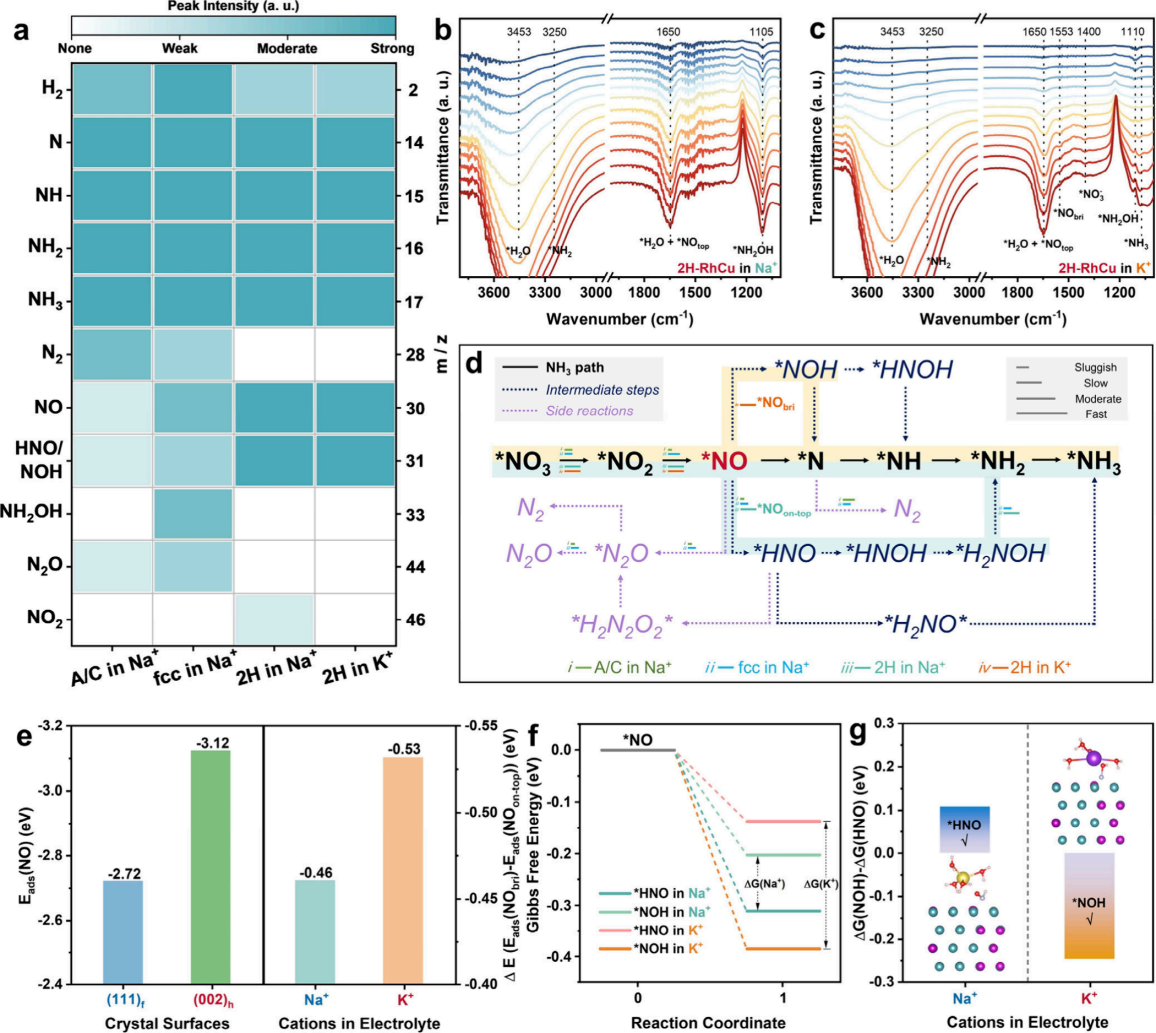
Electrochemical mechanism explorations. (a) Demonstration of various mass-to-charge (*m*/*z*) signals observed by *in situ* DEMS for A/C-, fcc-, and 2H-RhCu in Na^+^-based solutions and 2H-RhCu in K^+^-based solution. (b, c) ATR-FTIR spectra of 2H-RhCu in Na^+^ (b) and K^+^ (c) based electrolytes containing NO_3_^–^. (d) Schematic depiction of the differences in NO_3_RR pathways of different-phase RhCu catalysts in Na^+^ or K^+^ based electrolytes. (e) *E*_ads_(NO) on the (111)_f_ and (002)_h_ surfaces of RhCu crystals (left panel) and differences between *E*_ads_(NO_bri_) and *E*_ads_(NO_on‑top_) on the RhCu (002)_h_ surface in consideration of hydrated Na^+^ and K^+^ cations (right panel). (f, g) Gibbs free energy evolutions at the critical intermediate step from *NO to *HNO/*NOH (f) and differences between *G*(NOH) and *G*(HNO) with the corresponding energy-favorable atomic models as insets (g) on the RhCu (002)_h_ surface in Na^+^ and K^+^ based electrolytes.

As for the cation effects on 2H-RhCu, Na^+^ and K^+^ ions were considered for mechanism studies, given the much larger NH_3_ yield rates in the above two solutions than that in Li^+^ solution. Obviously, 2H-RhCu is still capable of efficiently promoting *H formation in K^+^ solution, and no residual *H can be recognized when conducting neutral NO_3_RR (Figure S25). However, the *H desorption in K^+^ solution shows a remarkable potential hysteresis compared to those in Na^+^ and Li^+^ solutions (Figure S26b). The potentials for complete removal of *H are 0.42 V (vs. RHE) for K^+^ and 0.38 V (vs. RHE) for both Na^+^ and Li^+^. The stronger binding between *H and the catalyst surface accounts for the more negative potentials at which the highest FE­(NH_3_) appears in the K^+^ electrolyte, to some extent.

Although the distributions of intermediate products prove almost the same for the electrolytes with Na^+^ or K^+^ by *in situ* DEMS measurements (Figures 4a and S27c,d), some variations on the adsorbed states of intermediates are found in ATR-FTIR spectra (Figure 4b,c). First, 2H-RhCu begins to have affinity toward NO_3_^–^ (1400 cm^–1^) only in K^+^-based solution when the applied potential comes into the NO_3_RR-dominant range. This uncommon phenomenon is deemed to be the consequence of reconstructed electric fields near the K^+^-filled OHP, according to some relevant previous studies on electrocatalytic CO_2_ reduction.^49–51^ Second, besides the peak at 1650 cm^–1^ ascribed to *H_2_O and *NO_on‑top_ which are both observed in both Na^+^ and K^+^ solutions, another bridge adsorption configuration of the NO intermediate (i.e., *NO_bri_) was identified at 1553 cm^–1^ only in the K^+^-containing environment.^66,67^ Third, different from the rising peak intensity of *NH_2_OH (1105 cm^–1^) in Na^+^ solution, this peak disappears in K^+^ solution while a neighbor peak corresponding to *NH_3_ gradually become recognizable, implying that the NO_3_RR does not involve *NH_2_OH as the intermediate in this case. Meanwhile, the analyses on the interfacial water structure revealed that there were no obvious differences in the composition and proportion of different interfacial water solvated configurations between Na^+^- and K^+^-based electrolytes, suggesting that the NO_3_RR performance discrepancies in the above two electrolytes are not mainly associated with water-splitting capability affected by electrostatic stabilization and hydration sphere effects (Figure S29).

The aforementioned results suggest that K^+^ plays multiple roles in facilitating the NO_3_RR toward NH_3_. On the one hand, K^+^ is able to orientate the gradient distribution of hydrated ions near the OHP, thereby establishing a surface electric field to enhance NO_3_^–^ adsorption and boost the reaction kinetics through strong electrostatic binding.^34–37^ On the other hand, compared with the Na^+^-filled OHP, the K^+^-filled one can effectively tune the intermediate assembly (e.g., *NO_on‑top_ vs. *NO_bri_) at the electrode/electrolyte interfaces and drive the NO_3_RR toward NH_3_ along those faster reaction pathways for hydrogenation. Combining these observations with previous studies, a schematic depicting the electrochemical mechanism differences caused by catalyst phase control and the cation effect is provided in Figure 4d. We deduced that one of the most possible and rational NO_3_RR pathways on 2H-RhCu with a K^+^-filled OHP should obey the sequence NO_3_^–^ → *NO_2_ → *NO → *NOH → *N → *NH → *NH_2_ → *NH_3_. The *NO_bri_ adsorbed state is more favorable toward rapid and highly selective NH_3_ electrosynthesis along the *NH_2_OH-free pathway (marked by the light-yellow shadow) while *NO_on‑top_ adsorbed state prefers the slower NO_3_RR pathway involving *NH_2_OH (marked by the light-blue shadow).

Density functional theory (DFT) simulations were further conducted to obtain theoretical insights into the mechanism differences. We first compared the adsorption energy of NO (*E*_ads_(NO)) on the mainly exposed (111)_f_ and (002)_h_ surfaces of RhCu crystals (Figure S30). Apparently, the (002)_h_ surface exhibits *E*_ads_(NO) of −3.12 eV, more negative than that (−2.72 eV) of the (111)_f_ one (Figure 4e, left panel and Table S3). Therefore, 2H-RhCu is only taken into account when the following conversion of *NO is performed after introducing cation effects (Na^+^ and K^+^) into the reaction system. To approach the real reaction conditions, hydrated Na^+^/K^+^ cation models were established here for consideration. When examining the adsorption energy of NO_on‑top_ (*E*_ads_(NO_on‑top_)) and NO_bri_ (*E*_ads_(NO_bri_)) on (002)_h_ surface, we found that there was stronger adsorption toward NO_bri_ in both cases but the energy difference Δ*E* (*E*_ads_(NO_bri_) – *E*_ads_(NO_on‑top_)) was more obvious in the model with a hydrated K^+^ cation, suggesting the easier formation of the *NO_bri_ adsorption configuration on the 2H-RhCu surface in K^+^ solution (Figure 4e, right panel and Table S3). More importantly, as observed from the Gibbs free energy barrier (Δ*G*) evolution of the critical step from *NO to *NOH/*HNO, hydrated K^+^ cations prefer to generate *NOH from *NO, while hydrated Na^+^ cations are beneficial for the conversion toward *HNO (Figures 4f and S31 and Table S4). The Δ*G* differences between *NOH and *HNO (Δ*G*(NOH) – Δ*G*(HNO)) are 0.108 eV and −0.247 eV for the hydrated Na^+^-involving solution and the hydrated K^+^-involving one, respectively (Figure 4g). It indicates that the selective conversion from *NO to *NOH in K^+^ solution is even more energy favorable in contrast to the one from *NO to *HNO in Na^+^ solution. These computational results are in good accordance with the aforementioned *in situ* electrochemical observations.

### Upgrading NO_3_RR on 2H-RhCu toward Multifunctional Energy Storage Devices

Zn-nitrate batteries are a unique device demonstration for NO_3_RR, which integrates energy supply, sewage disposal, and NH_3_ electrosynthesis.^14,57,61^ To elucidate the application potential of 2H-RhCu with a K^+^-filled OHP, we fabricated a prototype of a Zn-nitrate flow battery using 2H-RhCu as the cathode catalyst. The configuration of this flow battery is schematically illustrated in Figure 5a. The flow rates of catholyte and anolyte are both set to be 5 mL min^–1^, after initially screening the discharging voltages and stability of the cells at different flow rates (Figure S32). The general working principle can be described by eq 1 and eq 2 when discharging:

Anode:

1 $$4\mathrm{Zn}+8{\mathrm{OH}}^{-}\to 4\mathrm{Zn}O+4H_{2}O+8e^{-}$$

Cathode:

2 $${NO_{3}}^{-}+7H_{2}O+8e^{-}\to NH_{3}\cdot H_{2}O+9{\mathrm{OH}}^{-}$$

**Figure 5:**
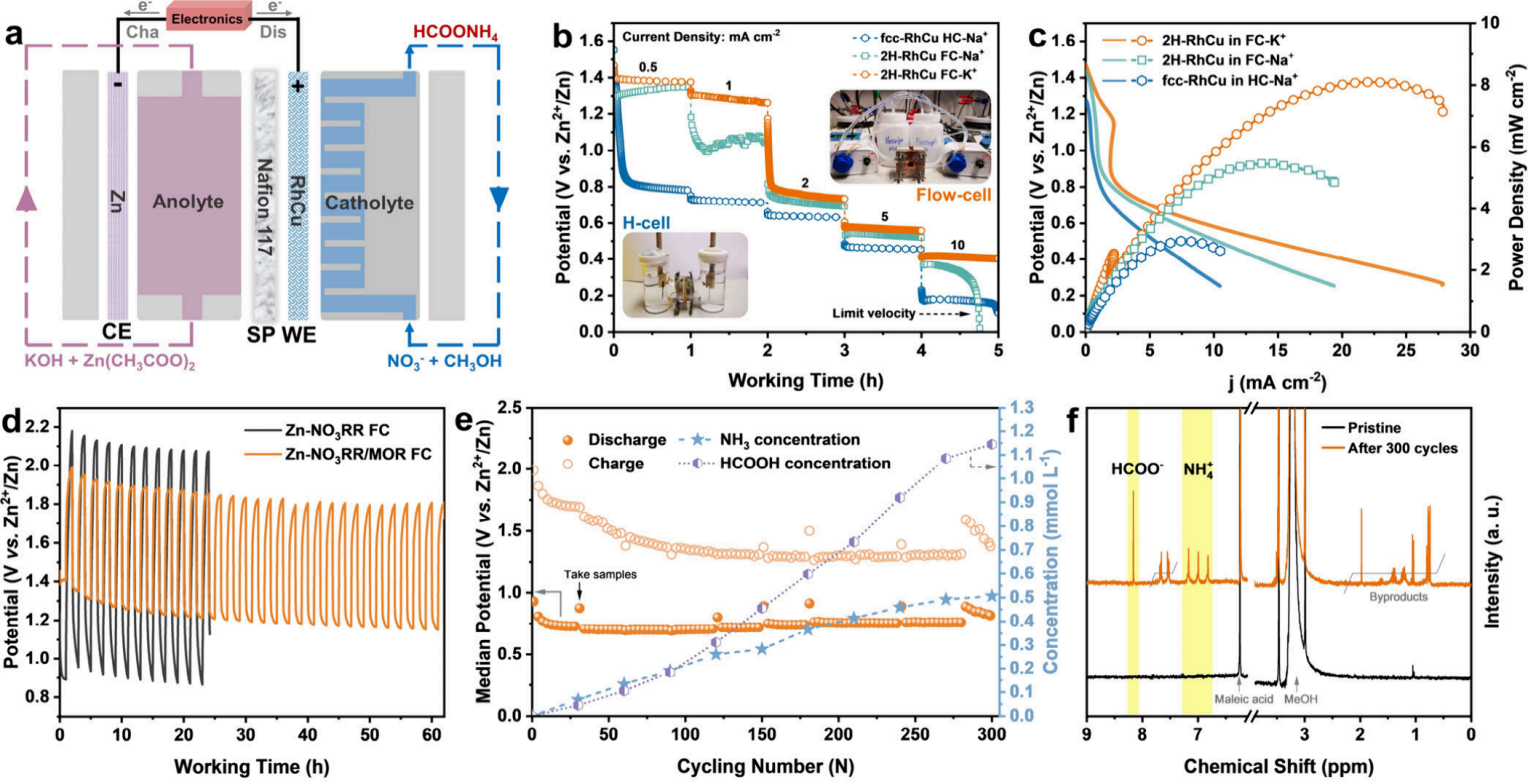
Construction of routine Zn-nitrate and rechargeable Zn-nitrate/methanol flow batteries. (a) Schematic illustration of the device configuration of as-assembled Zn-based flow batteries. (b, c) Rate performance (b) and discharging polarization profiles and resultant power density curves (c) of fcc-RhCu FC-K^+^, 2H-RhCu FC-Na^+^, and 2H-RhCu FC-K^+^. (d) Comparison of the discharge–charge profiles of Zn-NO_3_RR FC and Zn-NO_3_RR/MOR FC with 2H-RhCu at 0.1 mA cm^–2^. Discharging and charging periods are both set to be 1 h for every cycle. (e) Discharge–charge median potentials and the corresponding NH_3_ and HCOOH concentration changes in the catholyte of a Zn-NO_3_RR/MOR FC with 2H-RhCu during cycling at 0.5 mA cm^–2^. (f) ^1^H NMR spectra of the utilized cathodic electrolyte before (black line) and after (orange line) a long-term operation of 300 cycles.

In contrast to the most common H-cell (HC) configuration with traditional fcc-RhCu and Na^+^ solution (fcc-RhCu HC-Na^+^), our as-constructed flow cell (FC) has distinct advantages on rate capabilities (Figure 5b). Besides, the Zn-nitrate flow battery with 2H-RhCu and K^+^ solution (2H-RhCu FC-K^+^) can operate at a high rate of 10 mA cm^–2^ which has already exceeded the current limit by its counterpart with 2H-RhCu in a Na^+^ solution (2H-RhCu FC-Na^+^) (Figure 5b). The faster reaction kinetics enable 2H-RhCu FC-K^+^ to deliver a maximum power density of 8.10 mW cm^–2^, significantly larger than that (5.47 mW cm^–2^) by 2H-RhCu FC-Na^+^ and that (2.95 mW cm^–2^) by fcc-RhCu HC-Na^+^ (Figure 5c).

Poor rechargeability is another inevitable issue for traditional Zn-nitrate batteries relying on the oxygen evolution reaction (OER) for recharging via eq 3. To address this problem, we further introduced methanol (MeOH) into the cathodic K^+^-based neutral electrolyte of the above 2H-RhCu FC-K^+^, in light of the high electrocatalytic activity of Rh toward alcohol oxidation reactions. We first examined the electrocatalytic activity of 2H-RhCu toward the methanol oxidation reaction (MOR) in 1 M KOH + 1 M MeOH solution. Its optimal MOR mass activity is 139.3 mA mg^–1^_cat_ at 0.47 V (vs. RHE), as shown in Figure S33. The OH^–^ generated in the antecedent NO_3_RR (eq 2) offers the necessary alkaline environment to drive the subsequent MOR on Rh sites (eq 4), thereby constituting a looped discharge–charge cycle (eq 5) of rechargeable Zn-nitrate/methanol flow batteries (Zn-NO_3_RR/MOR FC).

OER Cathode:

3 $$4{\mathrm{OH}}^{-}\to {2H}_{2}O+O_{2}+4e^{-}$$

MOR Cathode:

4 $$CH_{3}\mathrm{OH}+4{\mathrm{OH}}^{-}\to HCOOH+3H_{2}O+4e^{-}$$

NO_3_RR–MOR looped reaction:

5 $${NO_{3}}^{-}+2CH_{3}\mathrm{OH}\to HCOONH_{4}+{HCOO}^{-}+H_{2}O$$

Compared with the pristine Zn-nitrate flow batteries (Zn-NO_3_RR FC) without adding MeOH, the substitution of the OER by the MOR renders the decrease of charge plateaus by ∼200 mV at 0.1 mA cm^–2^ (Figures 5d and S34). The slopes occurring at the initial stage of discharging and charging should be attributed to a certain reaction period for electrochemical equilibrium, especially at low current densities. When cycling at a higher rate of 0.5 mA cm^–2^, this Zn-NO_3_RR/MOR FC delivers a stable discharge median potential of ∼0.75 V and charge median potentials of ∼1.30 V after passivation and then steadily lasts for about 300 cycles (Figures 5e and S35). The gradual downward charge potentials within the initial 100 cycles should come from the deep activation of the MOR with the concentration increase of OH^–^ (upward pH) as cycles (Figure S36). We further investigated the NH_3_ and HCOOH concentration changes in the catholyte during cycling (Figure 5e and S37). Both of them show an escalating trend, and the corresponding slope ratio is approximately 0.42, close to the standard *n*(NH_4_^+^)/*n*(HCOO^–^) stoichiometric ratio (0.50) of the theoretical reaction along eq 5. As a result, besides some byproducts (including but not limited to formamide), a value-added chemical, ammonium formate (HCOONH_4_), is formed as the main product after the long-term usage of Zn-NO_3_RR/MOR FC (Figure 5f). The estimated yield rate of HCOONH_4_ is ∼0.16 μmol per cycle. The productivity can be further promoted by enlarging the reaction region and mass loading or working at larger discharge–charge currents. The postreaction characterizations identify that there are no obvious degradations in the morphology, phase, composition, and surface chemical state of 2H-RhCu (Figures S38–S40), even though it works as a bifunctional catalyst to repeatedly drive the electrochemical reduction–oxidation looped reactions.

## Conclusion

In summary, we have successfully synthesized three types of ultrathin RhCu nanostructures with different phases but similar morphologies and compositions by precisely regulating the wet-chemical nucleation and growth kinetics. When evaluated as NO_3_RR catalysts, 2H-RhCu demonstrates superior NH_3_ selectivity over its A/C and fcc counterparts in neutral medium. Taking 2H-RhCu as the optimal catalyst, it was found that the alkali metal cations in electrolytes also affect the NO_3_RR performance remarkably, where its selectivity and activity toward NH_3_ obey the order of K^+^ > Na^+^ > Li^+^. Especially, 2H-RhCu can deliver a high FE­(NH_3_) of 94.8% at −0.3 V (vs. RHE) and a large NH_3_ yield rate of 4936.8 mg h g^–1^_cat_ at −0.4 V (vs. RHE) in a K^+^-based electrolyte, greatly exceeding those in Na^+^/Li^+^-based ones. *In situ* electrochemical observations and theoretical simulation studies reveal that the 2H phase benefits the generation and conversion of critical *NO and *NH_2_OH intermediates while alleviating side reactions during the NO_3_RR. The K^+^-filled OHP further tunes *NO assembly from on-top to bridge adsorption configurations at electrode/electrolyte interfaces through strong electrostatic interactions, inducing a *NH_2_OH-free faster-hydrogenation pathway toward NH_3_. As a proof-of-concept application, rechargeable Zn-nitrate/methanol flow batteries using 2H-RhCu and K^+^-based electrolyte were constructed and demonstrated a low discharge–charge median potential gap of ∼0.55 V and value-added HCOONH_4_ production capability. This work not only highlights the great significance of catalyst crystal phase control and electrode/electrolyte interface engineering in facilitating multielectron transfer reactions but also offers a promising electrochemical prototype for future ecofriendly, energy-saving, and multifunctional energy storage and conversion devices.

## Supplementary Material



## Notes

Chemicals and reagents, experimental methods, characterizations, Figures S1–S40 and Tables S1–S4 (PDF)
