# Supplementary material for: Modulating the Nitrate Reduction Pathway on Unconventional Phase Ultrathin Nanoalloys for Selective Ammonia Electrosynthesis
Source: J Am Chem Soc. 2025 Jun 20;147(26):23226–38. doi: 10.1021/jacs.5c07490 (PMC12232309; doi:10.1021/jacs.5c07490)
Supplement: Supplementary file 1 [file ja5c07490_si_001.pdf]

Supporting Information

Modulating nitrate reduction pathway on unconventional phase ultrathin nanoalloys for selective ammonia electrosynthesis

Jingwen Zhou[1,2,‡], Fu Liu[1,‡], Zhihang Xu[3,‡], Jian-An Yin[2,4,‡], Liang Guo[1,2,‡], Fengkun Hao[1], Yunhao Wang[1,2], Yuecheng Xiong[1,2], Xichen Zhou[1], Cheng Wang[1], Yangbo Ma[1], Xiang Meng[1,2], Pengyi Lu[1,2], Jinwen Yin[1], An Zhang[1], Jie Wang[9], Chenliang Ye[6], Qiang Li[7], Chongyi Ling[7], Hsiao-Chien Chen[5,*], Hao Ming Chen[8], Ye Zhu[3,*], Jian Lu[2,4,*], and Zhanxi Fan[1,2,10,11,*]

[1]Department of Chemistry, City University of Hong Kong, Kowloon, Hong Kong SAR 999077, China.

[2]Hong Kong Branch of National Precious Metals Material Engineering Research Center (NPMM), City University of Hong Kong, Kowloon, Hong Kong SAR 999077, China.

[3]Department of Applied Physics, Research Institute for Smart Energy, The Hong Kong Polytechnic University, Kowloon, Hong Kong SAR 999077, China.

[4]Department of Mechanical Engineering, City University of Hong Kong, Kowloon, Hong Kong SAR 999077, China.

[5]Center for Reliability Science and Technologies, Center for Sustainability and Energy Technologies, Chang Gung University, Taoyuan 333323, Taiwan.

[6]Department of Power Engineering, North China Electric Power University, Baoding 071003, Hebei, China.

[7]Key Laboratory of Quantum Materials and Devices of Ministry of Education, School of Physics, Southeast University, Nanjing 211189, China.

[8]Department of Chemistry, National Taiwan University, Taipei 10617, Taiwan.

[9]Key Laboratory of Fluid and Power Machinery of Ministry of Education, School of Materials Science and Engineering, Xihua University, Chengdu 610039, Sichuan, China.

[10]Hong Kong Institute for Clean Energy, City University of Hong Kong, Kowloon, Hong Kong SAR 999077, China.

[11]City University of Hong Kong Shenzhen Research Institute, Shenzhen 518057, China.

‡These authors contributed equally to this work.

*Corresponding authors.

E-mail: zhanxi.fan@cityu.edu.hk; jianlu@cityu.edu.hk; yezhu@polyu.edu.hk; hc_chen@mail.cgu.edu.tw

1. Materials and Methods

1.1 Materials

Rhodium acetylacetonate ($Rh(acac)_3$, 97% metal basis), copper acetylacetonate ($Cu(acac)_2$, ACS reagent, ≥ 99.5%), oleylamine (OAm, 70%, technical grade), oleic acid (OLA, 90%, technical grade), n-hexane (anhydrous, 99.5%), methanol (MeOH, anhydrous, ≥ 99.9%) and ethanol (anhydrous, ≥ 99.9%) were purchased from Sigma-Aldrich. Formaldehyde (HCHO, ACS, 37 wt% in H_2O), lithium sulfate (Li_2SO4), sodium sulfate (Na_2SO_4), potassium sulfate (K_2SO_4), lithium nitrate ($LiNO_3$), sodium nitrate ($NaNO_3$), potassium nitrate ($K^{14}NO_3$, anhydrous, ≥ 99.0%), ^{15}N-labeled potassium nitrate ($K^{15}NO_3$, anhydrous, ≥ 99.0%), deuterium oxide (D_2O, 99.9%$_{at}$ D), dimethylsulfoxide (DMSO, anhydrous, ≥ 99.0%), sodium hydroxide (NaOH, > 98%), salicylic acid ($C_7H_6O_3$, 99%), formic acid (HCOOH, > 97%), sodium citrate ($C_6H_5Na_3O_7$, AR), sodium nitroferricyanide dehydrate ($C_5FeN_6Na_2O\cdot 2H_2O$, 99%), hydrogen peroxide (H_2O_2, 30%), maleic acid ($C_4H_4O_4$, 98%), sodium hypochlorite solution (NaClO, AR, active chlorine 6-14%$_{wt}$) and the other chemicals without special mention were purchased from Aladdin. Carbon papers (Toray TGP-H-060), Nafion solution (5%$_{wt}$ in ethanol), Nafion 117 membranes and bipolar membranes were purchased from Fuel Cell Earth. Ultrapure Milli-Q distilled water (Milli-Q System, Millipore) was used in the experiments. All the chemicals were used as received without any further purification.

1.2 Synthesis of ultrathin 2H-RhCu nanostructure

The unconventional ultrathin 2H-RhCu nanosheet assemblies were synthesized by a one-pot wet-chemical growth method. Firstly, 8 mL of $Rh(acac)_3$ solution (1 mg mL^{-1}, in OAm), 1.1 mL of $Cu(acac)_2$ solution (1 mg mL^{-1}, in OAm) and 750 μL of OLA were mixed together in a glass bottle, followed by dropwise adding 625 μL of HCHO into the mixture under stirring. After that, the obtained mixture was further stirring for 30 min to get a homogenous and

transparent solution. Next, the solution was transferred into a 30 mL autoclave and kept at 170 °C for 12 h. After cooling down naturally, the suspension was taken out from the autoclave, added with 10 mL of n-hexane and centrifuged at 10,000 rpm for 5 min to collect the sediments. The black sediments were subsequently washed with the mixture of n-hexane and ethanol at a volume ratio of 5/1 for three times. Finally, the as-obtained 2H-RhCu nanostructures were stored in n-hexane for further use.

1.3 Synthesis of ultrathin fcc-RhCu and A/C-RhCu nanostructures

In a typical synthesis of fcc-RhCu, 7.5 mL of $Rh(acac)_3$ solution (2 mg mL^{-1}, in the mixture of HCHO and distilled water at a volume ratio of 1/1) and 2 mL of $Cu(acac)_2$ solution (2 mg mL^{-1}, in the mixture of HCHO and distilled water at a volume ratio of 1/1) were mixed together in a glass bottle and stirred for 10 min. Then, the transparent solution was transferred into a 30 mL autoclave and kept at 180 °C for 12 h in an oven. The sediments were collected by centrifugation at 10,000 rpm for 5 min and washed with pure ethanol for three times. The obtained fcc-RhCu products were stored in ethanol for further use. As for the synthesis of A/C-RhCu, the reaction temperature was set to be 80 °C and the holding time was extended to 36 h, while keeping all the other synthetic parameters unchanged.

1.4 Preparation of working electrodes

The synthesized RhCu electrocatalysts with different phases were directly mixed with Nafion (5 $\%_{wt}$ in ethanol) at an alloy/binder weight ratio of 5/1 without any carbon additives. A certain amount of n-isopropanol was introduced into the above mixture to prepare the catalyst ink with an alloy concentration of ~ 5 μg μL^{-1} through sonication. The obtained catalyst ink was slowly dropped onto the carbon paper plates (CP, 1 × 1 cm^2, Toray H-060) at room temperature. The mass loadings of alloys were controlled at 0.2 ~ 0.3 mg cm^{-2}. After dried in

vacuum for over 24 h, these cathodes ascribed to various RhCu nanocatalysts were obtained and stored under vacuum for further use.

1.5 Electrochemical measurements

Before electrochemical measurements, Nafion 117 membrane was immersed in 5 $\%_{wt}$ H_2O_2 at 80 ℃ for 1 h and then in distilled water at 80 ℃ for another 1 h for activation and purification. All the NO_3RR performances were evaluated in H-cells separated by activated Nafion 117 membranes at room temperature. The CP-supported catalysts, Ag/AgCl electrode, and platinum (Pt) plate (1 × 1 cm^2) were used as the working electrode, reference electrode and counter electrode, respectively. A mixed solution consisting of 0.5 M M_2SO_4 and 0.1 M MNO_3 (M = Li, Na and K) was used as both the cathodic and anodic electrolytes, and it was saturated with Ar for at least 10 min every time before measurements. Note that all the working electrodes were subjected to a continuous cyclic voltammetry (CV) scanning for activation and surface stabilization over the potential window of 0.3 ~ -0.7 V (*vs.* RHE) for 60 cycles in 0.5 M M_2SO_4 solution in the absence of NO_3^-. LSV measurements were conducted on a three-electrode system at a scan rate of 5 mV s^{-1} in the electrolyte with or without NO_3^-, unless specified. Chronoamperometry (CA) tests were performed at a series of different applied potentials with 85% IR compensation in a typical H-cell with 25 mL of electrolyte in both anodic and cathodic sides under a stirring rate of 600 rpm to accelerate mass transfer. Note that IR compensation is cancelled when conducting NO_3RR in the 1 M KOH + 1 M KNO_3 electrolyte due to the maximum cell voltage (10 V) limited by the testing equipment. To obtain ECSA, CV measurements were performed at different sweep rates within the corresponding non-Faradaic intervals of different cathodes and a linear relation was demonstrated by fitting the function between current density differences (ΔJ, J_{anodic}-$J_{cathodic}$) at the midpoint of interval and scan rates. The slope of the linear relation was C_{dl}. Then ECSA could be obtained through the following equation: $ECSA = (C_{dl}/C_s) \times S_E$,

where C_s and S_E represent the specific capacitance (0.04 mF cm^{-2}) and the apparent area of electrode (1 cm^2), respectively. All the aforementioned electrochemical measurements were conducted on an Ivium electrochemical workstation (Ivium-n-Stat, Netherlands). All potentials were referred to RHE in this work unless specification, according to the following formula: $E(RHE) = E(Ag/AgCl) + 0.197 + 0.059 \times pH$. All the MOR performances were evaluated using the similar cathodes for NO_3RR tests. The CP-supported catalysts, Ag/AgCl electrode, and platinum (Pt) plate (1 × 1 cm^2) were used as the working electrode, reference electrode and counter electrode, respectively, in a single chamber cell with 30 mL of electrolyte. Before measurements, all the working electrodes were activated through CV sweeping over the potential window of 0.05 to 1.1 V (*vs.* RHE) for 60 cycles in Ar-saturated 1 M KOH. After that, MOR performances were evaluated in the mixture of 1 M KOH and 1 M MeOH over the potential window of 0.05 to 1.1 V (*vs.* RHE). CA tests were conducted at 0.74 V (*vs.* RHE) for 3600 s under stirring at 600 rpm.

In-situ differential electrochemical mass spectrometry (DEMS) measurement was performed on a Linglu DEMS analysis system, where the carbon paper electrode coated with different RhCu electrocatalysts, Pt wire, and saturated Ag/AgCl electrode were used as the working electrode, counter electrode, and reference electrode, respectively, in a specially designed classical cell. Four periods of cathodic LSV from 0.2 to -0.7 V (vs. RHE) at 5 mV s^{-1} were implemented to drive NO_3RR in neutral media. There was a rest interval between adjacent LSV processes until the detected signals recovered to the initial state. *In-situ* attenuated total reflection Fourier transform infrared spectroscopy (ATR-FTIR) test was conducted using a Nicolet iS50 FTIR spectrometer equipped with a mercury cadmium telluride (MCT) detector cooled with liquid nitrogen. The gold-deposited Si prism covered with RhCu catalyst, Pt wire, and saturated Ag/AgCl electrode were applied as the working electrode, counter electrode, and reference electrode, respectively. All spectra after background correction are shown in

the transmittance mode. Cathodic LSVs from 0.3 to -0.5 V (vs. RHE) at 2 mV s^{-1} were performed to drive the NO_3RR in different neutral electrolytes without prior CV activation for working electrodes. Electron paramagnetic resonance (EPR) analysis was carried out on a Bruker EMX Plus spectrometer operating at a frequency of around 9.6 GHz, sweep width of 100 G and power of 3 mW. A molecular trap, 5,5-dimethyl-1-pyrroline N-oxide (DMPO), was used to capture the active hydrogen radicals to generate the DMPO-H adduct that can be detected by EPR. In a typical measurement, a potentiostatic electrolysis at -0.3 V (vs. RHE) was performed on the targeted RhCu cathode in an H-type cell under the protection of Ar. After electrolysis for 30 s, the EPR signals were examined. Every fresh RhCu cathode was firstly tested in the NO_3^--free neutral solution with the assigned cation ions (i.e., Na^+ or K^+) and then re-tested in the solution containing NO_3^- under the same conditions.

1.6 Determination of ammonia and nitrite

To determine the amount of NH_3 produced after NO_3RR for 1 h at various conditions, indophenol blue method was used. In detail, 1 mL of electrolyte was taken out after test and diluted with distilled water at suitable folds. Firstly, 2.5 mL of solution A (composed of 0.625 M NaOH, 0.36 M salicylic acid and 0.17 M sodium citrate) were added. Then 300 μL of solution B (sodium nitroferricyanide,10 mg mL^{-1}) and 150 μL of solution C (NaClO, active chlorine 6-14 $\%_{wt}$) were added, successively. It was then subject to adequate vortex for homogeneous mixing and then kept without disturbance for 3 h under ambient environment. Next, UV-vis spectrophotometry was used to examine the absorbance values at 660 nm of these mixed solutions, and the NH_3 concentrations could be obtained according to the standard curves. The amount of generated NH_3 was also calculated by ^1H NMR method. 1 mL of electrolyte after NO_3RR was added with 10 μL of $C_4H_4O_4$ which acted as the quantitative reference. After that, 50 μL of 4 M H_2SO_4 were further introduced to provide a weak acid environment. Subsequently, 450 μL of the above mixed solution were added with

50 μL of D_2O for NMR tests. The integral peak area ratios between NH_4^+ and $C_4H_4O_4$ were calculated and the corresponding NH_4^+ concentrations could be determined according to the standard curve. The standard NH_4^+ solutions with given concentrations of $(NH_4)_2SO_4$ in 0.05 M H_2SO_4 were prepared to establish the calibration curves for UV-vis and ^{1}H NMR methods. As for the ^{15}N-labeling experiments, all the electrochemical operations and quantitative analysis were the same except for using $K^{15}NO_3$ as the nitrogen resource.

To determine the amount of NO_2^-, 1 mL of electrolyte was taken out after test and diluted with distilled water at suitable folds. Then 10 μL of N-(1-Naphthyl) ethylenediamine dihydrochloride solution (10 mg mL^{-1}) were dropped into the diluted electrolyte. After 20 min, these solutions were measured by UV-vis spectrophotometry. The absorbance values at 540 nm were recorded to calculate the concentrations of NO_2^- according to the standard curve. The standard calibration curve could also be acquired by using the standard solutions with given NO_2^- concentrations.

Faradaic efficiencies (FEs) and weight-normalized yield rates of NH_3 and NO_2^- were calculated by the following equations:

$$FE\ (NH_3) = (8 \times F \times M \times V \times D_n)/\ Q$$

$$NH_3\ yield\ rate = (17 \times M \times V \times D_n)/\ (t \times W)$$

$$FE(NO_2^-) = (2 \times F \times M \times V \times D_n)/\ Q$$

$$NO_2^-\ yield\ rate = (46 \times M \times V \times D_n)/\ (t \times W)$$

, where F is the Faraday constant (96485 C mol^{-1}), M is the measured concentration of NH_3 or NO_2^-, V is the volume of used electrolyte (0.025 L), Q is the total amount of charge transfer during NO_3RR, D_n is the dilution factor, t is the reaction time, and W is the mass loading of working electrodes.

The energy efficiency for NH_3 production is defined as the ratio of chemical energy to applied electrical power, which was calculated by the following equation:

$$EE_{NH_3} = \frac{(E^0_{OER} - E^0_{NH_3}) \times FE_{NH_3}}{E_{OER} - E_{NH_3}}$$

where E^o_{OER} represents the equilibrium potential of water oxidation (1.23 V *vs.* RHE), E^0_{NH3} (0.28 V (*vs.* RHE) under neutral condition) is the equilibrium potential of nitrate electroreduction to NH_3, FE(NH_3) is the Faradaic efficiency of NH_3, E_{OER} is the applied potential for oxygen evolution reaction (here assuming the overpotential for water oxidation is 0, i.e., E_{OER} is 1.23 V *vs.* RHE), E_{NH3} is the applied potential for NH_3 production.

1.7 Determination of formic acid (HCOOH)

The amount of HCOOH produced during the cycling of Zn-nitrate/methanol flow batteries was calculated by ^{1}H NMR methods. Firstly, 1.5 mL of the cathodic electrolyte were taken from the battery after given cycles and diluted at suitable folds using distilled water. After that, 600 μL of the diluted solution were added with 30 μL of DMSO/D_2O mixture in which DMSO acted as the quantitative reference for NMR measurements. The integral area ratios between HCOOH and DMSO peaks were calculated and the corresponding HCOOH concentrations could be calculated based on the calibration curve. The HCOOH solutions with given concentrations were prepared ahead to obtain the standard NMR spectra and then establish the calibration curve accordingly.

1.8 Assembly of primary Zn-nitrate and rechargeable Zn-nitrate/methanol flow batteries

The CP-supported catalysts (active area: 1 × 1 cm^2; catalyst mass loading: ~ 1 mg) were used as the cathodes. Polished Zn plates (active area: 1 × 1 cm^2) worked as both reference and counter electrodes (anodes). An activated Nafion 117 membrane was used as the separator to

isolate the cathodic and anodic electrolytes in a typical flow cell. When constructing rechargeable Zn-nitrate/methanol flow batteries, 100 mL of 1 M KOH aqueous solution with 0.02 M $Zn(CH_3COO)_2$ as additive were introduced into the anolyte tank while another 100 mL of 0.5 M K_2SO_4 + 0.1 M KNO_3 solution added with 1 M MeOH were added into the catholyte tank. The flow rates of anolyte and catholyte were both fixed at 5 mL min^{-1} after performance optimization. The assembly of routine primary Zn-nitrate flow batteries was similar to that of the above rechargeable Zn-nitrate/methanol flow batteries but using the mixed solution without MeOH as the cathodic electrolyte. The galvanostatic discharge/charge measurements of these Zn-based hybridized flow batteries were carried out using LAND battery test systems (CT2001A, Wuhan LAND Co. LTD) under different conditions at room temperature. Discharge polarization profiles were obtained by conducting cathodic LSV from OCV to 0.25 V (*vs.* Zn^{2+}/Zn) and then the resultant power density curves could be figured out.

1.9 DFT calculations

To obtain suitable 2H and fcc structures of RhCu solid solution alloys synthesized in this work for simulation studies, six kinds of Cu-doped Rh models for every phase were randomly generated at the Rh/Cu atomic ratio of 3/1. After relaxation, structure with the most favourable energy was picked out as the optimal crystal model. All the computational simulations were performed on basis of DFT calculations with plane-wave technique which is implemented in the Vienna ab initio simulation package (VASP) [1]. Gradient corrected exchange-correlation functional of Perdew, Burke, and Ernzerhof (PBE) models was used under the projector augmented wave (PAW) method [2], with a cut-off kinetic energy of 400 eV for plane wave basis. The convergence criterion of the total energy was set up to be within 1×10^{-4} eV, while all the atoms and geometries were optimized until the residual forces became less than 2×10^{-2} eV/Å. For all the calculations regarding the adsorption of atoms/molecules on RhCu $(111)_f$ and $(002)_h$ surfaces, they were carried out within a K-point

3×3×1 grid. In the vertical direction, a vacuum layer with thickness of 15 Å was introduced for all the surfaces. The adsorption energy of various intermediates was defined as $E_{ads} = E_T - (E_A + E_S)$, where E_A and E_S represent the energy of the specified atoms/molecules and the pristine system, respectively, and E_T is the total energy of the corresponding system after the specified atoms/molecules were adsorbed on the crystal surface.

1.10 Characterizations

XRD measurements were conducted on a Rigaku SmartLab SE X-ray diffractometer using Cu K_α radiation at a wavelength of 1.5406 Å. SEM and EDS data were acquired by a Thermo Fisher Scientific (TFS) Quattro S scanning electron microscope operated at 15 kV. TEM images and EELS results were obtained using a field emission JEM-2100F (JEOL, Japan) operated at 200 kV. The HAADF-STEM images and EDS elemental mappings were obtained on a double aberration-corrected Spectra 300 TEM/STEM (TFS, USA) operated at 300 kV and equipped with a Super-X EDS spectrometer (TFS, USA). XPS analysis was based on a ESCALAB 220i-XL electron spectrometer from VG Scientific using 300 W Al KR radiation (base pressure < 10^{-5} mbar). The UV-Vis adsorption spectra were obtained on a classical UV-vis spectrophotometer (Shimadzu 1700). The further quantitative analysis of NH_4^+ and HCOOH in electrolyte was carried out using the nuclear magnetic resonance spectroscopy (NMR 300MHz, Bruker AVANCE ||| BBO Probe). The XAS data were collected in the total fluorescence mode, which was performed at beamline 12B of Taiwan beamline at Spring-8 of National Synchrotron Radiation Research Center (NSRRC). The electron storage ring was operated at 8.0 GeV with constant current of about 400 mA. The incident beam energy was monochromatized by the Si (111) double crystal monochromator[3]. The XAS-related data processing was performed by using Athena and Artemis software packages[4].

2. Supporting Figures

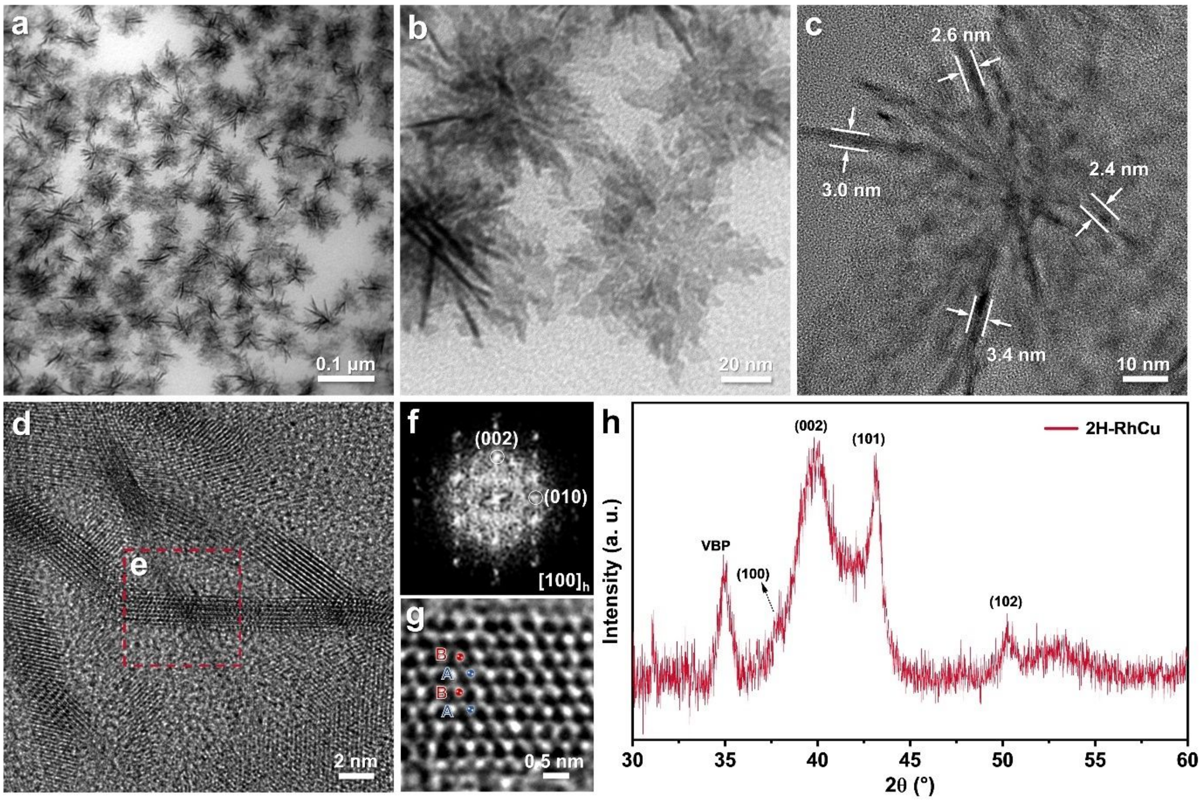

Figure S1. Microstructural characterization of ultrathin 2H-RhCu nanosheet assemblies. (a-c) TEM images of as-synthesized 2H-RhCu nanostructures showing their morphology (a, b) and nanosheet thickness (c). (d-g) Typical HRTEM image, corresponding FFT pattern and zoom-in HRTEM image of 2H-RhCu nanosheets along the $[100]_h$ direction. (h) XRD pattern of the synthesized 2H-RhCu nanostructures. VBP, vacated Barlow packing.

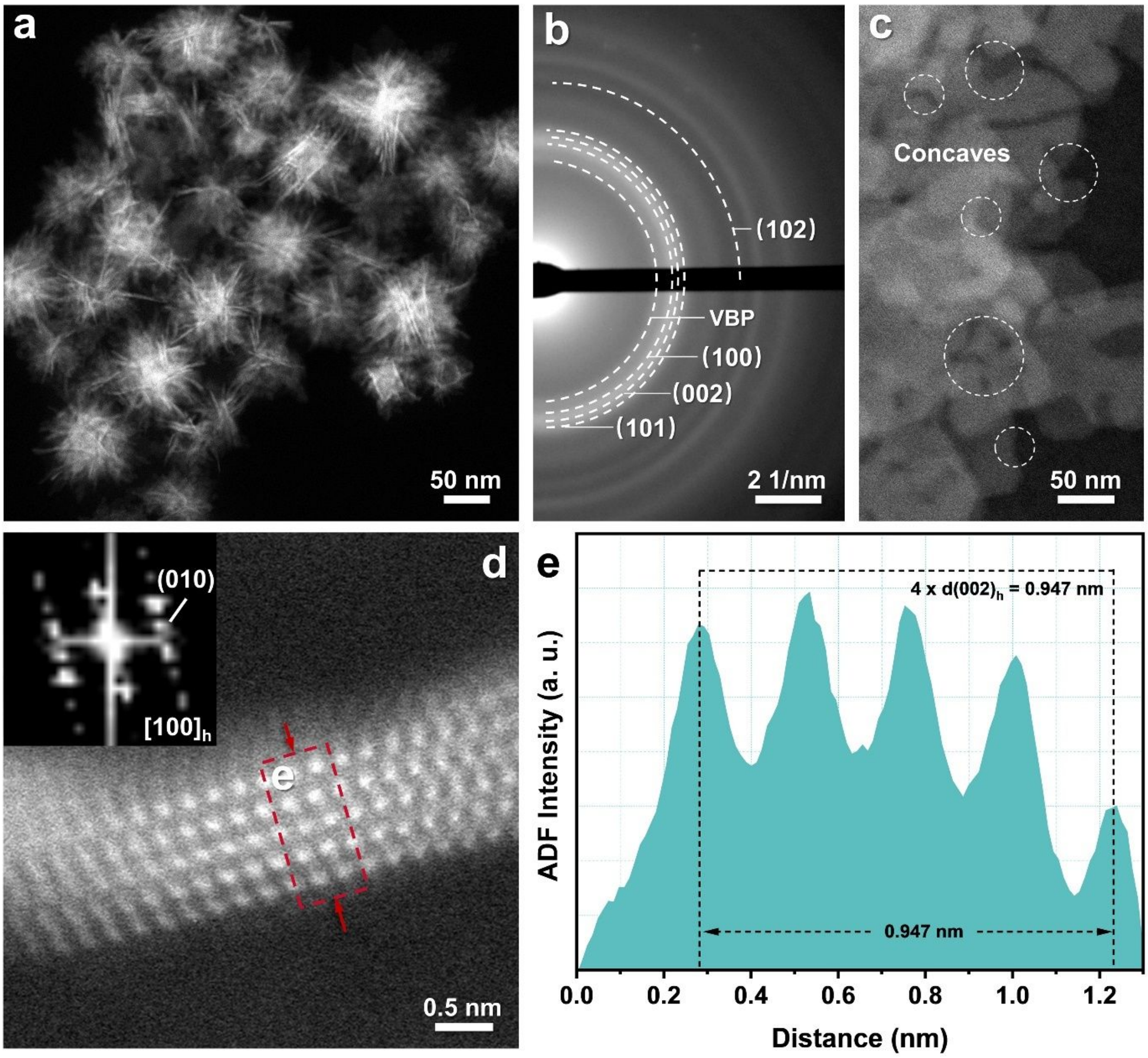

Figure S2. Structural features of ultrathin 2H-RhCu nanostructures. (a,b) Low-magnification HAADF-STEM image showing the “nano-urchin” morphology (a) and the corresponding SAED pattern (b) of synthesized 2H-RhCu. (c) HAADF-STEM image near the edge of a 2H-RhCu nano-urchin. (d) Atomic-resolution HAADF-STEM image of a single nanosheet unit constituting the 2H-RhCu nanostructures along the $[100]_h$ direction. The corresponding FFT pattern is shown as inset. (e) Integrated pixel intensity profile along the arrow ascribed to the region marked by the red dashed square in (d).

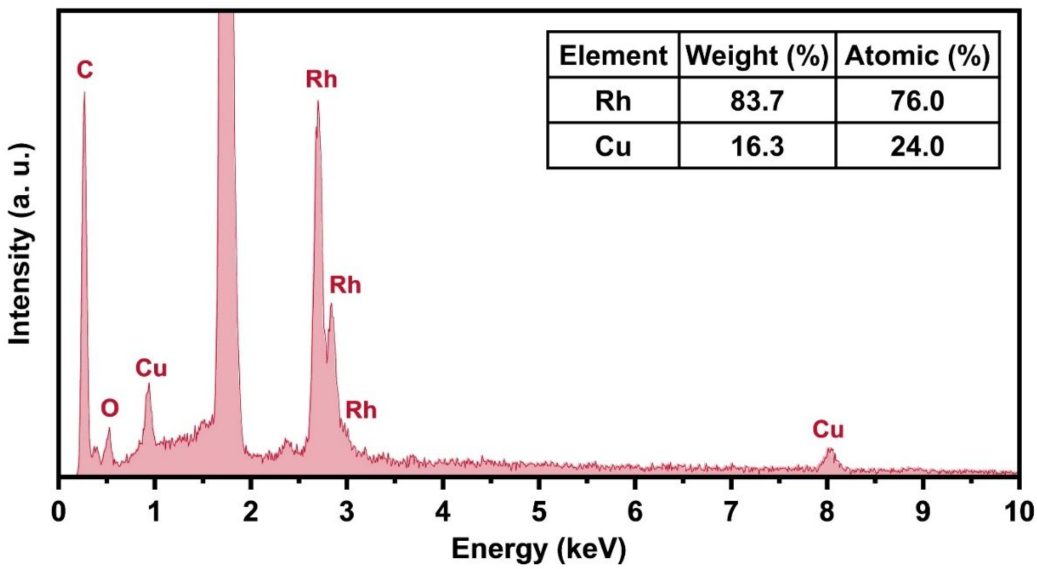

Element	Weight (%)	Atomic (%)
Rh	83.7	76.0
Cu	16.3	24.0

Figure S3. EDS spectrum of 2H-RhCu nanostructures. Inset: a table showing the elemental ratio between Rh and Cu.

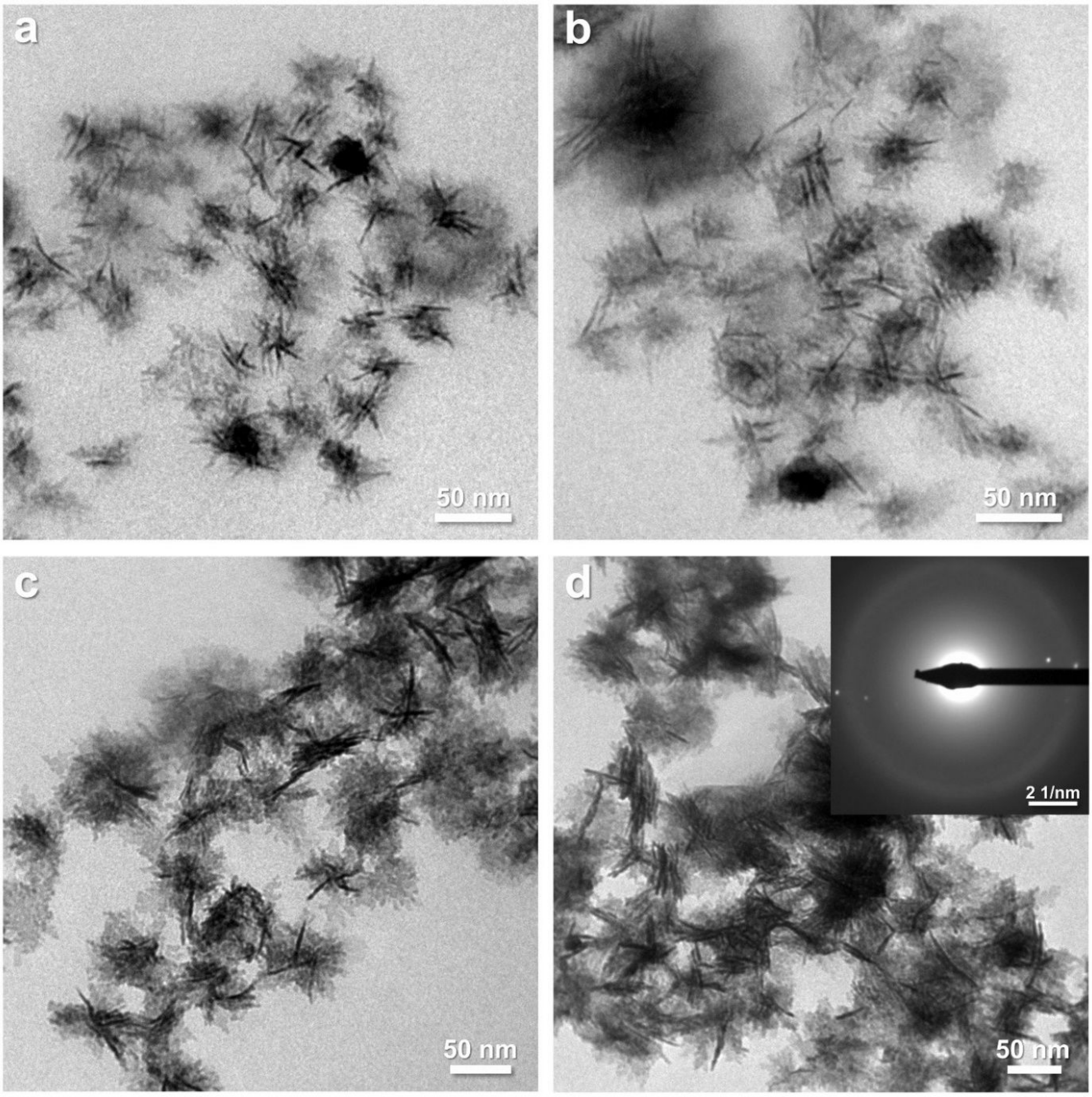

Figure S4. Time-dependent TEM observation of 2H-RhCu growth in one-pot chemical synthesis. (a-d) Typical TEM images of 2H-RhCu growth in OAM+OLA+HCHO solution at the reaction time of 6 h (a,b) and 9 h (c,d). Inset of (d): the corresponding SAED pattern.

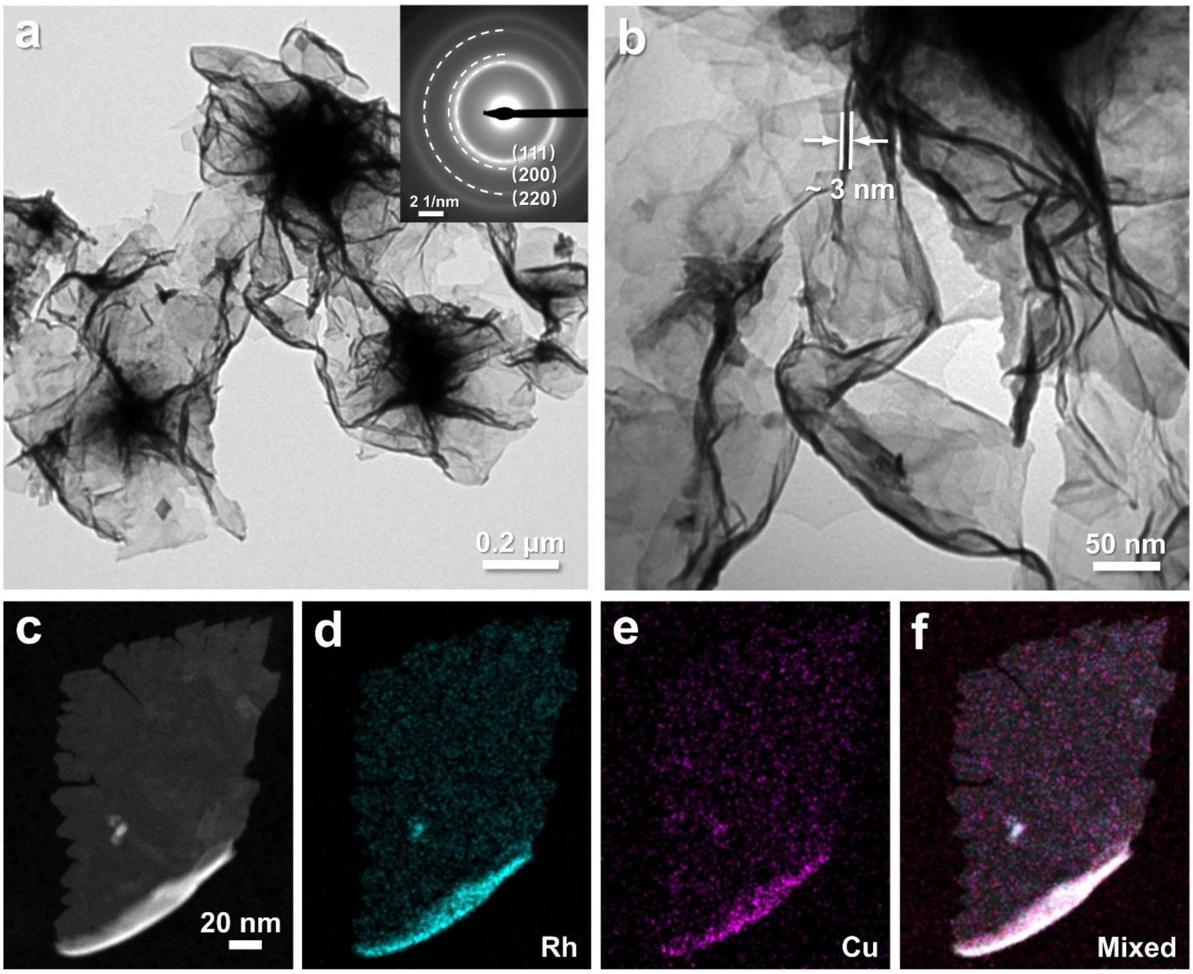

Figure S5. Structural and elemental characterizations of fcc-RhCu nanosheet assemblies. (a,b) low-magnification (a) and zoom-in (b) TEM images of fcc-RhCu. Inset of (a): the corresponding SAED pattern. (c-f) HAADF-STEM image (c) and corresponding EDS elemental maps (d-f) for fcc-RhCu.

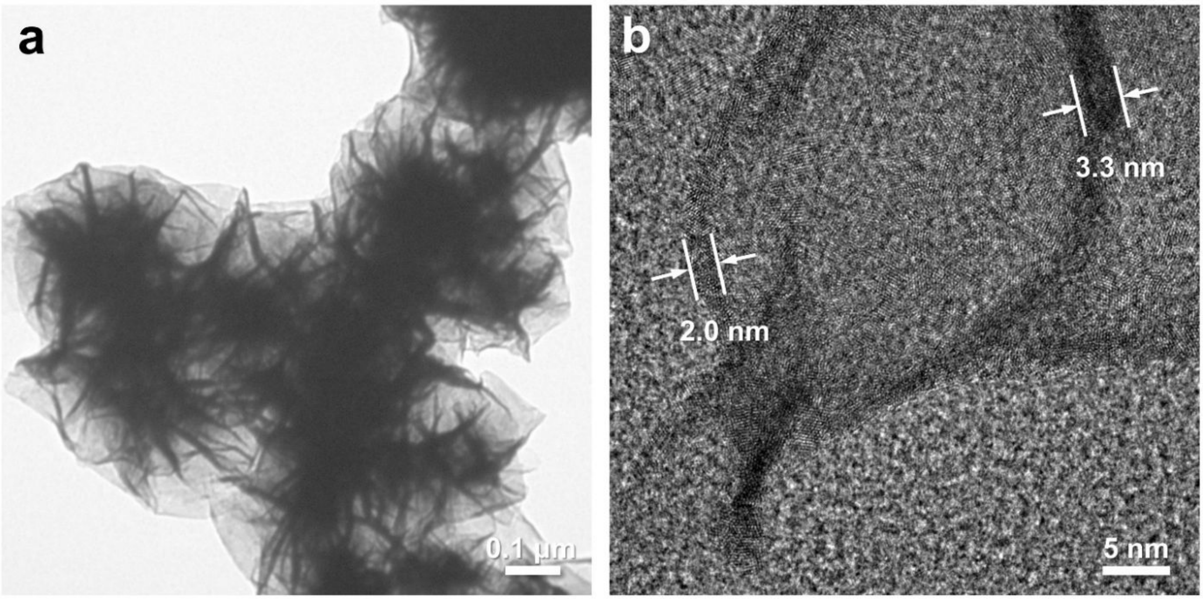

Figure S6. Structural characterization of A/C-RhCu nanosheet assemblies. (a,b) Low-magnification TEM (a) and HRTEM (b) images of A/C-RhCu.

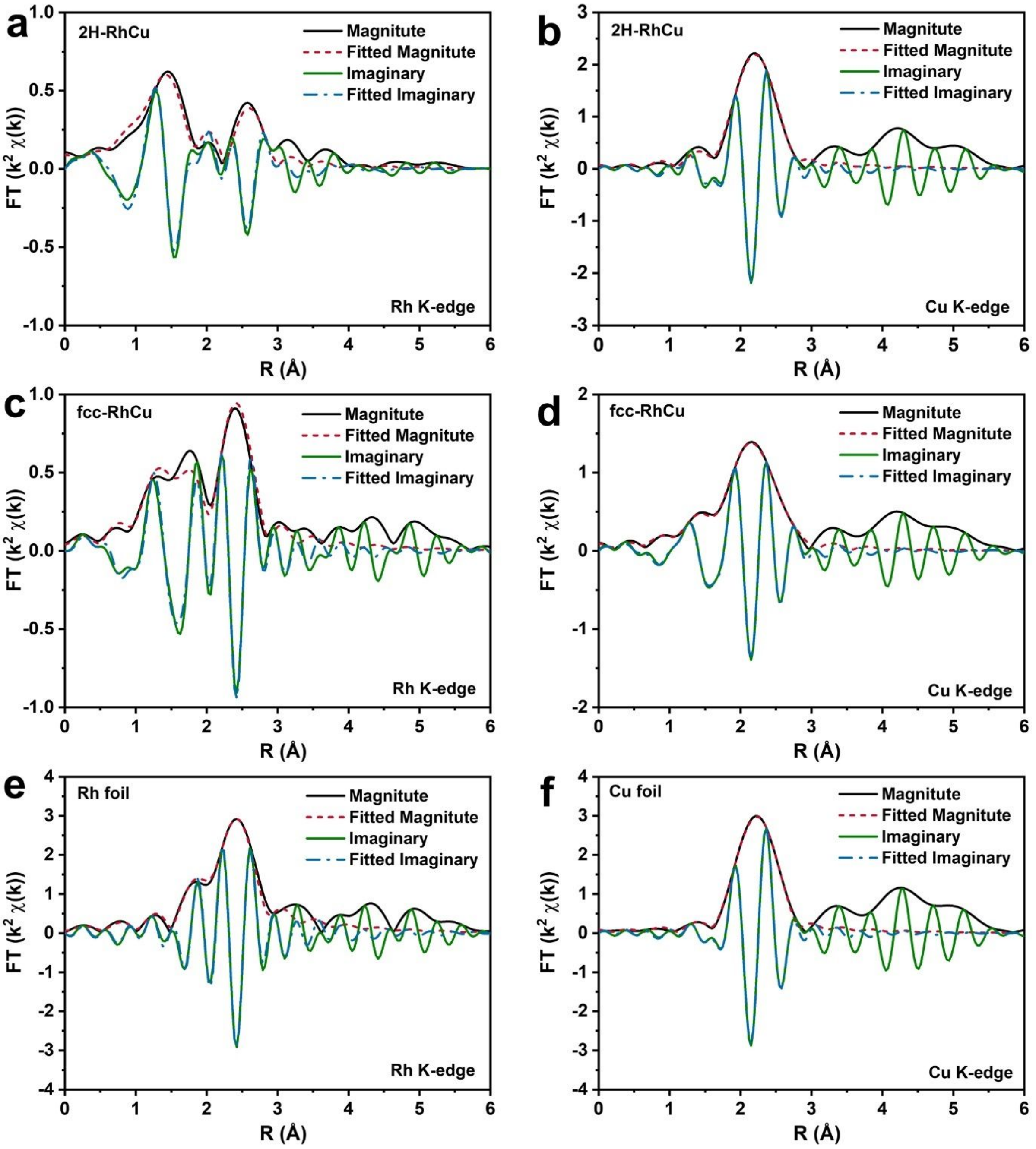

Figure S7. Fitting results of the EXAFS spectra. (a-f) The k^2-weighted R space fitting results at Ru K-edge (a, c, e) for 2H-RhCu (a), fcc-RhCu (c), Rh foil (e) and the fitting results at Cu K-edge (b, d, f) for 2H-RhCu (b), fcc-RhCu (d), Cu foil (f).

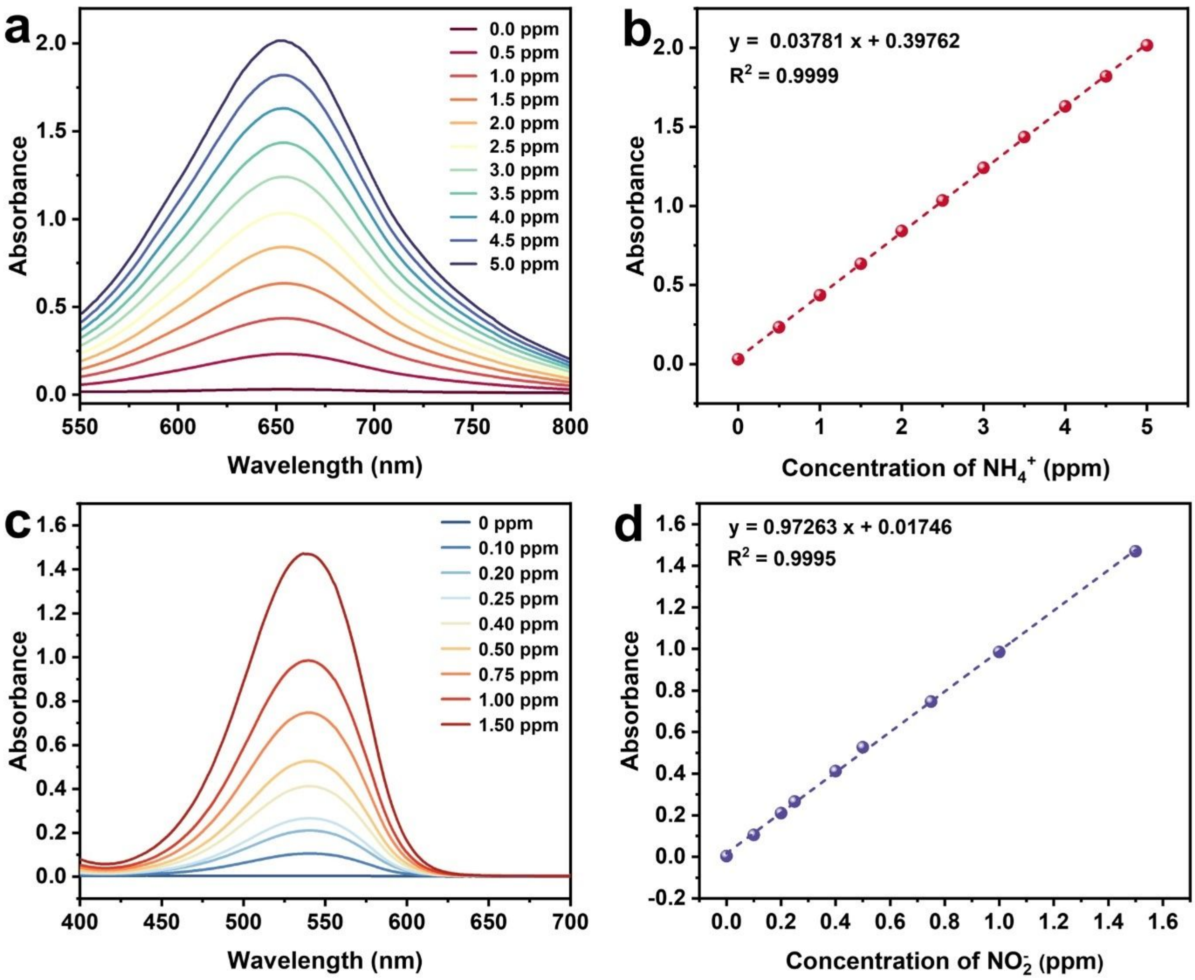

Figure S8. Establishment of the calibration curves of NH_4^+ and NO_2^- concentrations by colorimetric methods. (a,b) UV-vis absorption curves over the wavelength range of 800 - 550 nm (a) and calibration curve (b) of the solutions with given NH_4^+ concentrations. (c,d) UV-vis absorption curves over the wavelength range of 700 - 400 nm (c) and calibration curve (d) of the solutions with given NO_2^- concentrations.

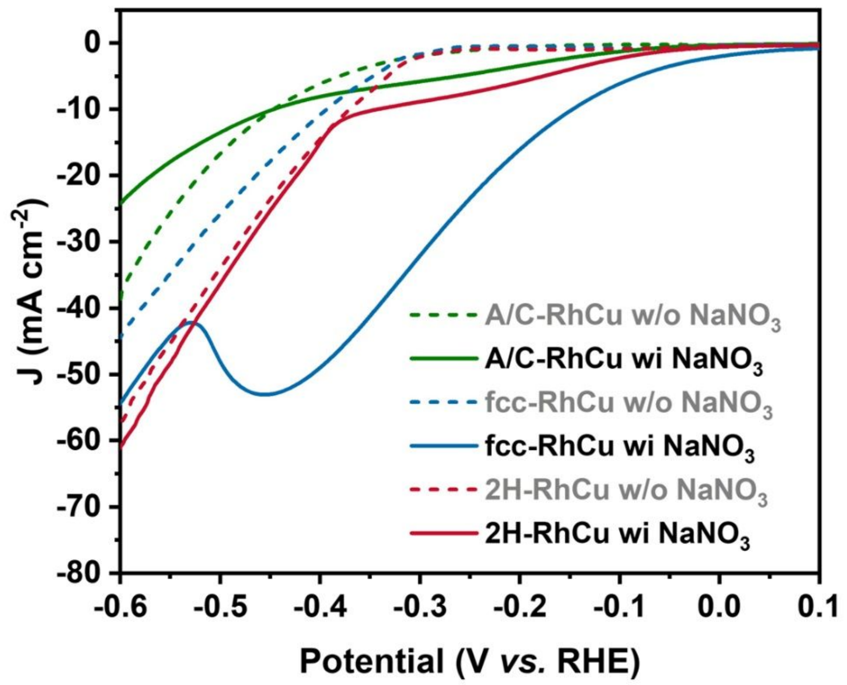

Figure S9. LSV curves of RhCu nanocatalysts with different phases. The solid and dash lines represent the ones by A/C-, fcc- and 2H-RhCu cathodes recorded in the presence or absence of 0.1 M $NaNO_3$ in 0.5 M Na_2SO_4 solutions, respectively. The linear scanning is conducted at 5 mV s^{-1} over the potential window of 0.1 ~ -0.6 V (*vs.* RHE).

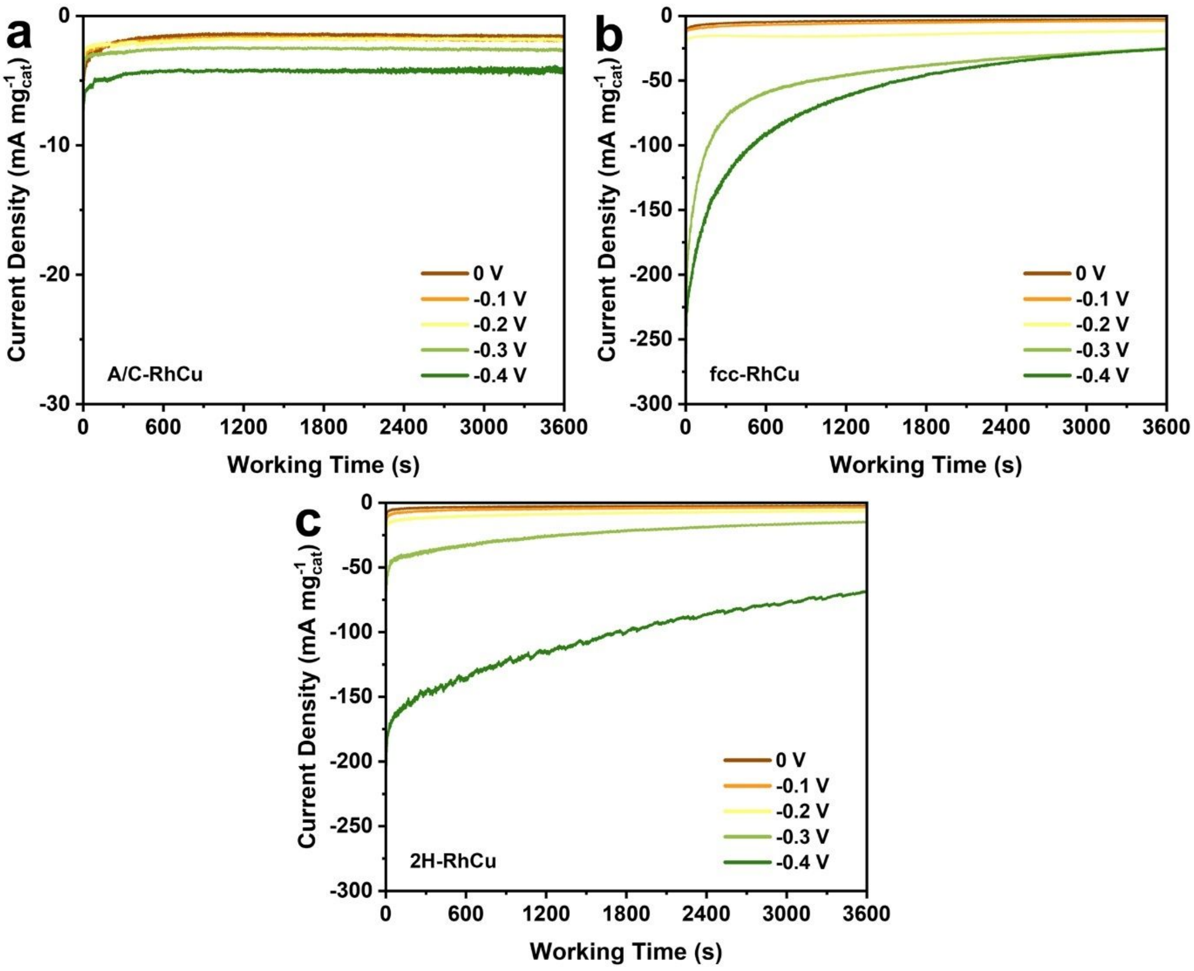

Figure S10. CA curves of RhCu nanocatalysts with different phases in Na^{+}-based neutral electrolyte. (a-c) CA curves at different applied potentials for A/C- (a), fcc- (b) and 2H-RhCu (c) cathodes in 0.5 M Na_2SO_4 solution added with 0.1 M $NaNO_3$.

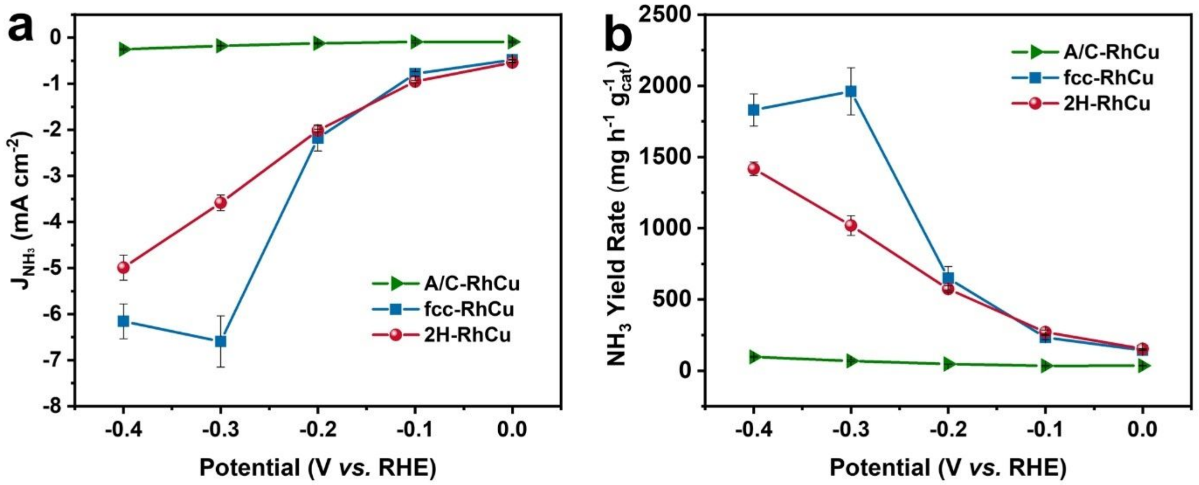

Figure S11. NO_3RR performance towards NH_3 in Na^+-based neutral electrolyte. (a,b) Partial current densities towards NH_3 (a) and NH_3 yield rates (b) at different applied potentials for A/C-, fcc-and 2H-RhCu cathodes in 0.5 M Na_2SO_4 solution containing 0.1 M $NaNO_3$.

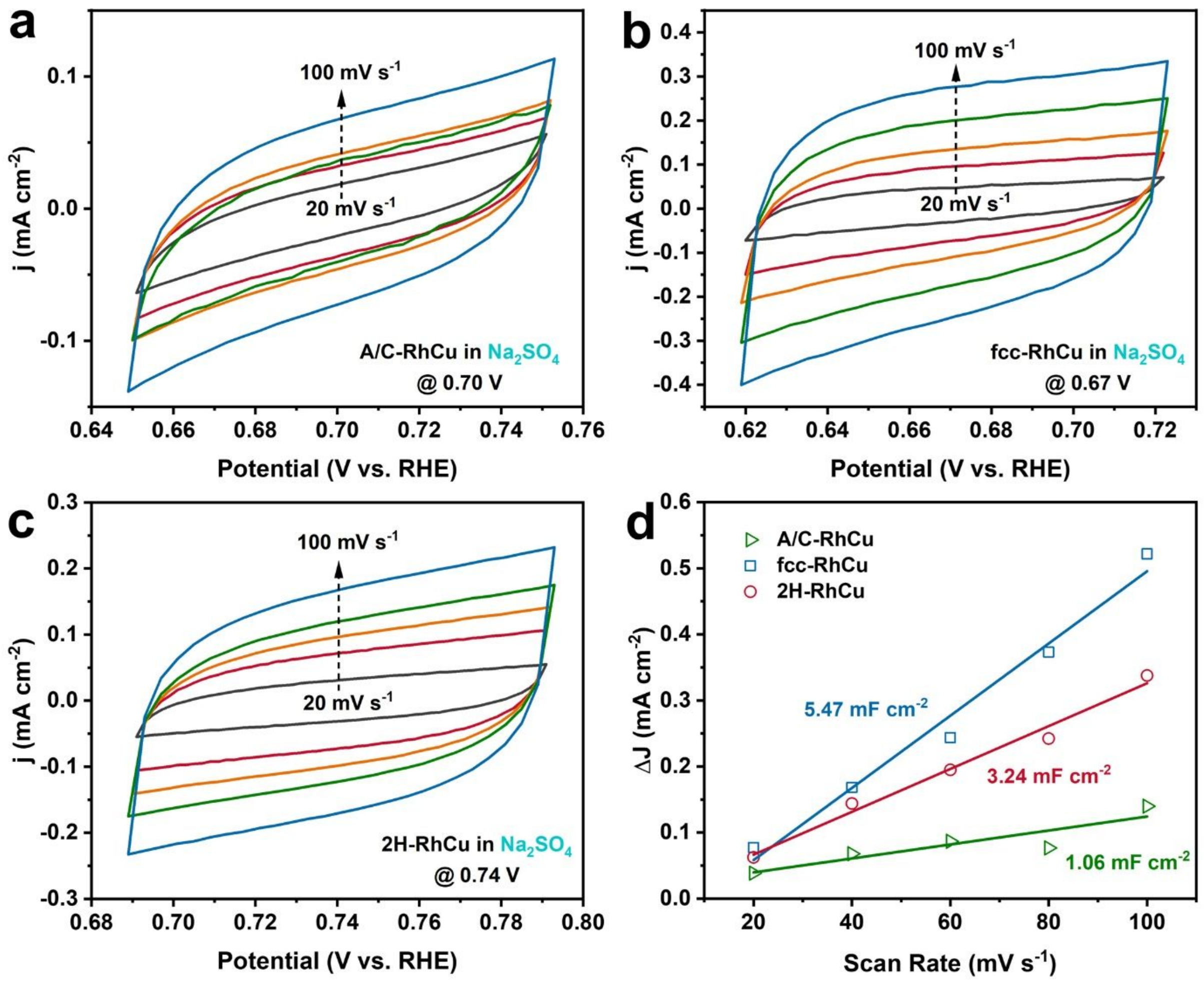

Figure S12. ECSA measurements on RhCu nanocatalysts with different phases. (a-c) Cyclic voltammetry profiles within the non-Faradaic ranges around the open-circuit potentials of A/C- (a), fcc- (b) and 2H-RhCu (c) cathodes at the scan rates of 20, 40, 60, 80 and 100 mV s^{-1} in Na^+-based electrolyte. (d) Fitting results of C_{dl} for A/C-, fcc- and 2H-RhCu cathodes.

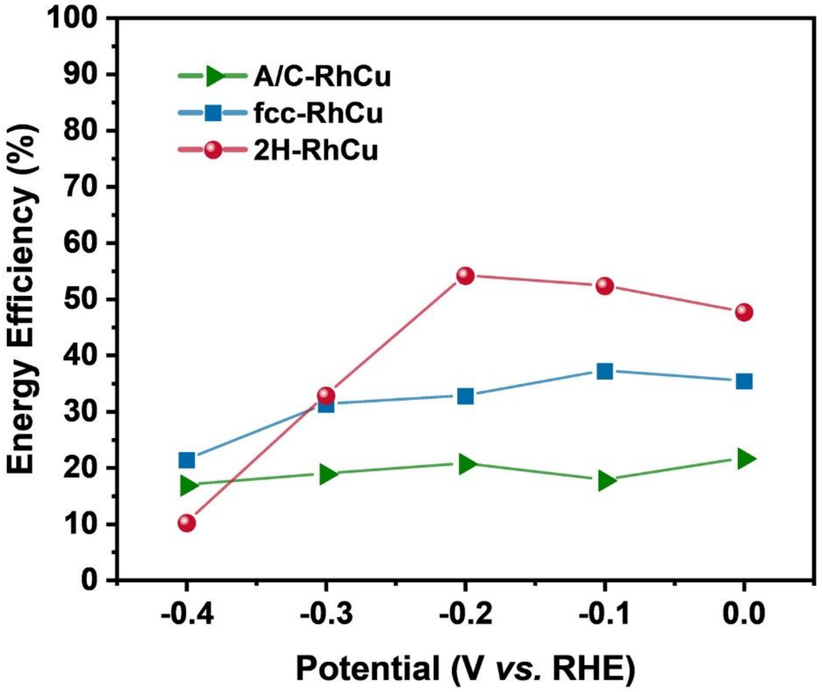

Figure S13. The energy efficiencies of A/C-, fcc- and 2H-RhCu cathodes in the 1 M Na_2SO_4 + 0.01 M $NaNO_3$ electrolyte.

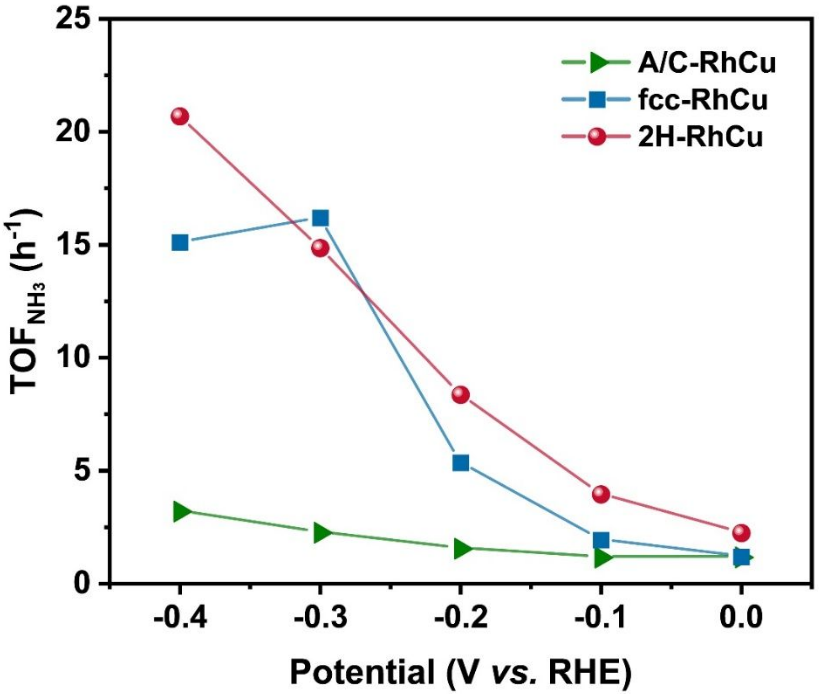

Figure S14. TOFs of NH_3 for A/C-, fcc- and 2H-RhCu cathodes in 1 M Na_2SO_4 + 0.1 M $NaNO_3$ electrolyte at different applied potentials.

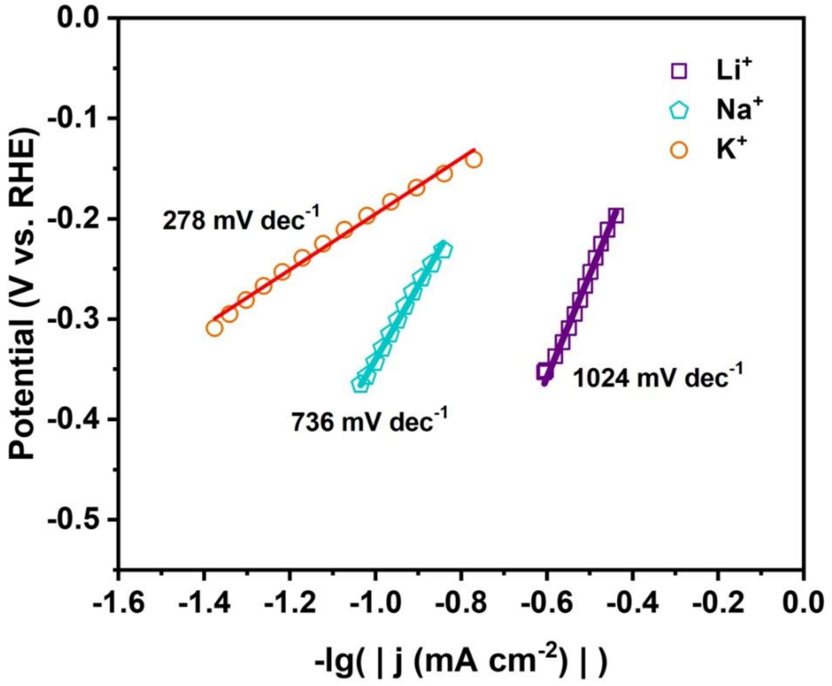

Figure S15. Tafel slopes of 2H-RhCu cathodes during NO_3RR in electrolytes with different cation ions. The Tafel slopes were obtained by converting the data of LSV curves in the potential range of stage S1 marked in Figure 3d.

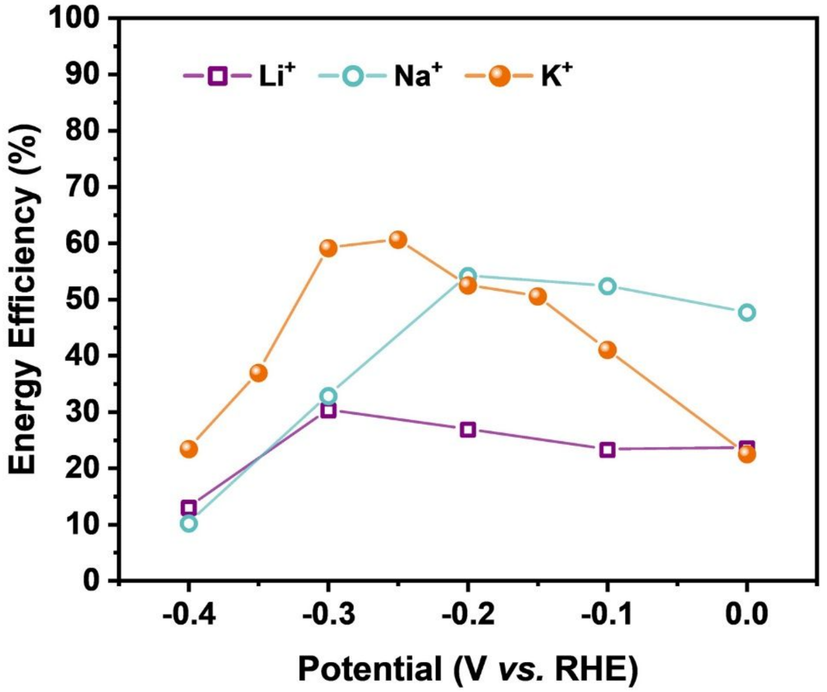

Figure S16. Energy efficiencies of 2H-RhCu cathodes in the Li^+, Na^+ and K^+-based electrolytes.

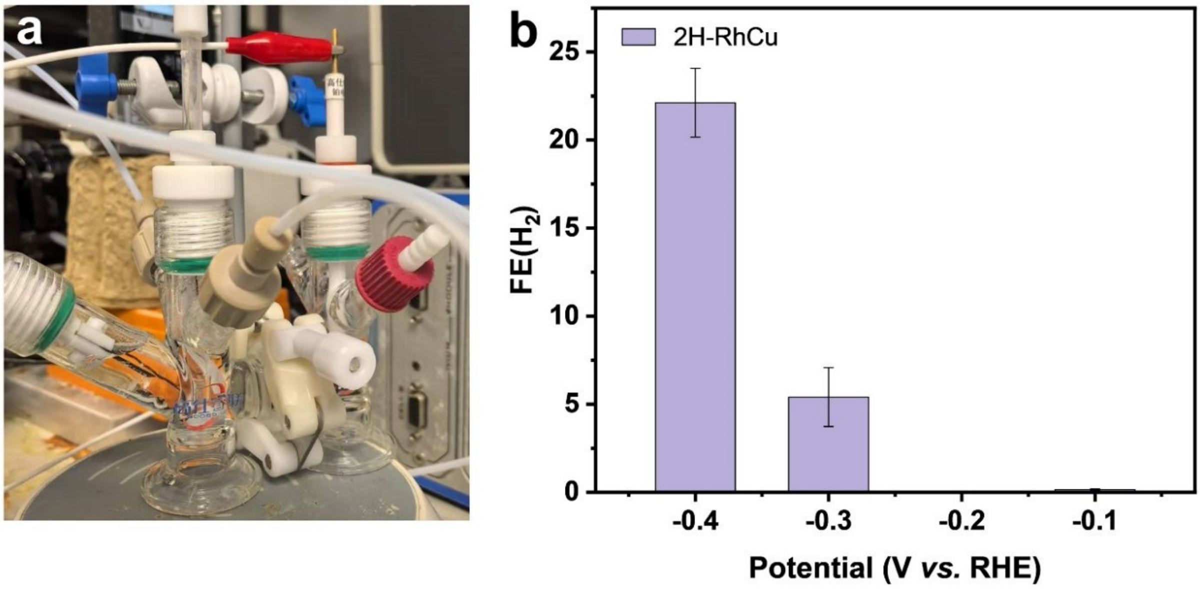

Figure S17. H_2 quantitation in NO_3RR on 2H-RhCu cathodes in the K^+-based electrolyte. (a) Digital photograph depicting the utilized H-cell for gaseous H_2 quantitation. (b) Faradaic efficiencies towards H_2 of 2H-RhCu during NO_3RR at -0.1, -0.3 and -0.4 V (*vs.* RHE) in the 1 M K_2SO_4 + 0.1 M KNO_3 neutral electrolyte.

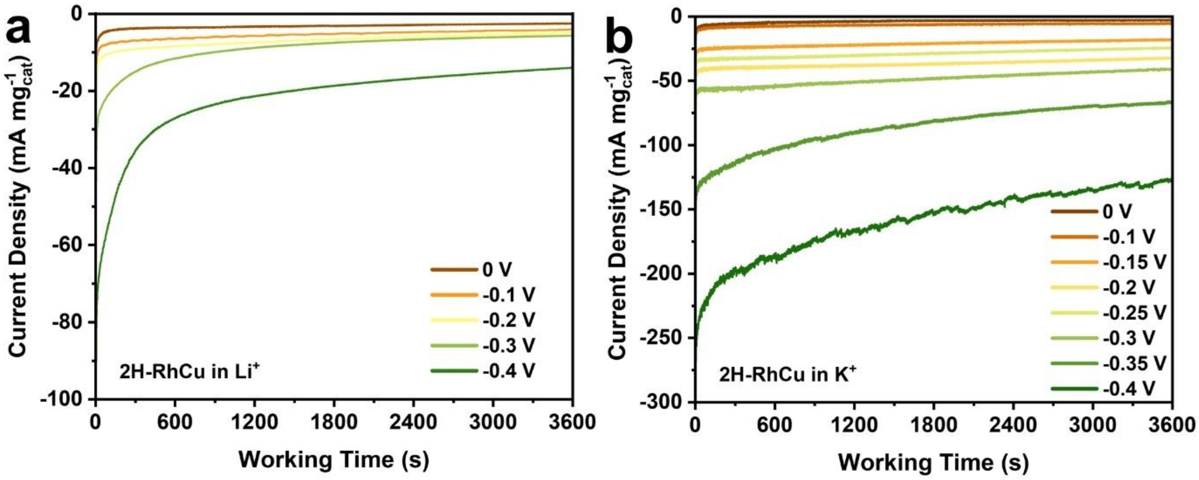

Figure S18. CA curves of 2H-RhCu in Li^+ and K^+ based neutral electrolytes. (a,b) CA curves at various applied potentials for 2H-RhCu cathodes in 0.5 M Li_2SO_4 solution containing 0.1 M $LiNO_3$ (a) and in 0.5 M K_2SO_4 solution containing 0.1 M KNO_3 (b).

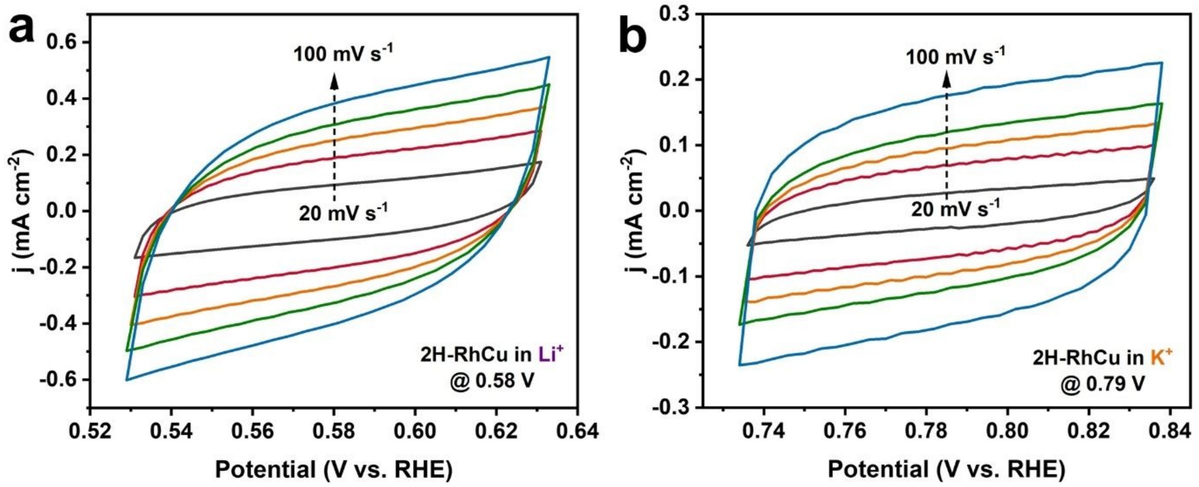

Figure S19. ECSA measurements on 2H-RhCu in neutral media with different cations. (a, b) Cyclic voltammetry profiles within the non-Faradaic ranges around the open-circuit potentials of 2H-RhCu cathodes at the scan rates of 20, 40, 60, 80 and 100 mV s^{-1} in Li^+-based electrolyte (a) and in K^+-based electrolyte (b).

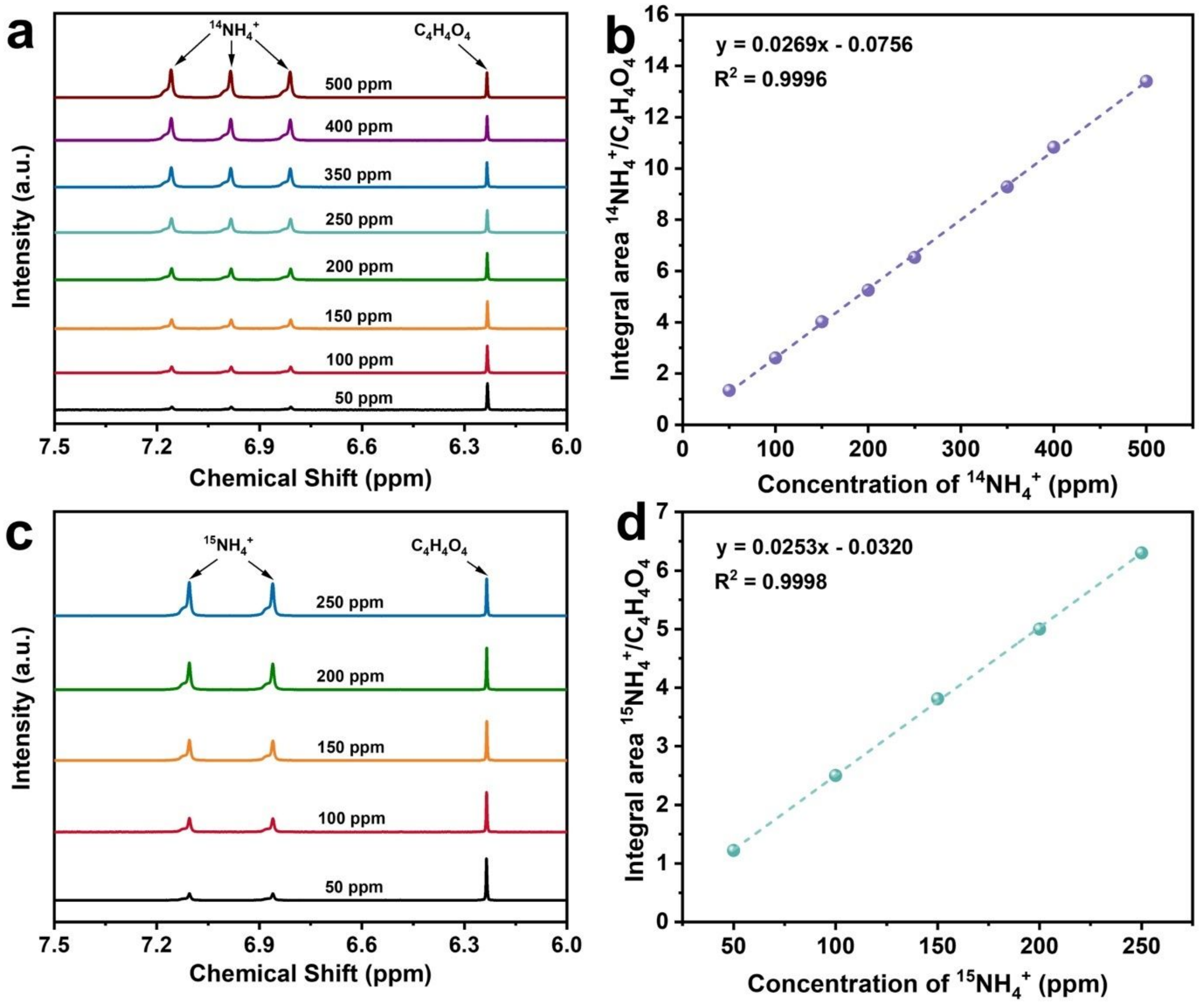

Figure S20. Establishment of the calibration curves of $^{14}NH_4^+$ and $^{15}NH_4^+$ concentrations by ^1H NMR methods. (a,b) ^1H NMR spectra for solutions with $^{14}NH_4^+$ concentrations of 50, 100, 150, 200, 250, 350, 400 and 500 ppm (a) and calibration curve (b) of the solutions with given $^{14}NH_4^+$ concentrations. (c,d) ^1H NMR spectra for solutions with $^{15}NH_4^+$ concentrations of 50, 100, 150, 200, and 250 ppm (c) and calibration curve (d) of the solutions with given $^{15}NH_4^+$ concentrations.

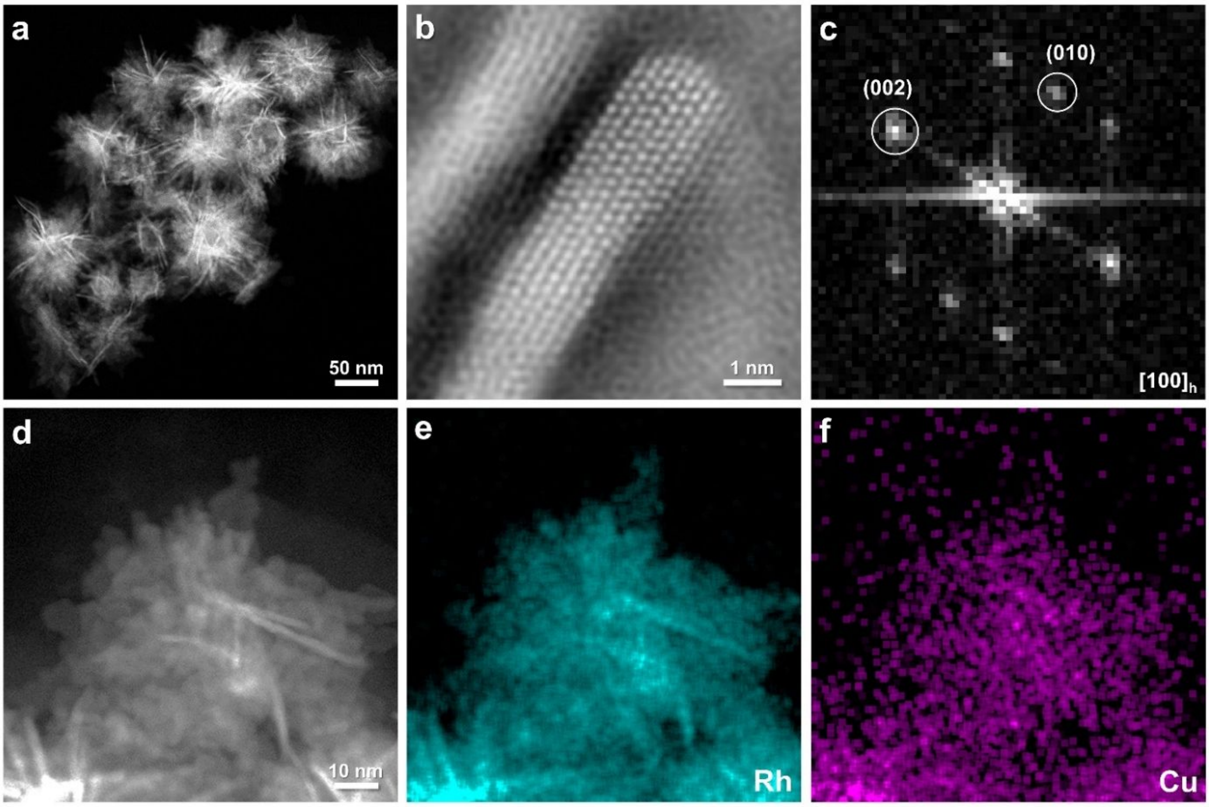

Figure S21. Structural and composition characterizations of 2H-RhCu after the long-term cycling stability test. (a-c) Low-magnification (a) and atomic-resolution (b) HAADF-STEM images of 2H-RhCu after the long-term test, and the FFT pattern (c) corresponding to (b). (d-f) HAADF-STEM image (d) and elemental mapping of Rh (e) and Cu (f) of a 2H-RhCu nano-urchin after 12 cycles of NO_3RR tests.

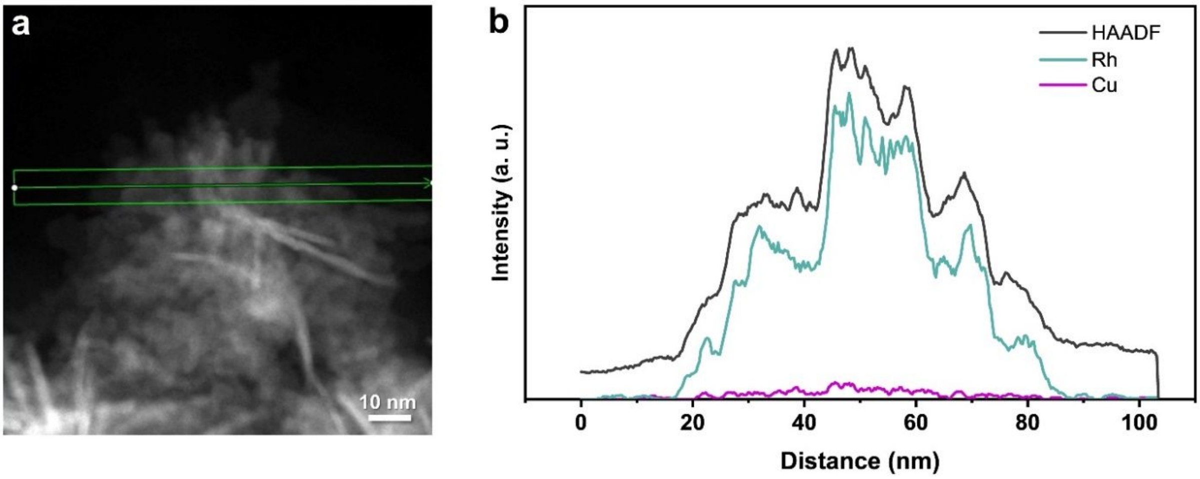

Figure S22. Composition analysis of 2H-RhCu after the long-term test. (a,b) HAADF-STEM image (a) and EDS line scan profiles (b) of an individual 2H-RhCu nano-urchin after NO_3RR test.

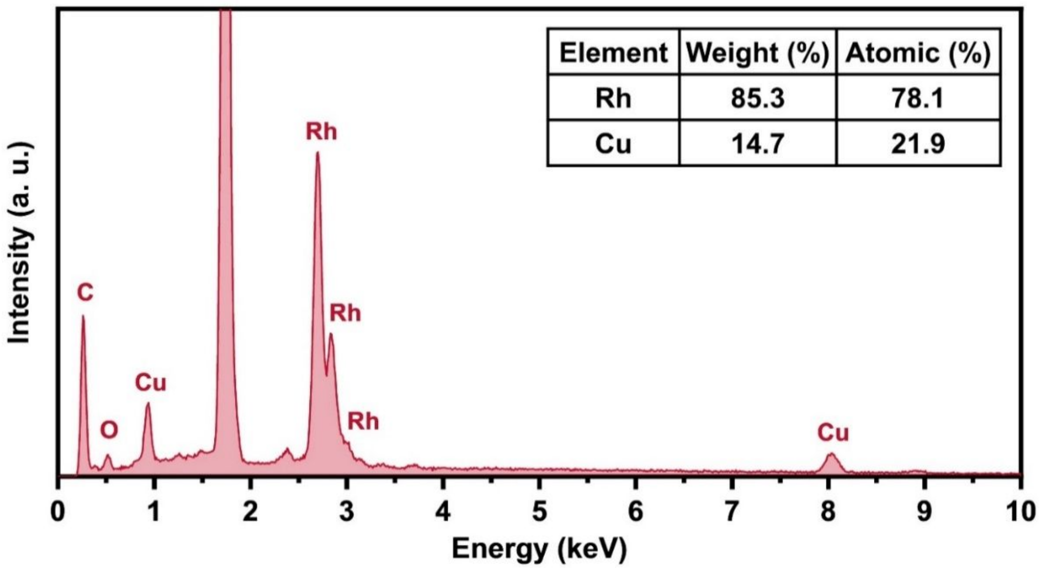

Figure S23. EDS spectrum of 2H-RhCu cathodes after the cycling stability test of NO_3RR. Inset: a table showing the elemental ratio between Rh and Cu.

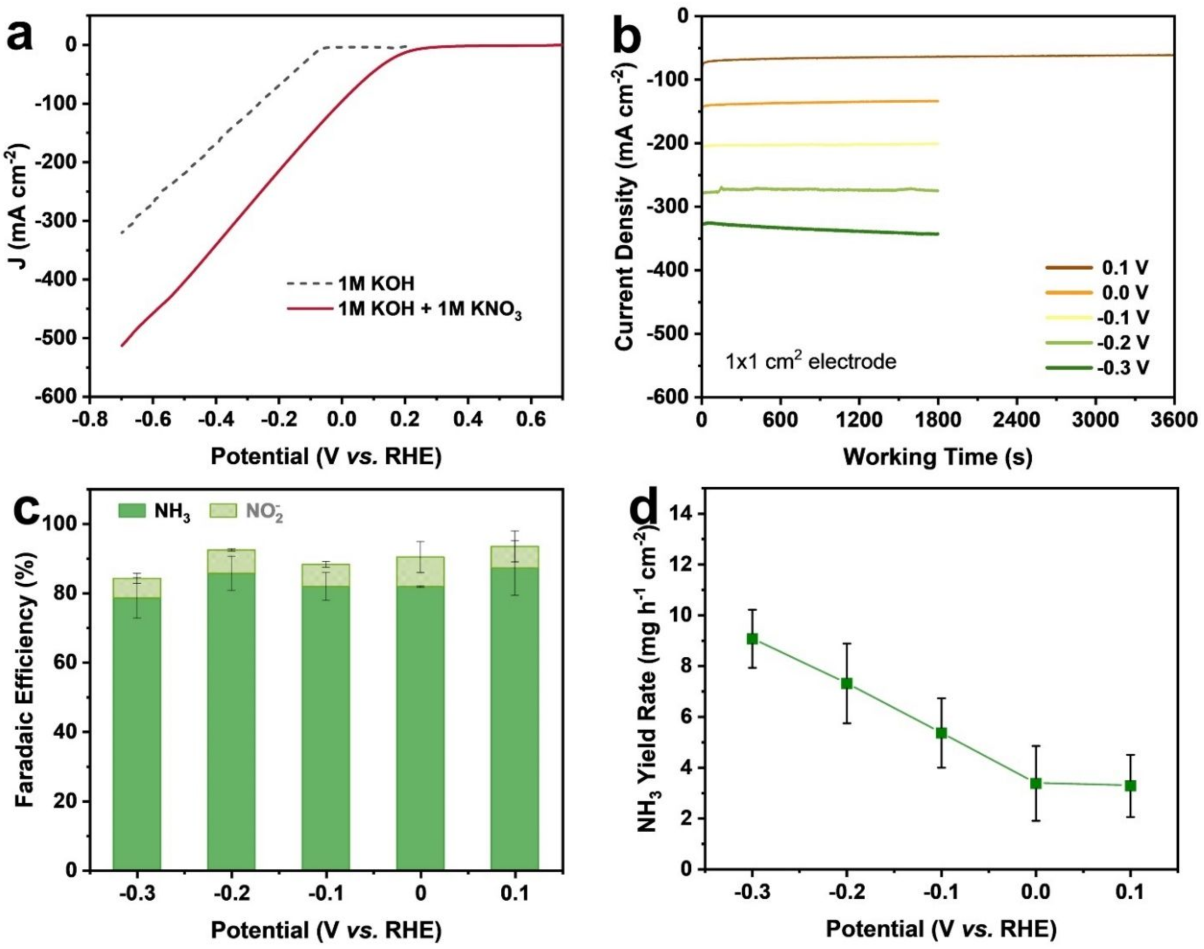

Figure S24. NO_3RR performance of 2H-RhCu in alkaline electrolyte. (a) LSV curves at 5 mV s^{-1} of 2H-RhCu cathodes in 1 M KOH solution added with and without 1 M KNO_3 over the potential window from 0.6 to -0.7 V (*vs.* RHE). (b-d) CA curves (b), FEs(NH_3) and FEs(NO_2^-) (c), and NH_3 yield rates (d) at 0.1, 0, -0.1, -0.2 and -0.3 V (*vs.* RHE) in the 1 M KOH + 1 M KNO_3 electrolyte.

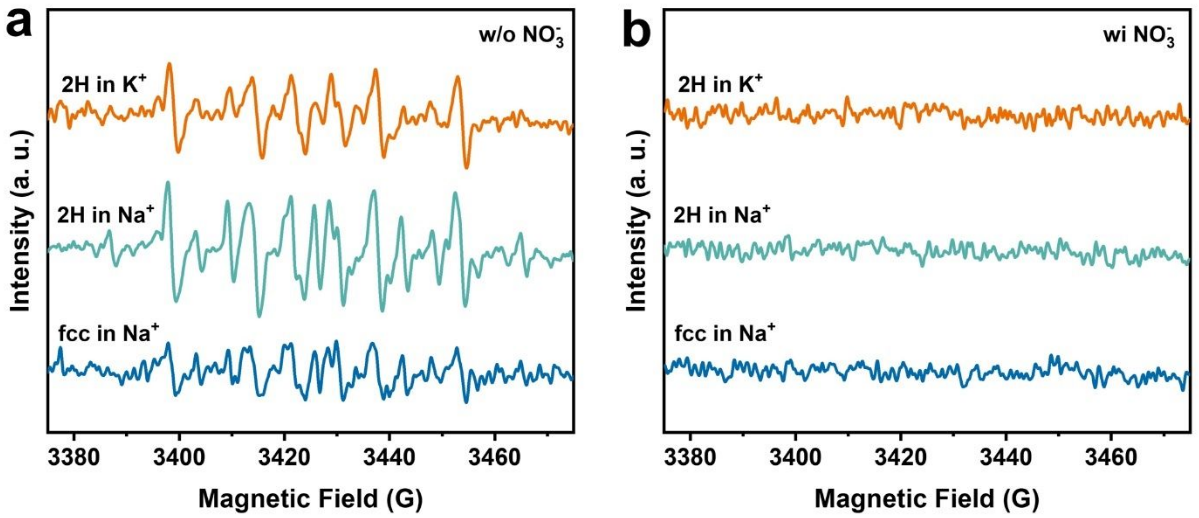

Figure S25. Electrochemical quasi in-situ EPR tests on RhCu nanocatalysts. EPR spectra of fcc-RhCu in Na^+-based solution, 2H-RhCu in Na^+-based solution and 2H-RhCu in K^+-based solution in the absence (a) and presence (b) of 0.1 M NO_3^- at -0.3 V (*vs.* RHE).

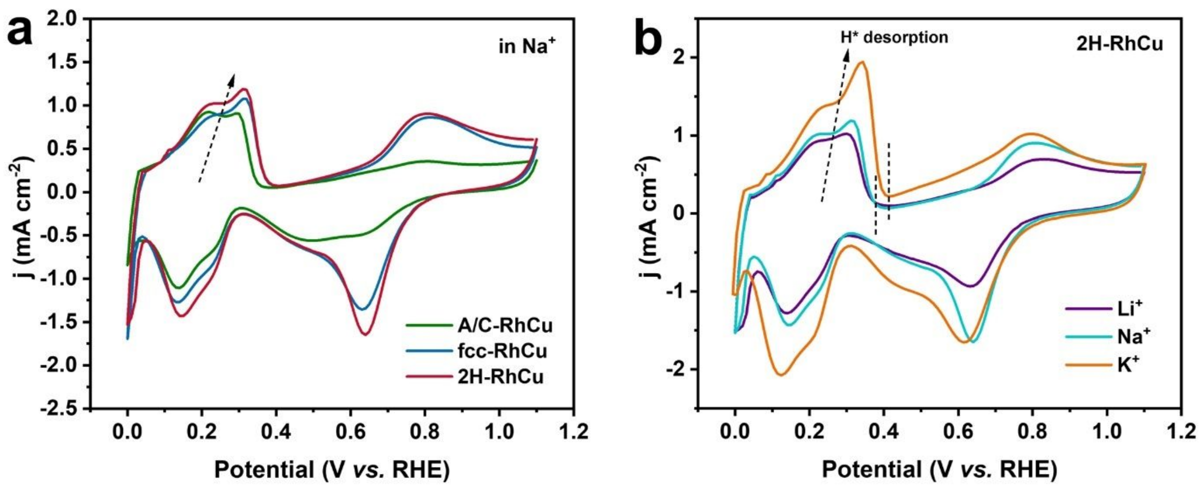

Figure S26. The coverage of active *H on RhCu cathodes in the electrolytes with different cation ions. (a) CV curves obtained on A/C-, fcc- and 2H-RhCu cathodes in 1 M NaOH solution at 5 mV s^{-1} over the potential window of 0 ~ 1.1 V (*vs.* RHE). (b) CV curves obtained on 2H-RhCu cathodes in 1 M LiOH, 1 M NaOH and 1 M KOH solutions at 5 mV s^{-1} over the potential window of 0 ~ 1.1 V (*vs.* RHE).

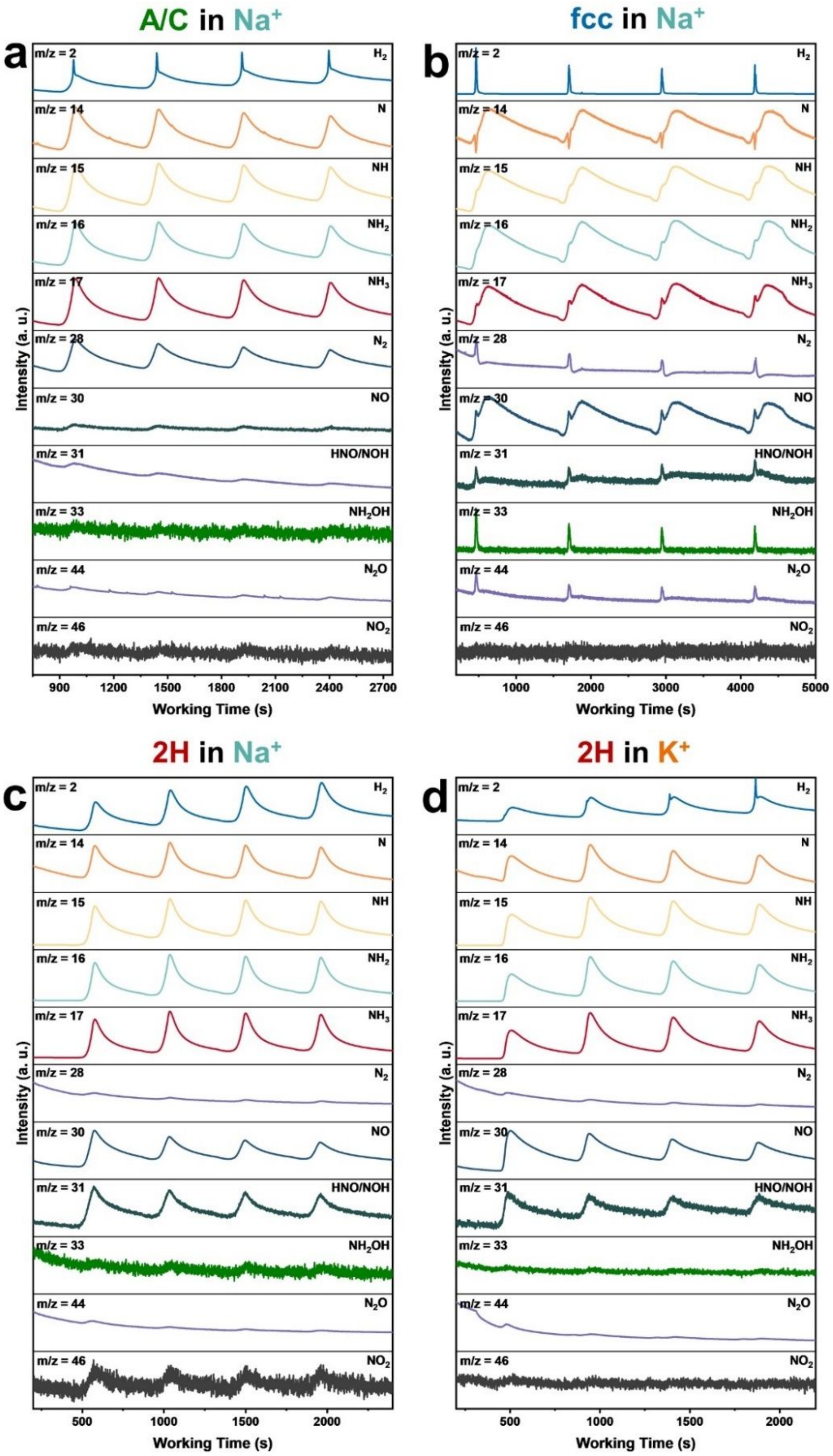

Figure S27. Analysis of water-soluble and gaseous intermediates and end-products of NO_3RR. (a-d) *In-situ* DEMS patterns for A/C- (a), fcc- (b) and 2H-RhCu (c) in 0.5 M Na_2SO_4 + 0.1 M $NaNO_3$ mixed solution and (d) 2H-RhCu in 0.5 M K_2SO_4 + 0.1 M KNO_3 mixed solution during NO_3RR. Four periods of cathodic LSV from 0.2 to -0.7 V (*vs.* RHE) were repeated to improve the detection reliability.

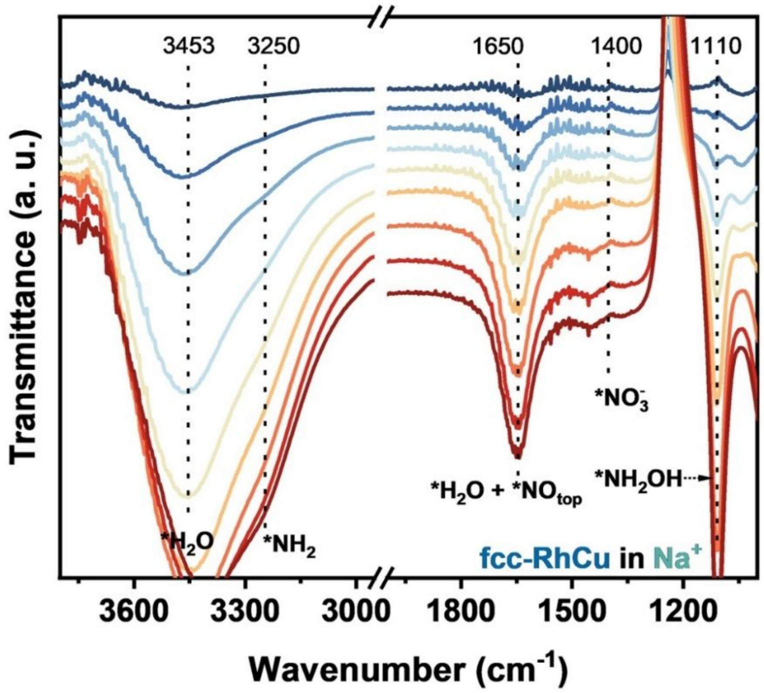

Figure S28. ATR-FTIR spectra of fcc-RhCu in Na^+-based electrolyte. A linear scanning from 0.3 to -0.5 V (vs. RHE) at 2 mV s^{-1} was performed to drive the NO_3RR on as-prepared electrodes in 0.5 M Na_2SO_4 solution containing 0.1 M $NaNO_3$.

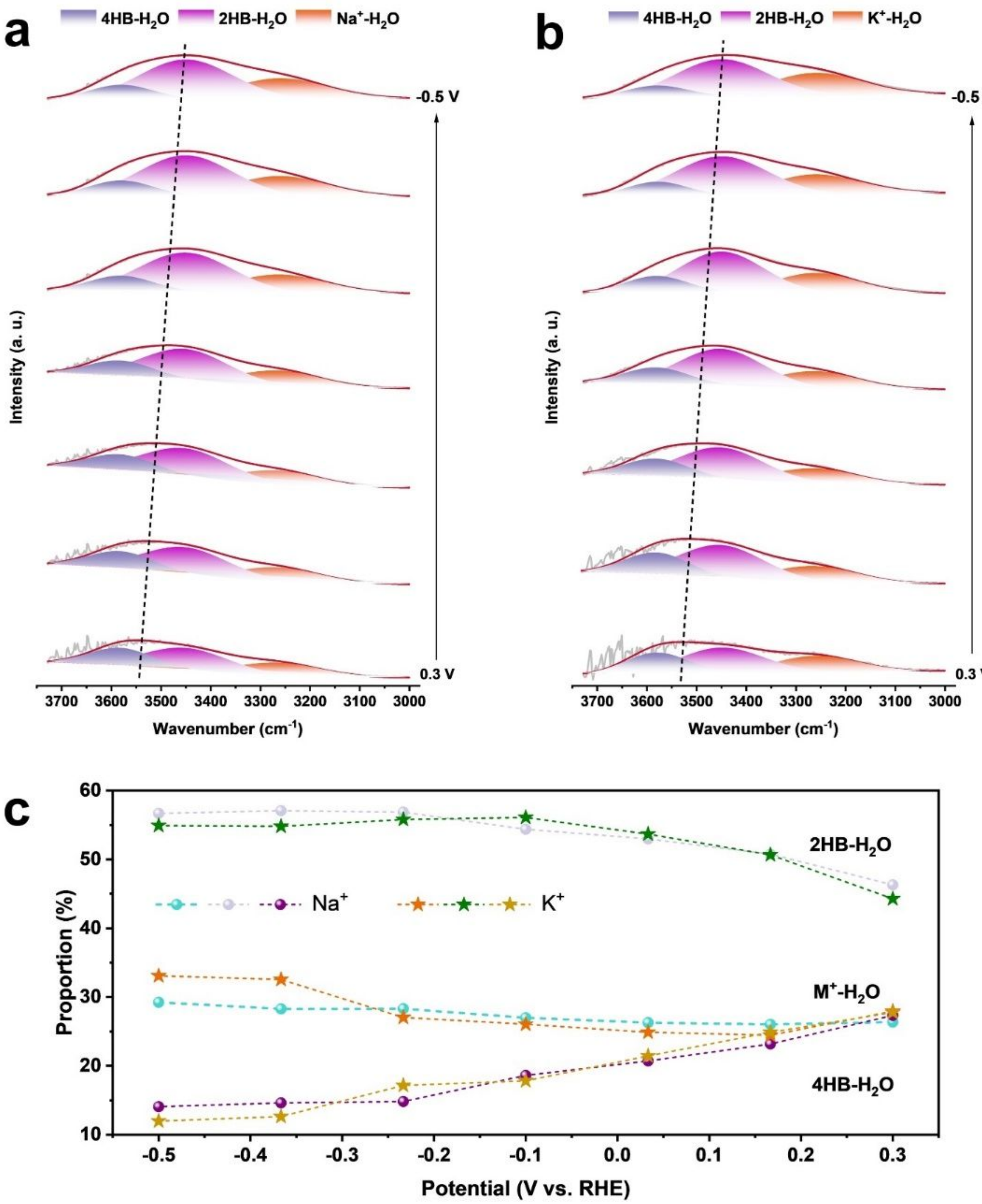

Figure S29. Analysis on the interfacial water structure on 2H-RhCu cathodes in Na^+- and K^+-based electrolytes. (a,b) *In-situ* ATR-FTIR spectra of interfacial water at 2H-RhCu in Na^+- (a) and K^+-based (b) electrolytes. (c) The proportions of metal cation-H_2O, 2HB-H_2O, and 4HB-H_2O solvated structures as the NO_3RR proceeds from 0.3 to -0.5 V (*vs.* RHE).

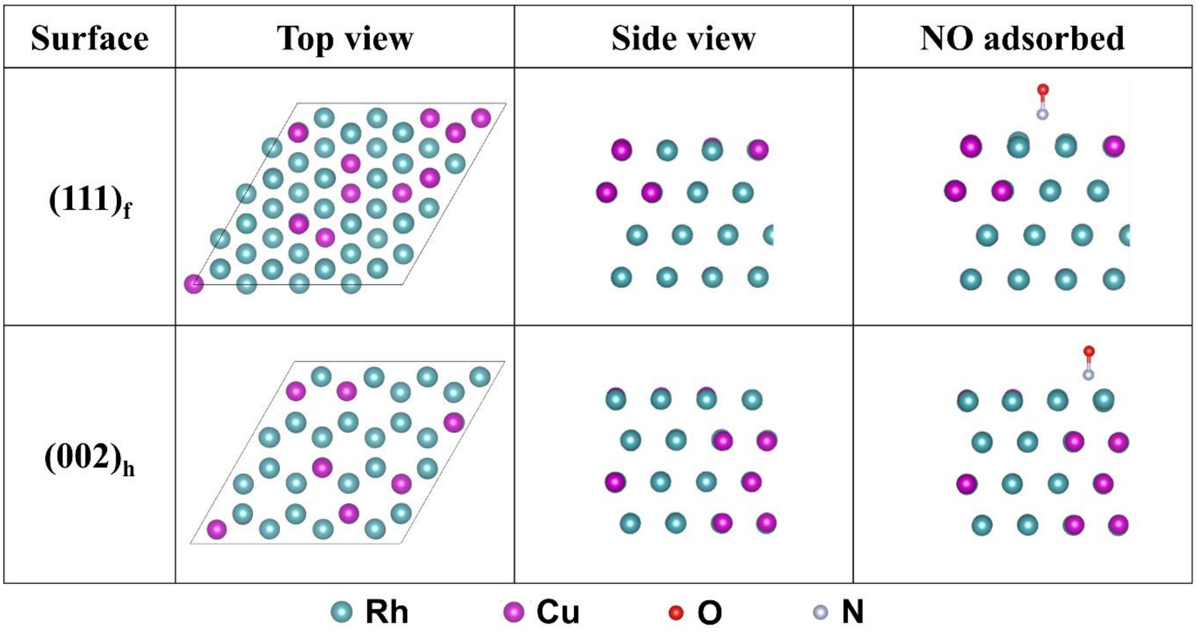

Figure S30. Atomic models of the $(111)_f$ and $(002)_h$ surfaces of RhCu alloys before and after the adsorption of NO. Top view (left panels) and side view (middle panels) of the fcc- and 2H-RhCu atomic models are both shown for better demonstration of their structures. The side view of the fcc- and 2H-RhCu atomic models adsorbed with a NO molecule (right panels) is provided. Cation effects are not considered at this stage.

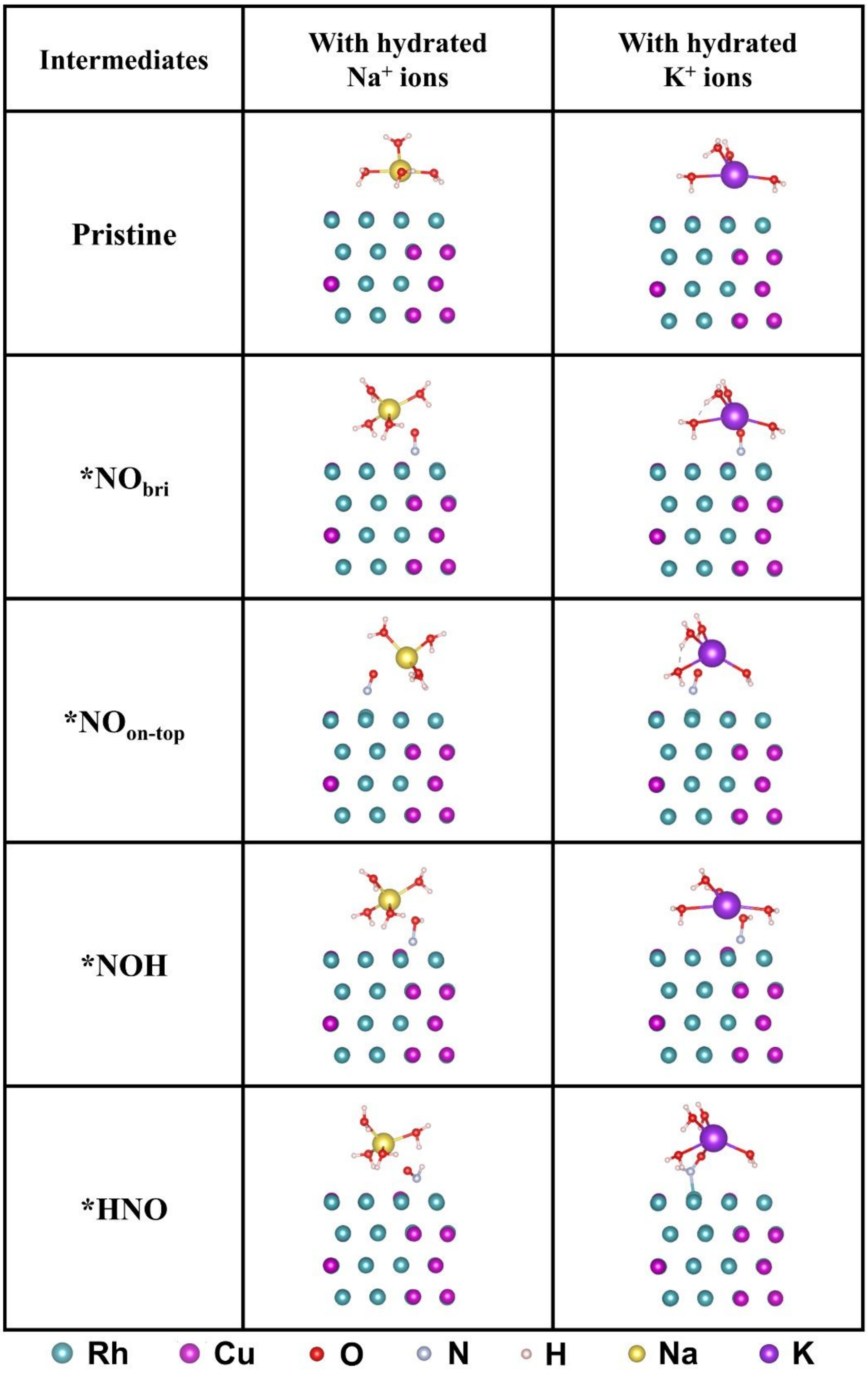

Figure S31. Atomic models of the $(002)_h$ surfaces of RhCu alloys before and after being adsorbed with different intermediates at the critical conversion step from $^*NO$ to $^*NOH/^*HNO$. It provides side views of all these atomic models for direct comparison. A hydrated Na^+ (left panels) or K^+ (right panels) ion is introduced here to simulate the cation effects for NO_3RR.

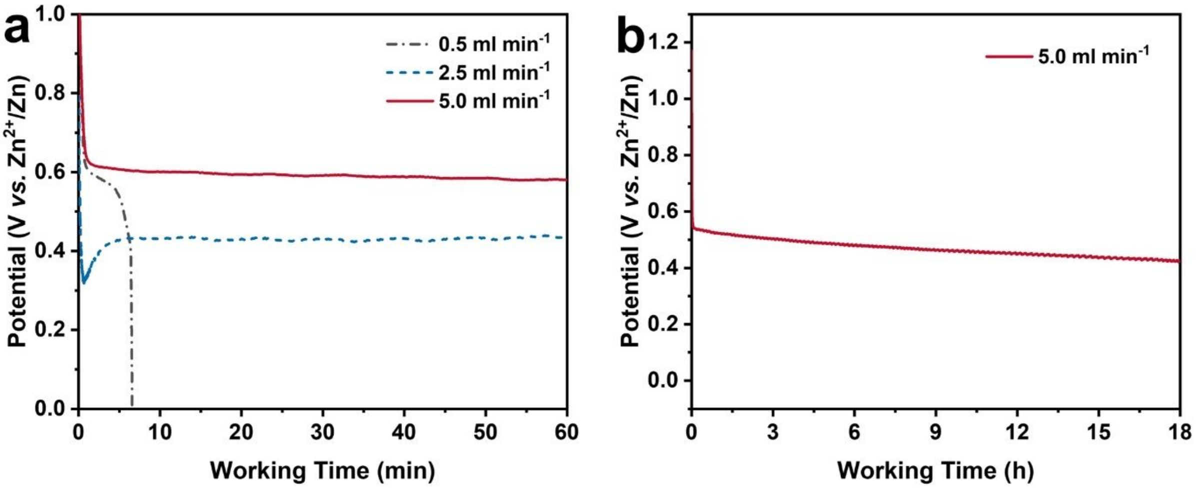

Figure S32. Effects of flow velocities on the working voltages of assembled Zn-nitrate flow batteries. (a) The galvanostatic discharge profiles of Zn-nitrate flow batteries with 2H-RhCu and K^+-based neutral electrolyte at the anolyte/catholyte flow rates of 0.5, 2.5 and 5.0mL min^{-1} within the initial first hour. (b) Working voltage stability evaluation of Zn-nitrate flow batteries with 2H-RhCu and K^+-based neutral electrolyte at the anolyte/catholyte flow rate of 5.0 mL min^{-1}.

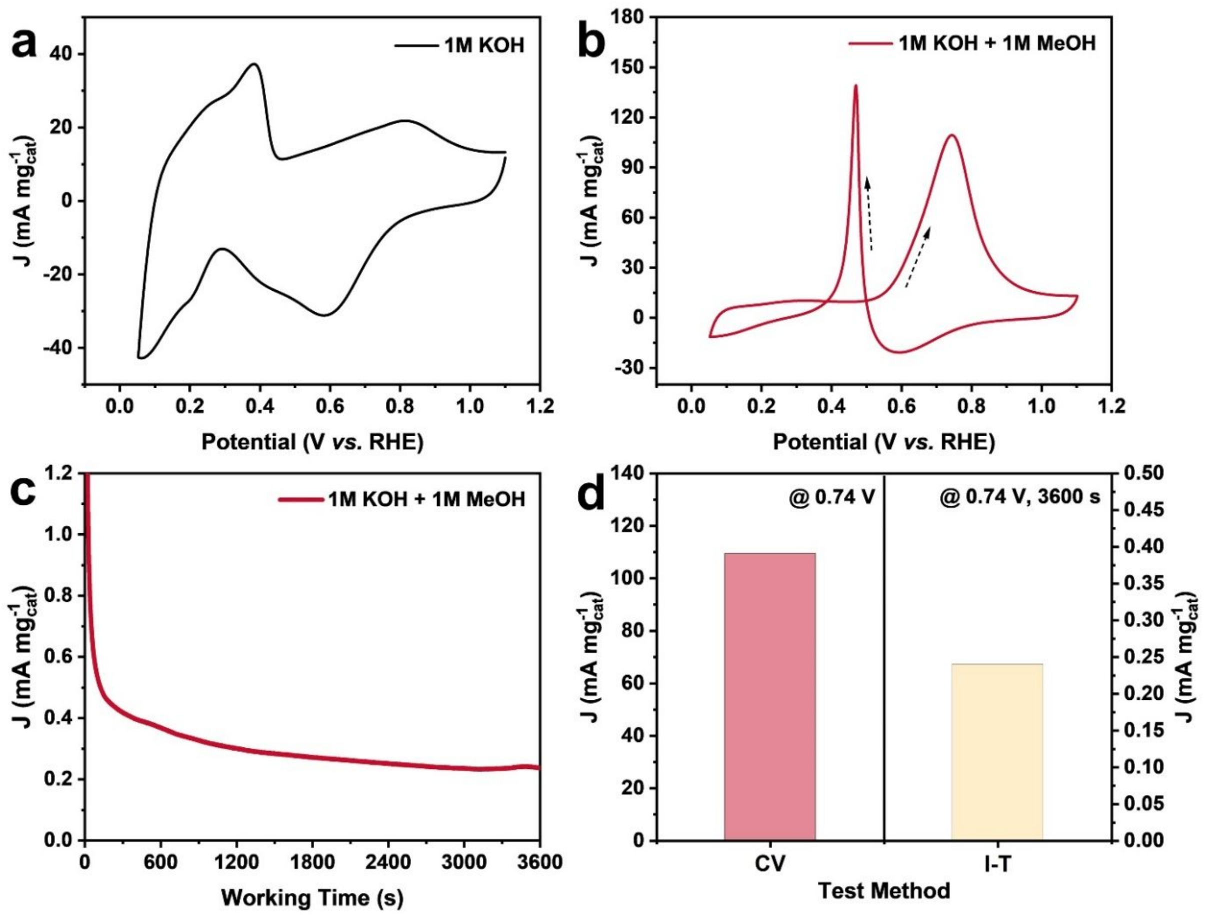

Figure S33. MOR performance of 2H-RhCu. (a,b) CV profiles of 2H-RhCu at 50 mV s^{-1} over the potential window of 0.05 - 1.10 V (*vs.* RHE) in 1 M KOH (a) and 1 M KOH + 1 M MeOH (b) solution. (c) Chronoamperometric curve of 2H-RhCu tested in 1 M KOH + 1 M MeOH at 0.74 V (*vs*. RHE) within 3600 s. (d) Mass activity for 2H-RhCu at 0.74 V (*vs.* RHE) in CV test (left panel) and at 3600 s under 0.74 V (*vs.* RHE) in I-T tests (right panel).

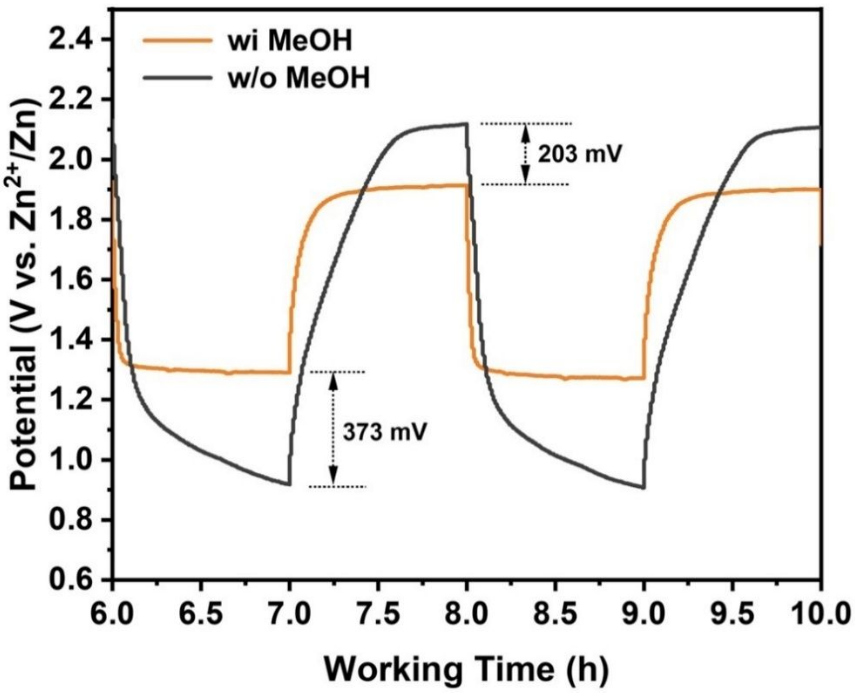

Figure S34. Comparison on the discharge-charge profiles of Zn-nitrate flow batteries with and without methanol in the catholyte. The cathode catalyst is 2H-RhCu while the electrolytes are 0.5 M K_2SO_4 + 0.1 M KNO_3 mixed solutions with or without adding 1 M MeOH. Each cycle is comprised of a galvanostatic discharging process for 1 h and a galvanostatic charging process for 1 h at both 0.1 mA cm^{-2}.

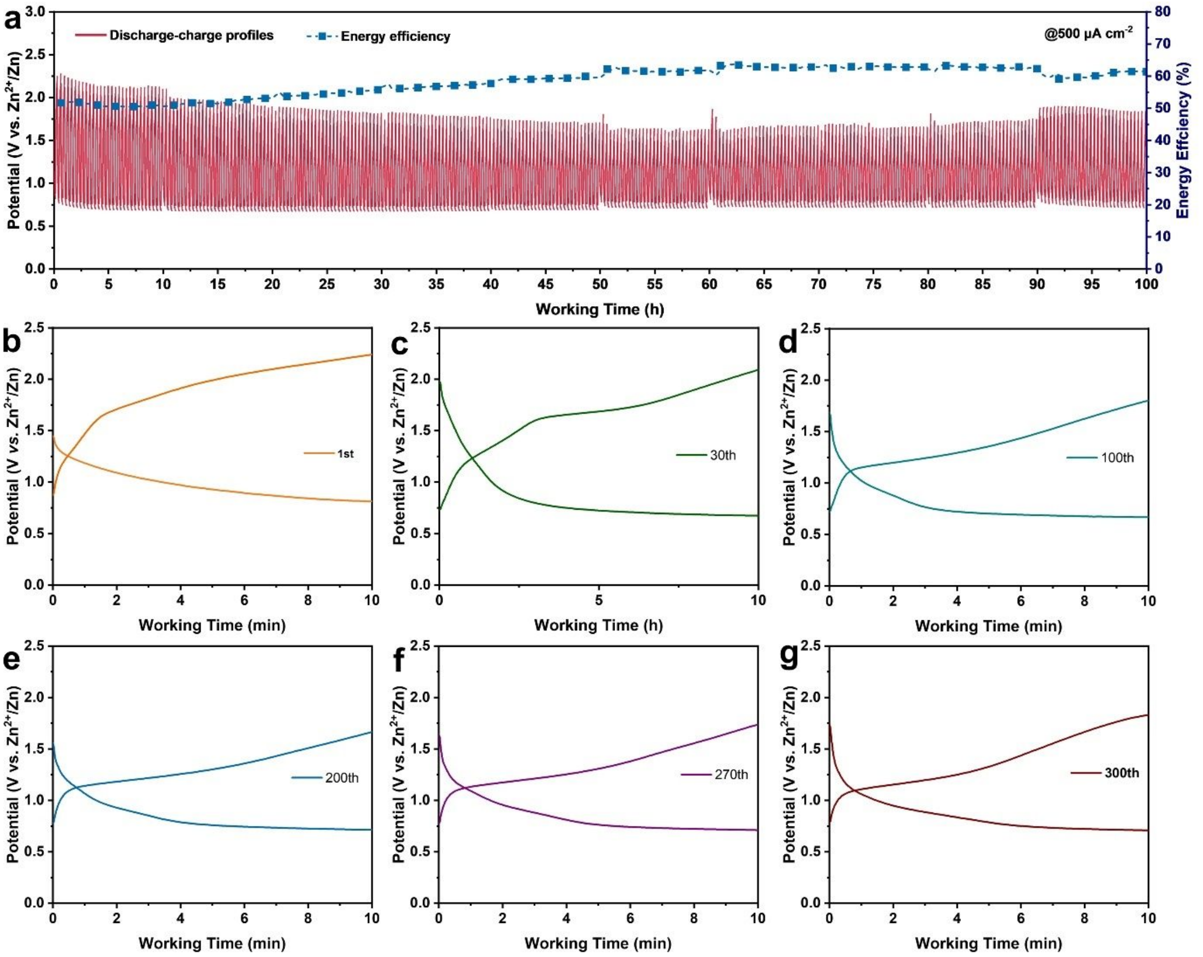

Figure S35. Long-term cycling behaviour of an assembled Zn-NO_3RR/MOR FC battery. (a-g) Woking voltage-time curves and energy efficiencies (a) and discharge-charge profiles at the selected cycles (b-g) of an assembled Zn-NO_3RR/MOR FC battery with 2H-RhCu at 500 μA cm^{-2} within 100 h. Every cycle contains a galvanostatic discharging for 10 min and subsequent charging for 10 min, and the battery could steadily last for at least 300 cycles.

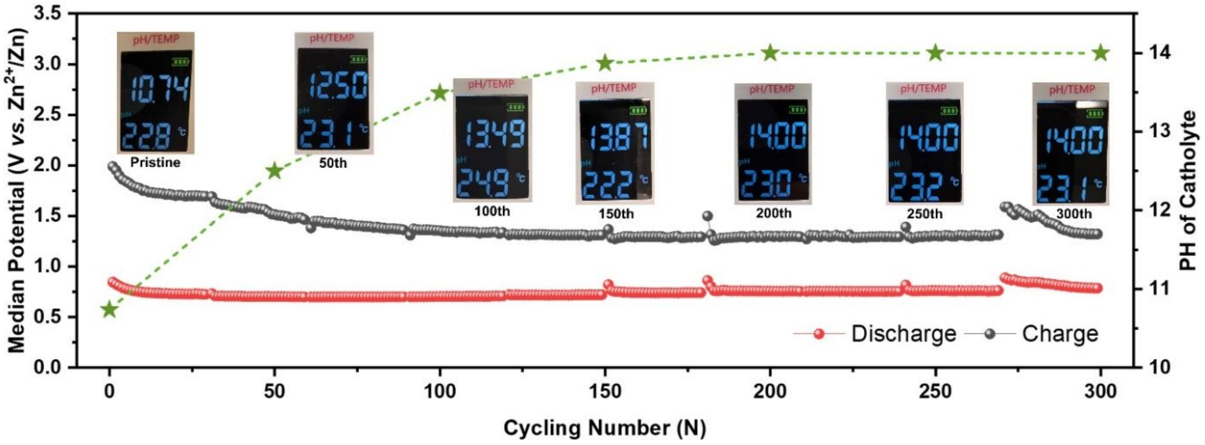

Figure S36. Discharge and charge median potentials and pH change of catholyte of the assembled rechargeable Zn-nitrate/methanol flow batteries within the initial 300 cycles.

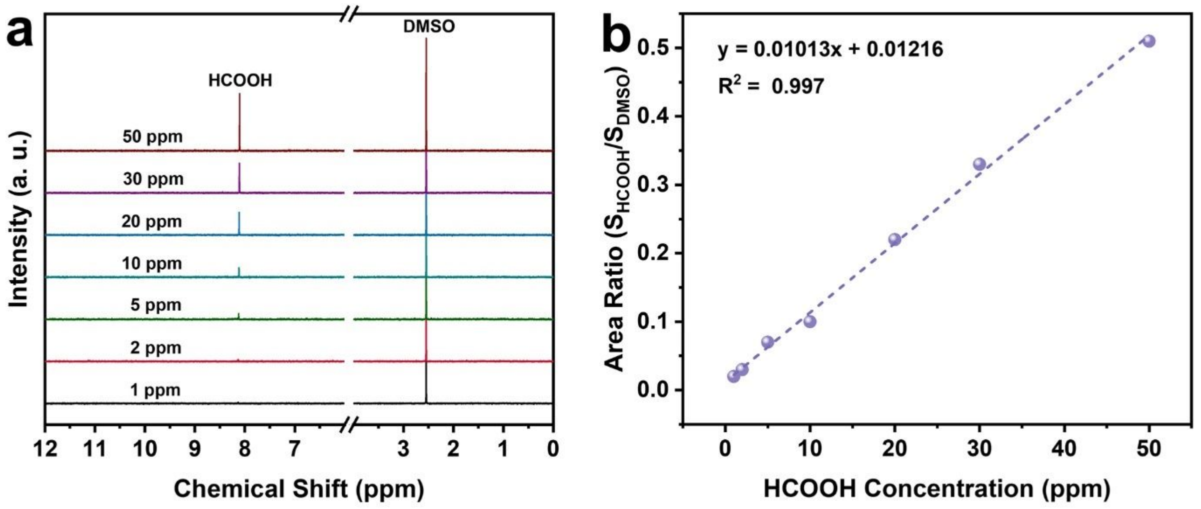

Figure S37. Establishment of the calibration curve of HCOOH concentrations by ^{1}H NMR methods. (a,b) ^{1}H NMR spectra for solutions with HCOOH concentrations of 1, 2, 5, 10, 20, 30 and 50 ppm (a) and calibration curve (b) of the solutions with given HCOOH concentrations. Note that DMSO is used as the internal standard.

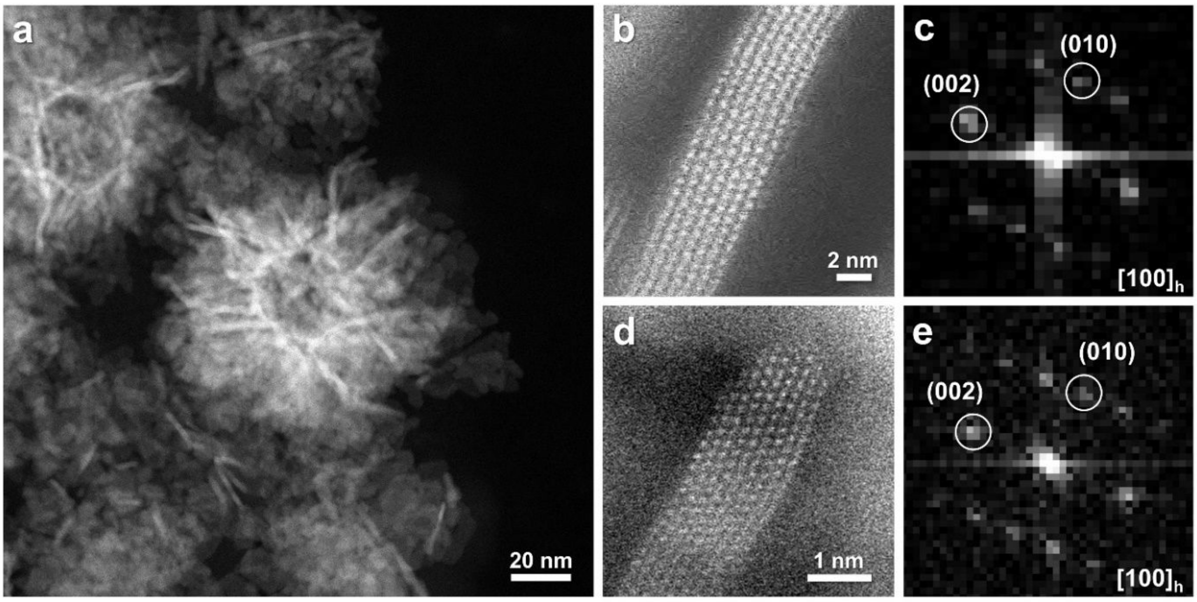

Figure S38. Structural characterization of 2H-RhCu electrocatalyst taken from the cycled Zn-nitrate/methanol flow batteries. (a-e) Low-magnification (a), atomic-resolution (b,d) HAADF-STEM images of 2H-RhCu taken from the cycled flow battery and the FFT patterns (c,e) corresponding to (b) and (d), respectively.

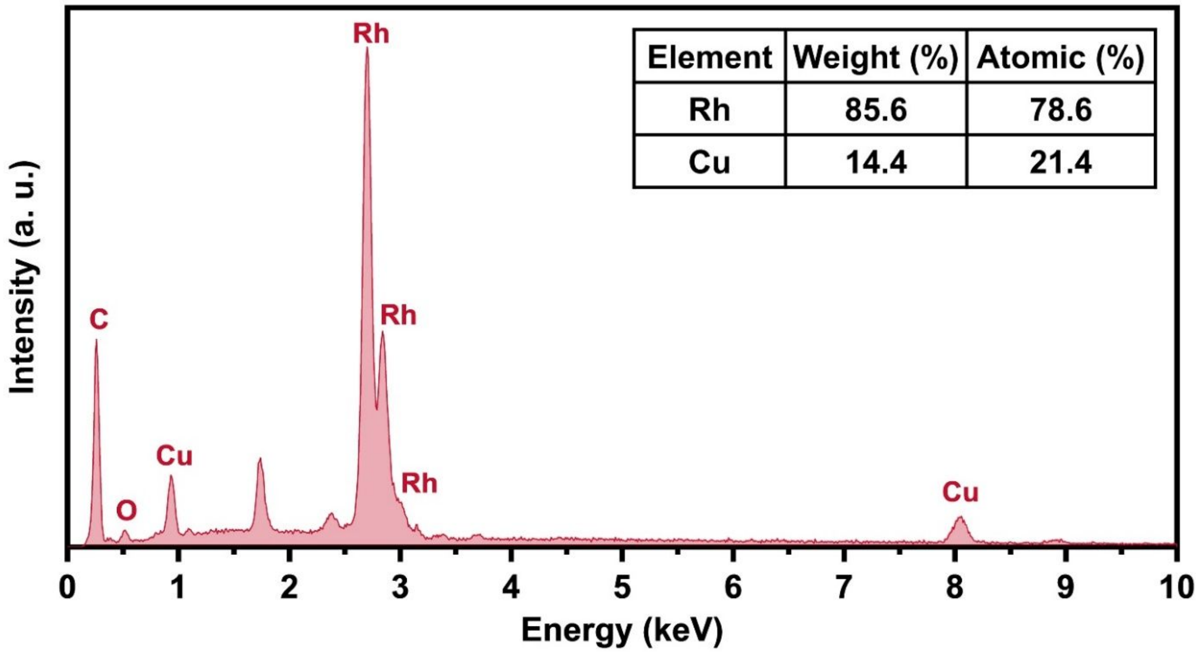

Element	Weight (%)	Atomic (%)
Rh	85.6	78.6
Cu	14.4	21.4

Figure S39. EDS spectrum of the 2H-RhCu cathode taken from the Zn-nitrate/methanol flow battery. Inset: a table showing the elemental ratio between Rh and Cu.

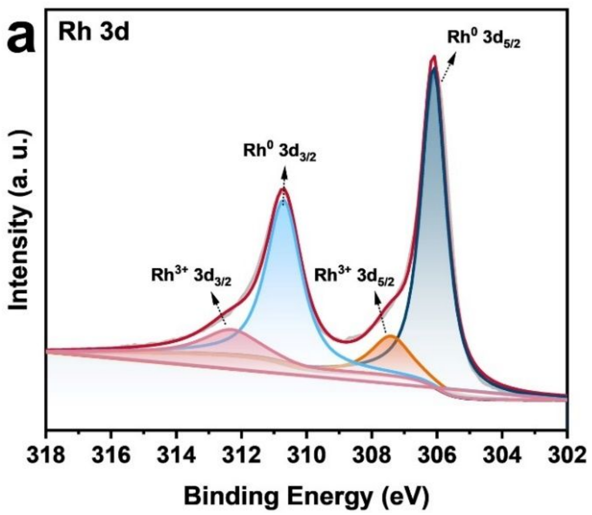

Figure S40. XPS analysis of the 2H-RhCu cathode taken from the Zn-nitrate/methanol flow battery after 300 deep cycles. (a,b) High-resolution Rh 3d (a) and Cu 2p (b) XPS spectra for the 2H-RhCu cathode after the long-term cycling.

3. Supporting Tables

Table S1. The fitting results of Rh K-edge and Cu K-edge EXAFS spectra for 2H-RhCu, fcc-RhCu, Rh foil and Cu foil (S_0^2 = 0.92).

Samples	Element	Scattering path	R (Å)	C.N.	σ^2 (Å^2)	ΔE_0 (eV)	R-factor
**2H-RhCu**	Rh K-edge	Rh-Rh	2.85	2.7	0.009	-5.7	0.02
		Rh-O	1.98	3.3			
	Cu K-edge	Cu-Cu	2.55	9.9	0.009	3.7	0.006
		Cu-Rh	2.60	0.6			
		Cu-O	1.88	0.4			
**fcc-RhCu**	Rh K-edge	Rh-Rh	2.69	4.5	0.005	-6.8	0.018
		Rh-O	1.98	2.1			
	Cu K-edge	Cu-Cu	2.56	8.8	0.012	4.4	0.001
		Cu-Rh	2.69	1.3			
		Cu-O	1.90	2.2			
**Rh foil**	Rh K-edge	Rh-Rh	2.69	12	0.002	-6.4	0.004
**Cu foil**	Cu K-edge	Cu-Cu	2.55	12	0.009	4.5	0.003

Note: Since the loading of Rh is significantly higher than that of Cu, the Rh-Cu bonds are negligible in these bimetallic samples.

Table S2. Comparison on the electrocatalytic performance of 2H-RhCu in K^+-based electrolyte proposed in this work and the other typical electrocatalysts reported previously for NO_3RR in neutral media.

Catalysts	Electrolytes	FE_{NH3} (%)	Potential (V *vs.* RHE)	NH_3 yield rate	Refs.
2H-RhCu	0.5 M K_2SO_4 + 0.1 M KNO_3 (pH = 7)	94.8	-0.3	4936.8 mg h^{-1} g^{-1}_{cat} at -0.4 V	This work
LC-Rh NF	0.1 M Na_2SO_4 + 0.1 M KNO_3 (pH = 11.5)	95	0.2	506.6 mg h^{-1} g^{-1}_{cat} at 0.2 V	5
RhCu M-tpp	0.5 M Na_2SO_4 + 3000 ppm $NaNO_3$ (pH = 7)	84.8	-0.2	717.8 mg h^{-1} g^{-1}_{cat} at -0.5 V	6
PA-RhCu cNCs	0.1 M $HClO_4$ + 0.05 M KNO_3 (pH = 1)	93.7	0.05	2400 mg h^{-1} g^{-1}_{cat} at 0.05 V	7
Rh/C	0.5 M H_2SO_4 + 0.1 M $NaNO_3$ (pH = 0)	92	0.1	N/A	8
PdCu/Cu_2O	0.5 M Na_2SO_4 +1000 ppm NO_3^-	94.32	-0.8	3.23 mg h^{-1} cm^{-2} at -0.8V	9
TiO_{2-x}	0.5 M Na_2SO_4 + 50 ppm $NaNO_3$	85	-0.94	765 mg h^{-1} g^{-1}_{cat} at -1.6 V	10
S-modified Fe SACs	0.02 M Na_2SO_4 + 100 ppm N-NO_3^-	78.4	-0.54	~ 190 mg h^{-1} g^{-1}_{Fe} at -0.87 V	11
Pd-NDs/Zr-MOF	0.1 M Na_2SO_4 + 500 ppm $NaNO_3$	58.1	-1.3	4884.3 mg h^{-1} g^{-1}_{cat} at -1.3 V	12
Co SACs	0.02 M Na_2SO_4 + 100 ppm N-NO_3^-	92	-0.69	0.409 mg h^{-1} cm^{-2} at -0.89 V	13
Plasma-Cu_2O	0.5 M Na_2SO_4 + 200 ppm $NaNO_3$	85.26	-0.6	1.19 mg h^{-1} cm^{-2} at -0.6 V	14
Cu-rich PdCu	0.5 M K_2SO_4 + 50 ppm N-KNO_3	90.02	-0.46	784.37 mg h^{-1} g^{-1}_{cat} at -0.46V	15
O-Cu-PTCDA	0.1M PBS+ 500 ppm NO_3^-	85.9	-0.4	0.875 mg h^{-1} cm^{-2} at -0.6 V	16
Cu-cis-N_2O_2	0.5 M Na_2SO_4 + 1000 ppm KNO_3	86	-1.2	27.84 mg h^{-1} cm^{-2} at -2.0 V	17
Cu-GS-1000	0.1 M K_2SO_4 + 0.1 M KNO_3	98	-0.8	3000 mg h^{-1} g^{-1}_{cat} at -0.8 V	18
Cu/Cu_2O NWAs	0.5 M Na_2SO_4 + 200 ppm $NaNO_3$	95.8	-0.85	4.1633 mg h^{-1} cm^{-2} at -0.85 V	19
Ni_1Cu-SAA	0.5 M K_2SO_4 + 200 ppm KNO_3	98	-0.55	7.14 mg h^{-1} cm^{-2} at -0.75 V	20

Fe/Ni_2P	0.2 M K_2SO_4 + 50 mM KNO_3	94.3	-0.4	6.5 mg h^{-1} cm^{-2} at -0.6 V	21
TiO_2 NTs/CuO_x	0.5 M Na_2SO_4 + 100 ppm N-KNO_3	92.2	-0.75	1.24 mg h^{-1} cm^{-2} at -0.75 V	22
Fe SACs	0.1 M K_2SO_4 + 0.5 M KNO_3	75	-0.66	~ 20000 mg h^{-1} g^{-1}_{cat} at -0.85 V	23
SN Co-Li^+/PCNF	0.5 M Na_2SO_4 + 0.5 M KNO_3	72.7	-0.94	12.75 mg h^{-1} cm^{-2} at -1.04 V	24
Pd/TiO_2	1 M LiCl + 0.25 M $LiNO_3$	92.1	-0.7	1.12 mg h^{-1} cm^{-2} at -0.8 V	25
Cu@$Cu_{2+1}O$ NWs	0.5 M K_2SO_4 + 50 ppm KNO_3	87.07	-0.55	630 mg h^{-1} g^{-1}_{cat} at -0.84 V	26

Note: From Table S2, 2H-RhCu can exhibit the unique capability of realizing sufficient FEs(NH_3) at much less negative potentials (e.g., > -0.3 V (*vs.* RHE)) and its NH_3 yield rate in K^+-based electrolyte is comparable to those for some transition metal oxides. However, those single-atom catalysts take an obvious leading position when evaluating the NH_3 yield rate only based on the weight of metal elements, but we think that the substrate effect cannot be ignored in NO_3RR processes, despite that the bare substrates like carbon and inert metal oxides have been proven to be with negligible electroactivity for NH_3 generation.

Table S3. Adsorption energies of NO on RhCu $(111)_f$ and $(002)_h$ surfaces and the adsorption energies of NO_{bri} and $NO_{on\text{-}top}$ on RhCu $(002)_h$ surface after considering cation effects.

Species	Surfaces	E_{ads} (eV)	Cations
*NO	$(111)_f$	-2.721	No hydrated ions
*NO	$(002)_h$	-3.124	No hydrated ions
$^*NO_{bri}$	$(002)_h$	-2.894	Hydrated Na^+ ions
$^*NO_{on\text{-}top}$	$(002)_h$	-2.434	Hydrated Na^+ ions
$^*NO_{bri}$	$(002)_h$	-2.832	Hydrated K^+ ions
$^*NO_{on\text{-}top}$	$(002)_h$	-2.301	Hydrated K^+ ions

Table S4. Gibbs free energy evolution at the intermediate conversion step from $^*NO$ to $^*NOH$/$^*HNO$ on the established $(002)_h$ surface of RhCu alloy in Na^+ and K^+ based solutions.

**Initial state**	**End state**	**Surfaces**	**ΔG (eV)**	**Cations**
**$^*NO$**	$^*NOH$	$(002)_h$	-0.202	Hydrated Na^+ ions
**$^*NO$**	$^*HNO$	$(002)_h$	-0.310	Hydrated Na^+ ions
**$^*NO$**	$^*NOH$	$(002)_h$	-0.384	Hydrated K^+ ions
**$^*NO$**	$^*HNO$	$(002)_h$	-0.136	Hydrated K^+ ions

References

(1) Hafner, J., Ab-initio simulations of materials using vasp: Density-functional theory and beyond. *J. Comput. Chem.* **2008,** *29* (13), 2044-78.

(2) Blochl, P. E., Projector augmented-wave method. *Phys. Rev. B* **1994,** *50* (24), 17953-17979.

(3) Du, Y.; Zhu, Y.; Xi, S.; Yang, P.; Moser, H. O.; Breese, M. B.; Borgna, A., Xafca: A new xafs beamline for catalysis research. *J. Synchrotron Radiat.* **2015,** *22* (3), 839-43.

(4) Newville, M., Ifeffit: Interactive xafs analysis and feff fitting. *J. Synchrotron Radiat.* **2001,** *8*, 322-324.

(5) Liu, H.; Timoshenko, J.; Bai, L.; Li, Q.; Rüscher, M.; Sun, C.; Roldan Cuenya, B.; Luo, J., Low-coordination rhodium catalysts for an efficient electrochemical nitrate reduction to ammonia. *ACS Catal.* **2023,** *13* (2), 1513-1521.

(6) Zhou, J.; Xiong, Y.; Sun, M.; Xu, Z.; Wang, Y.; Lu, P.; Liu, F.; Hao, F.; Feng, T.; Ma, Y.; Yin, J.; Ye, C.; Chen, B.; Xi, S.; Zhu, Y.; Huang, B.; Fan, Z., Constructing molecule-metal relay catalysis over heterophase metallene for high-performance rechargeable zinc-nitrate/ethanol batteries. *Proc. Natl. Acad. Sci.* **2023,** *120* (50), e2311149120.

(7) Ge, Z. X.; Wang, T. J.; Ding, Y.; Yin, S. B.; Li, F. M.; Chen, P.; Chen, Y., Interfacial engineering enhances the electroactivity of frame-like concave RhCu bimetallic nanocubes for nitrate reduction. *Adv. Energy Mater.* **2022,** *12* (15), 2103916.

(8) Richards, D.; Young, S. D.; Goldsmith, B. R.; Singh, N., Electrocatalytic nitrate reduction on rhodium sulfide compared to Pt and Rh in the presence of chloride. *Catal. Sci. Technol.* **2021,** *11* (22), 7331-7346.

(9) Yin, H.; Chen, Z.; Xiong, S.; Chen, J.; Wang, C.; Wang, R.; Kuwahara, Y.; Luo, J.; Yamashita, H.; Peng, Y.; Li, J., Alloying effect-induced electron polarization drives nitrate electroreduction to ammonia. *Chem Catal.* **2021,** *1* (5), 1088-1103.

(10) Jia, R.; Wang, Y.; Wang, C.; Ling, Y.; Yu, Y.; Zhang, B., Boosting selective nitrate electroreduction to ammonium by constructing oxygen vacancies in TiO_2. *ACS Catal.* **2020,** *10* (6), 3533-3540.

(11) Li, J.; Li, M.; An, N.; Zhang, S.; Song, Q.; Yang, Y.; Liu, X., Atomically dispersed Fe atoms anchored on S and N-codoped carbon for efficient electrochemical denitrification. *Proc. Natl. Acad. Sci.* **2021,** *118* (33), e2105628118.

(12) Jiang, M.; Su, J.; Song, X.; Zhang, P.; Zhu, M.; Qin, L.; Tie, Z.; Zuo, J. L.; Jin, Z., Interfacial reduction nucleation of noble metal nanodots on redox-active metal-organic frameworks for high-efficiency electrocatalytic conversion of nitrate to ammonia. *Nano Lett.* **2022,** *22* (6), 2529-2537.

(13) Li, J.; Li, M.; An, N.; Zhang, S.; Song, Q.; Yang, Y.; Li, J.; Liu, X., Boosted ammonium production by single cobalt atom catalysts with high faradic efficiencies. *Proc. Natl. Acad. Sci.* **2022,** *119* (29), e2123450119.

(14) Gong, Z.; Zhong, W.; He, Z.; Liu, Q.; Chen, H.; Zhou, D.; Zhang, N.; Kang, X.; Chen, Y., Regulating surface oxygen species on copper (I) oxides via plasma treatment for effective reduction of nitrate to ammonia. *Appl. Catal. B* **2022,** *305*, 121021.

(15) Xu, Y.; Ren, K.; Ren, T.; Wang, M.; Liu, M.; Wang, Z.; Li, X.; Wang, L.; Wang, H., Cooperativity of Cu and Pd active sites in cupd aerogels enhances nitrate electroreduction to ammonia. *Chem. Commun.* **2021,** *57* (61), 7525-7528.

(16) Chen, G.-F.; Yuan, Y.; Jiang, H.; Ren, S.-Y.; Ding, L.-X.; Ma, L.; Wu, T.; Lu, J.; Wang, H., Electrochemical reduction of nitrate to ammonia via direct eight-electron transfer using a copper-molecular solid catalyst. *Nat. Energy* **2020,** *5* (8), 605-613.

(17) Cheng, X. F.; He, J. H.; Ji, H. Q.; Zhang, H. Y.; Cao, Q.; Sun, W. J.; Yan, C. L.; Lu, J. M., Coordination symmetry breaking of single-atom catalysts for robust and efficient nitrate electroreduction to ammonia. *Adv. Mater.* **2022,** *34* (36), 2205767.

(18) Leverett, J.; Tran-Phu, T.; Yuwono, J. A.; Kumar, P.; Kim, C.; Zhai, Q.; Han, C.; Qu, J.; Cairney, J.; Simonov, A. N.; Hocking, R. K.; Dai, L.; Daiyan, R.; Amal, R., Tuning the coordination structure of Cu-N-C single atom catalysts for simultaneous electrochemical reduction of CO_2 and no_3^- to urea. *Adv. Energy Mater.* **2022,** *12* (32), 2201500.

(19) Wang, Y.; Zhou, W.; Jia, R.; Yu, Y.; Zhang, B., Unveiling the activity origin of a copper-based electrocatalyst for selective nitrate reduction to ammonia. *Angew. Chem. Int. Ed.* **2020,** *59* (13), 5350-5354.

(20) Cai, J.; Wei, Y.; Cao, A.; Huang, J.; Jiang, Z.; Lu, S.; Zang, S.-Q., Electrocatalytic nitrate-to-ammonia conversion with ~100% faradaic efficiency via single-atom alloying. *Appl. Catal. B* **2022,** *316*, 121683.

(21) Zhang, R.; Guo, Y.; Zhang, S.; Chen, D.; Zhao, Y.; Huang, Z.; Ma, L.; Li, P.; Yang, Q.; Liang, G.; Zhi, C., Efficient ammonia electrosynthesis and energy conversion through a Zn-nitrate battery by iron doping engineered nickel phosphide catalyst. *Adv. Energy Mater.* **2022,** *12* (13), 2103872.

(22) Qiu, W.; Chen, X.; Liu, Y.; Xiao, D.; Wang, P.; Li, R.; Liu, K.; Jin, Z.; Li, P., Confining intermediates within a catalytic nanoreactor facilitates nitrate-to-ammonia electrosynthesis. *Appl. Catal. B* **2022,** *315*, 121548.

(23) Wu, Z. Y.; Karamad, M.; Yong, X.; Huang, Q.; Cullen, D. A.; Zhu, P.; Xia, C.; Xiao, Q.; Shakouri, M.; Chen, F. Y.; Kim, J. Y. T.; Xia, Y.; Heck, K.; Hu, Y.; Wong, M. S.; Li, Q.; Gates, I.; Siahrostami, S.; Wang, H., Electrochemical ammonia synthesis via nitrate reduction on Fe single atom catalyst. *Nat. Commun.* **2021,** *12* (1), 2870.

(24) Mi, L.; Huo, Q.; Cao, J.; Chen, X.; Yang, H.; Hu, Q.; He, C., Achieving synchronization of electrochemical production of ammonia from nitrate and ammonia capture by constructing a "two-in-one" flow cell electrolyzer. *Adv. Energy Mater.* **2022,** *12* (44), 2202247.

(25) Guo, Y.; Zhang, R.; Zhang, S.; Zhao, Y.; Yang, Q.; Huang, Z.; Dong, B.; Zhi, C., Pd doping-weakened intermediate adsorption to promote electrocatalytic nitrate reduction on TiO_2 nanoarrays for ammonia production and energy supply with zinc-nitrate batteries. *Energy Environ. Sci.* **2021,** *14*, 3938-3944.

(26) Ren, T.; Ren, K.; Wang, M.; Liu, M.; Wang, Z.; Wang, H.; Li, X.; Wang, L.; Xu, Y., Concave-convex surface oxide layers over copper nanowires boost electrochemical nitrate-to-ammonia conversion. *Chem. Eng. J.* **2021,** *426*, 130759.
